# In silico profiling for secondary metabolites from *Lepidium meyenii* (maca) by the pharmacophore and ligand-shape-based joint approach

**DOI:** 10.1186/s13020-016-0112-y

**Published:** 2016-09-26

**Authors:** Fan Yi, Xiao-lei Tan, Xin Yan, Hai-bo Liu

**Affiliations:** 1 Institute of Medicinal Plant Development, Chinese Academy of Medical Sciences, Peking Union Medical College, 151 Malianwa N, Haidian District, Beijing, 100193 China; 2 Key Laboratory of Bioactive Substances and Resources Utilization of Chinese Herbal Medicine, Ministry of Education, Beijing, 100193 China; 3 Research Center for Drug Discovery, School of Pharmaceutical Sciences, Sun Yat-sen University, 132 East Circle at University City, Guangzhou, 510006 China

## Abstract

**Background:**

*Lepidium meyenii* Walpers (maca) is an herb known as a traditional nutritional supplement and widely used in Peru, North America, and Europe to enhance human fertility and treat osteoporosis. The secondary metabolites of maca, namely, maca alkaloids, macaenes, and macamides, are bioactive compounds, but their targets are undefined.

**Methods:**

The pharmacophore-based PharmaDB targets database screening joint the ligand shape similarity-based WEGA validation approach is proposed to predict the targets of these unique constituents and was performed using Discovery Studio 4.5 and PharmaDB. A compounds–targets–diseases network was established using Cytoscape 3.2. These suitable targets and their genes were calculated and analyzed using ingenuity pathway analysis and GeneMANIA.

**Results:**

Certain targets were identified in osteoporosis (8 targets), prostate cancer (9 targets), and kidney diseases (11 targets). This was the first study to identify the targets of these bioactive compounds in maca for cardiovascular diseases (29 targets). The compound with the most targets (46) was an amide alkaloid (MA-24).

**Conclusion:**

In silico target fishing identified maca’s traditional effects on treatment and prevention of osteoporosis, prostate cancer, and kidney diseases, and its potential function of treating cardiovascular diseases, as the most important of this herb’s possible activities.

**Electronic supplementary material:**

The online version of this article (doi:10.1186/s13020-016-0112-y) contains supplementary material, which is available to authorized users.

## Background

*Lepidium meyenii* Walpers (maca) belongs to the brassica (mustard) family and the *Lepidium* genus, which grows robustly only at altitudes over 4000 m [1]. Maca has three major phenotypes, yellow, red and black, based on its hypocotyl and stem coloration [2]. The underground part of the maca is consumed as a food and as a folk medicine to enhance fertility and sexual behaviors and has multiple bioactivities [3]. Currently, maca is used in nutrition and health care products sold from Peru to North America and Europe [4, 5]. Maca contains abundant valuable nutritional ingredients [6], such as maca alkaloids, macaenes, glucosinolates, sterols, and polyphenols, and other secondary metabolites. The maca alkaloids, especially macamides and macaenes, are the main functional constituents of maca [7, 8]. To date, 31 maca alkaloids and four macaenes have been isolated from *L. meyenii*. Their structures are shown below (Table 1). All of the macamides, which are found only in maca, are *N*-benzylamides. Wu [9] synthesized 11 of the reported macamides as well as a series of structurally related amides that resemble macamides (Table 2). These synthesized compounds were collected in this study and used in our experiments.

**Table 1:** Structures of alkaloids and macaenes isolated from *L. meyenii*

No.	Type	Structure	Reference
MA-1	Amide alkaloid		[26]
MA-2	Amide alkaloid		[25]
MA-3	Amide alkaloid		[25]
MA-5	Amide alkaloid		[26]
MA-6	Amide alkaloid		[27]
MA-7	Amide alkaloid		[27]
MA-8	Amide alkaloid		[27]
MA-9	Amide alkaloid		[27]
MA-10	Amide alkaloid		[27]
MA-11	Amide alkaloid		[27]
MA-12	Amide alkaloid		[27]
MA-13	Amide alkaloid		[27]
MA-14	Amide alkaloid		[27]
MA-15	Amide alkaloid		[27]
MA-19	Amide alkaloid		[26]
MA-20	Amide alkaloid		[26]
MA-21	Amide alkaloid		[26]
MA-22	Amide alkaloid		[26]
MA-23	Amide alkaloid		[27]
MA-27	Amide alkaloid		[26]
MA-28	Amide alkaloid		[27]
MA-24	Amide alkaloid		[9]
MA-25	Amide alkaloid		[9]
MA-26	Amide alkaloid		[9]
MA-4	Pyridine derivatives		[26]
MA-29	Pyridine derivatives		[28]
MA-31	Pyridine derivatives		[28]
MA-16	Imidazole alkaloid		[25]
MA-17	Imidazole alkaloid		[6]
MA-18	β-carboline alkaloids		[29]
MA-30	Indole alkaloid		[29]
MA-32	Macaene		[30]
MA-33	Macaene		
MA-34	Macaene		
MA-35	Macaene

**Table 2:** Structures of the synthetic amides resembling macamides

No.	Type of compounds	Structure
MA-36	Amide alkaloid	
MA-37	Amide alkaloid	
MA-38	Amide alkaloid	
MA-39	Amide alkaloid	
MA-40	Amide alkaloid	
MA-41	Amide alkaloid	
MA-42	Amide alkaloid	
MA-43	Amide alkaloid	
MA-44	Amide alkaloid	
MA-45	Amide alkaloid	
MA-46	Amide alkaloid	
MA-47	Amide alkaloid	

The biological and pharmacological effects of maca have been investigated in experimental animals such as rats [10], mice [11], fish [12], and bulls [13]. Maca can enhance sexual behavior and increase sperm count [14], improve prostate function related to testosterone enanthate (TE) levels [15], and improve the quality of embryos [16, 17]. Maca also has beneficial effects on learning and memory in scopolamine-induced memory impairment mice [18]. Moreover, maca aqueous extract scavenges free radicals and protects cells against oxidative stress [5].

Several in vitro/in vivo animal experimental studies have shown that secondary metabolites extracted from maca have bioactivities that help treat osteoporosis and enhance prostate function and sexual function [19–21]. However, most in vitro and in vivo experiments have not specified the molecular target of these secondary metabolites and the mechanisms of the functions of the compounds obtained from maca are unclear. The pharmacophore model is reliable for parallel screening to predict and mimic the binding situation of compounds and targets [22, 23]. This study aimed to investigate the network involved in the mechanisms of action of secondary metabolites of maca. We used the pharmacophore-based method in combination with a novel ligand shape similarity strategy and used the weighted Gaussian algorithm (WEGA).

## Methods

### Collection of chemical constituents

The natural constituents of maca were collected from the literature [9, 24–30] using the search terms “*lepidium* or maca” combined with “constitutes, compounds, chemical or metabolites.” Traditionally used maca contains a dominant pattern of secondary metabolites, particularly alkaloids and macaenes [31]. The secondary metabolite constituents from maca were evaluated to precisely predict the active compounds. A total of 47 alkaloids extracted from maca and synthetic amides were categorized into classes.

### Conformer generation

All chemical structures were prepared in SD format, converted from a 2D cdx file format to 3D models, using Open Babel GUI [32] version 2.3.2 (OpenBableGUI; Chris Morley, USA). Molecular energy was minimized using the Energy Minimization module of Discovery Studio version 4.5 (DS 4.5; Accelrys Inc., San Diego, CA, USA) under the chemistry at Harvard Macromolecular Mechanics (CHARMM) force field. This survey led to the construction of the 3D multi-conformational maca compounds molecular structure database (i.e., maca-DB), which was generated by a Monte Carlo-based conformational analysis (FAST mode). These compounds are rigid; the number of conformers for each compound is much less than 255. The maca-DB contains a total of 47 constituents and 9976 conformations.

### Pharmacophore model collection

In silico profiling of the maca-DB was performed using the generated 3D chemical feature-based pharmacophore models. The pharmacophore models were used to represent the binding mode of particular compounds to specific drug targets [33]. Each pharmacophore model contained several convictive chemical features that determine the chemical functionalities of a certain ligand: H-bond donors or acceptors, hydrophobic groups, aromatic nuclei, and positive or negative ionizable moieties [34]. Unlike common docking methods, pharmacophore-based virtual screening outlines the specific compounds and their multiple pharmacologic targets and determines novel actions of these compounds.

### PharmaDB

PharmaDB is the only pharmacophore database implemented in DS 4.5. A total of 68,000 pharmacophores were derived from 8000 protein–ligand complexes in the sc-PDB dataset. sc-PDB is designed to identify binding sites suitable for the docking of a drug-like ligand, and 9276 three-dimensional structures of binding sites were identified using the Protein Data Bank (PDB) [35].

### Parameters

In this study, PharmaDB was used for profiling. All pharmacophore models with the shape of the binding pocket were selected for virtual screening using the default settings of the Ligand Profiler module of DS 4.5. In principle, each alkaloid that mapped to a chemical feature of the respective pharmacophore model was counted as one hit. The screening was conducted using the default settings and with a minimal inter-feature distance of 0.5 Å.

### Binding mode refinement

All the poses of the ligands mapped to the pharmacophore were preserved. A series of target–ligand pairs were selected for further examination. The selection was based on compatibility with previously reported pharmacological activities and the traditional use of maca. Further refinement was carried in Molecular Operating Environment (MOE; Chemical Computing Group Inc., Canada) to identify the protein–ligand binding modes. Energy minimization was performed by conjugated gradient minimization with the Merck Molecular Force Field 94× (MMFF94×) until a root-mean-square deviation of 0.1 kcal mol^−1^ Ǻ^−1^ was reached.

### WEGA validation

The WEGA is an accurate shape-based virtual screening method [36]. In this research, we validated the reliability of the binding model by calculating the binding efficiencies of the compounds and the original ligands of the hit targets using the shape similarity calculations function of WEGA.

The sc-PDB also provides separate MOL2 files for the ligand, its binding site, and the corresponding protein chain(s). Ions and cofactors at the vicinity of the ligand are included in the protein. This helps to evaluate the influence of ligand binding on binding site diversity for docking. MOL2 files of the hit-target protein ligands were selected to create the target-ligands database (tl-DB). WEGA validation was performed by comparing the contents of the maca-DB to those of the tl-DB.

### Network construction

The Table 3 showing interactions between all mapped compounds and hit targets shows the ligand profiling results. For each target, the protein name, gene name, and pathway information were collected from the PDB, Kyoto Encyclopedia of Genes and Genomes (KEGG) [37], and Cell Signaling Technology (CST) [38]. The target–target interactions were mapped using GeneMANIA [39]; all targets were analyzed using Ingenuity Pathway Analysis (IPA^®^; Qiagen, Redwood City, CA, USA). All diseases related to the targets were retrieved from the Therapeutic Target Database (TTD) [40] and DrugBank database [41]. The overall compound–target–pathway networks were generated using Cytoscape, version 3.2 (Cytoscape Consortium, USA). In the graphical networks, nodes represent the compounds, targets, and related diseases. The edges linking the compound-target and target-diseases represent their relationships and are marked with two types of lines. The related diseases are marked with different colors at the nodes. The targeted diseases pathway was mapped using the KEGGscape plugin of Cytoscape, version 3.2.

**Table 3:** Four categories of disease targets of the selected compounds from profiling

Target	No. of hit compound	Drugs^a^
*Osteoporosis*		
ABL1	2	Nilotinib, saracatinib, regorafenib
ER-α	4	17-α-ethinylestradiol, fulvestrant, β-estradiol
CSF1R	2	Nilotinib, sunitinib, pazopanib
MMP3	1	Marimastat
C-src	2	Dasatinib, AZM-475271, saracatinib,
MMP9	1	
MMP13	3	Marimastat
CDK9	1	BMS-387032, alvocidib
*Prostate cancer*		
Hsp90-α	15	Alvespimycin, retaspimycin, luminespib
MMP3	1	Marimastat
MET	1	Crizotinib, tivantinib, cabozantinib
AR	3	Testosterone enanthate, enzalutamide, 1-testosterone
MMP9	1	
RXR-α	4	Etretinate, tretinoin, bexarotene
MMP12	4	Marimastat
MMP13	3	Marimastat
MAP2K1	3	Selumetinib, trametinib, dabrafenib
*Kidney diseases*		
MMP9	1	
CA2	11	Ethoxyzolamide, dichlorphenamide, brinzolamide
P450scc	22	
MET	1	Crizotinib, tivantinib, cabozantinib
MIF	1	
sEH	2	
PPAR-γ	20	Icosapent, amlodipine/telmisartan, aleglitazar,
MMP12	4	Marimastat
KIF11	23	
MAPK14	19	Talmapimod, RO-3201195
CA9	1	Girentuximab, methazolamide, hydrochlorothiazide
*Cardiovascular diseases*		
JAK2	19	Tofacitinib, ruxolitinib
F2	8	Enoxaparin, desirudin, dabigatran etexilate
F10	15	Dalteparin, heparin, enoxaparin
REN	1	Aliskiren, aliskiren/valsartan, aliskiren/amlodipine
CA1	1	Ethoxyzolamide, dichlorphenamide, brinzolamide
ER-α	4	17-α-ethinylestradiol, fulvestrant, β-estradiol
MMP3	1	Marimastat
LTA4H	3	
THR-β	1	Amiodarone, levothyroxine, dextrothyroxine,
FGFR1	16	Pazopanib, nintedanib, regorafenib
PLA2G2A	7	Varespladib methyl, varespladib, indomethacin
FLT1	3	Sunitinib, pazopanib, axitinib
FGFR2	2	Nintedanib, regorafenib, dexamethasone/thalidomide
CDK2	26	BMS-387032, alvocidib
EPHX2	2	
KDR	8	Sunitinib, cediranib, pazopanib
PPAR-γ	20	Icosapent, amlodipine/telmisartan, aleglitazar,
MMP12	4	Marimastat
MMP13	3	Marimastat
PIK3CG	12	Dactolisib, buparlisib,
GSK3-β	2	Enzastaurin
CDK9	1	BMS-387032, alvocidib
MAPK10	7	
NR1H2	4	
DHODH	7	Teriflunomide, leflunomide
PPAR-δ	8	Treprostinil, icosapent, bezafibrate
PPAR-α	2	Choline fenofibrate, aleglitazar, gemfibrozil
MAPK14	19	Talmapimod, RO-3201195
NR1H4	1	

^a^All drug information was obtained from IPA analysis and Drugbank

## Results and discussion

### Evaluation of constituents

**Fig. 1:**
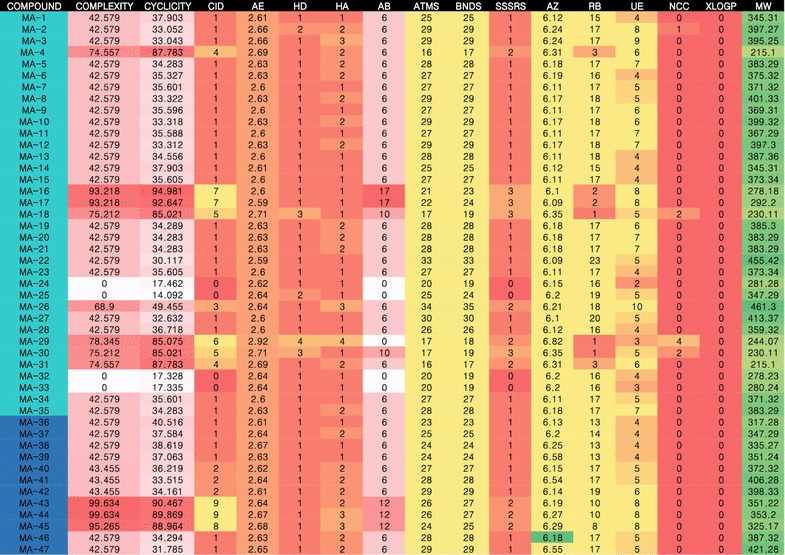
The diversity of compounds analyzed by the scaffold-based classification approach (SCA). CID means compound class ID, categories by complexity; the SCA also outputs the following structural descriptor values: (1) *Cyclicity* side chain value; (2) *AE* average electronegativity; (3) *HD* number of H-bond donors; (3) *HA* number of H-bond acceptors; (4) *AB* number of aromatic bonds; (5) *ATMS* number of non-H atoms; (6) *BNDS* number of non-H-involved bonds; (7) *SSSRS* number of the smallest set of smallest rings; (8) *AZ* average atomic numbers; (9) *RB* number of rotating bonds; (10) *MW* molecular weight

**Fig. 2:**
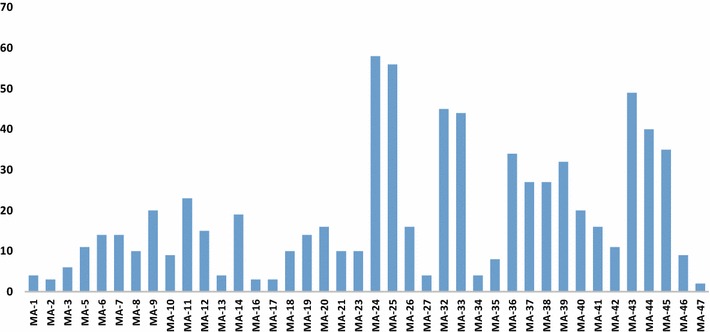
Overview of number of hit compounds of the parallel pharmacophore screening against druggable targets in the field of diseases

In modern drug discovery, large compound libraries are compared, and the diversity of these libraries must be analyzed [42]. The constituents collected and synthesized from maca could be divided into eight compound classes (Fig. 1). The 40 compounds examined in this research were fished by targets (Fig. 2). The compounds with higher degree values were distributed across different categories, such as amide alkaloids (MA-24; 25), macaenes (MA-32; 33), and synthetic amides (MA-43; 44). Compounds that participate in more interactions than other components have a higher bioactivity value.

### Network analysis

In total, 950 models were selected for in silico screening of the maca-DB. These models belong to 125 protein targets; 87 of those targets were validated by the TTD database, were involved in 60 pathways, and were targets of 41 maca constituents. As shown in Fig. 2, we chose three major disease areas (prostate cancer, osteoporosis, and kidney diseases) to validate the traditional medical action and the fished maca compounds.

### Interpreting the mechanisms of action

**Fig. 3:**
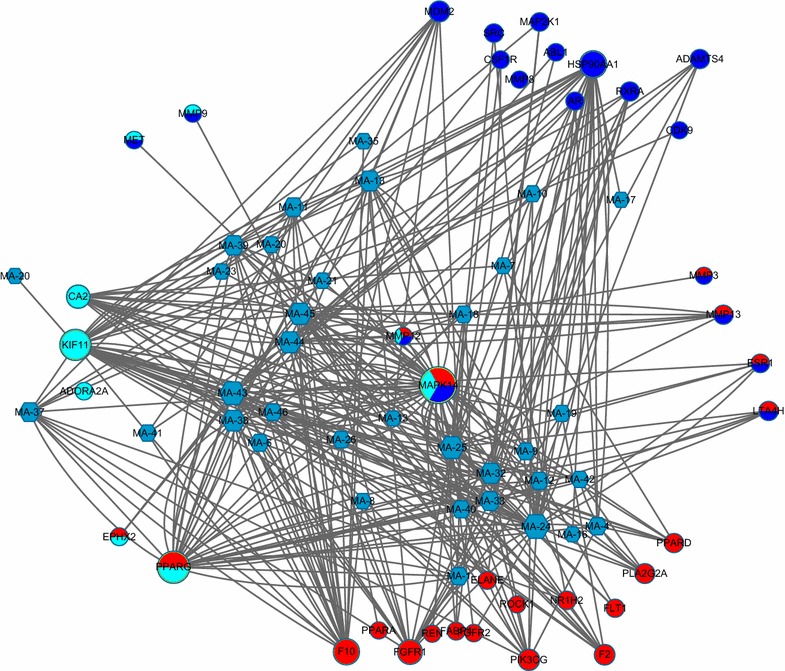
The major pharmacologic network of maca. Hexagon, compounds; circle, targets (*red*: osteoporosis; *light blue*: kidney diseases; *deep blue*: prostate cancer)

An array of well-defined in-house structure and ligand-based pharmacophore models was selected from PharmaDB. For the profiling results, all biological functions of hit targets were annotated from TTD and DrugBank. The identified targets had variable pharmacologic usages, such as the treatment of osteoporosis (8 targets), prostate disease (9 targets), and kidney disease (11 targets). Some targets were related to cardiovascular diseases (CVD), such as hypertension, myocardial infarction, ischemic heart disease, and dyslipidemia (29 targets). Figure 3 and Table 3 provides an overview of the selected targets in the four categories of disease mentioned above. A total of 125 targets were mapped, and the IPA analysis indicated that 107 of them have been used to make drugs. The druggable list is presented in Additional file 1.

### WEGA validation

**Fig. 4:**
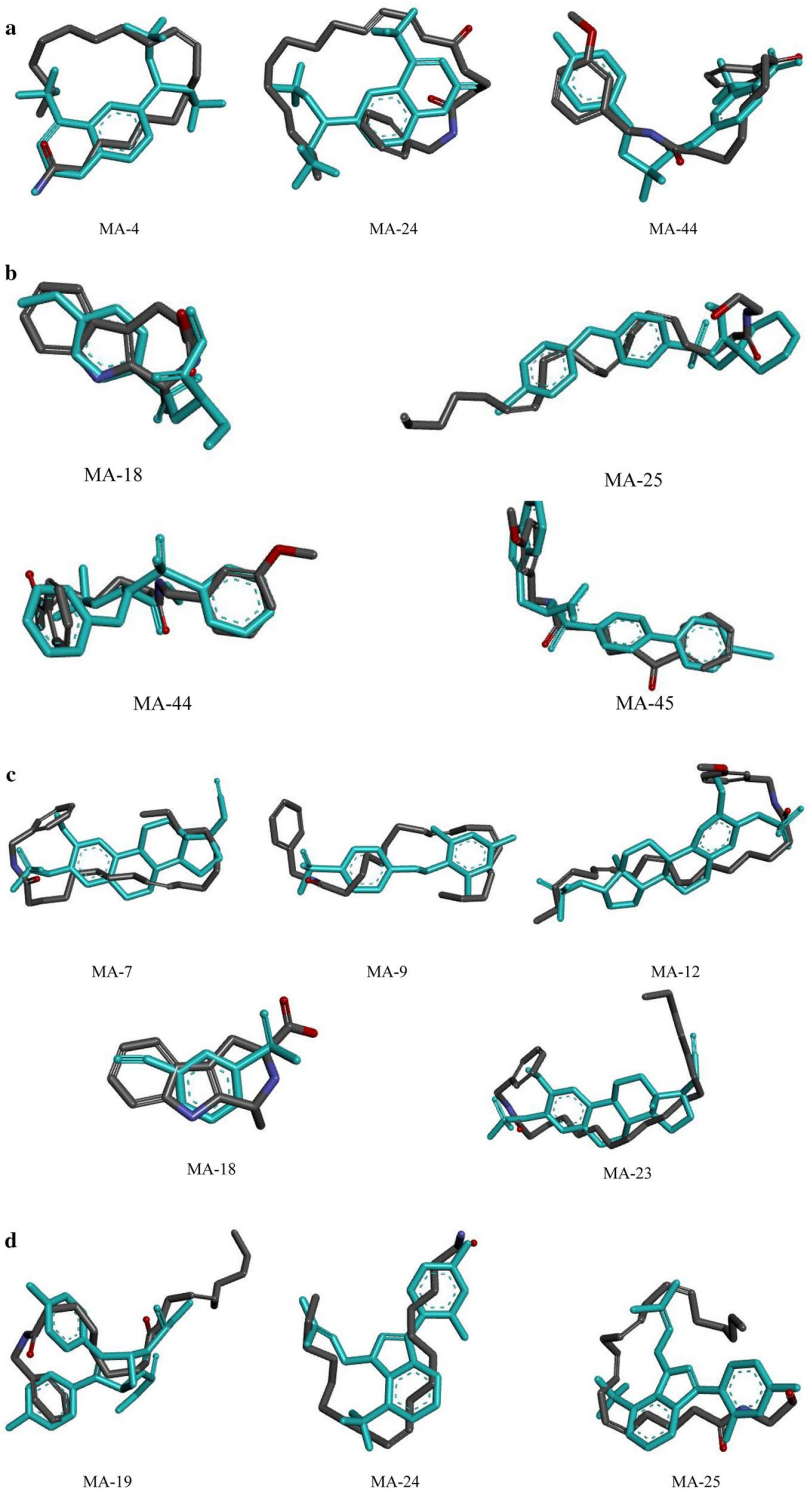
Compounds from maca align with natural ligands from PDB structure (*light blue*) by WEGA (**a** matrix metalloproteinases; **b** androgen receptor; **c** carbonic anhydrase II; **d** estrogen receptor)

The WEGA is suitable for large-scale parallel screening of a series of bioactive compounds; regardless of the conformations of the compounds, their targets can be experimentally determined. The results determined by shape showed that all binding models obtained with DS 4.5 had a high ligand-receptor structure binding value: all scores were above 0.5 (Additional file 2). Important molecular superimposition images are shown in Fig. 4.

### Selected targets related to prostate cancer

**Fig. 5:**
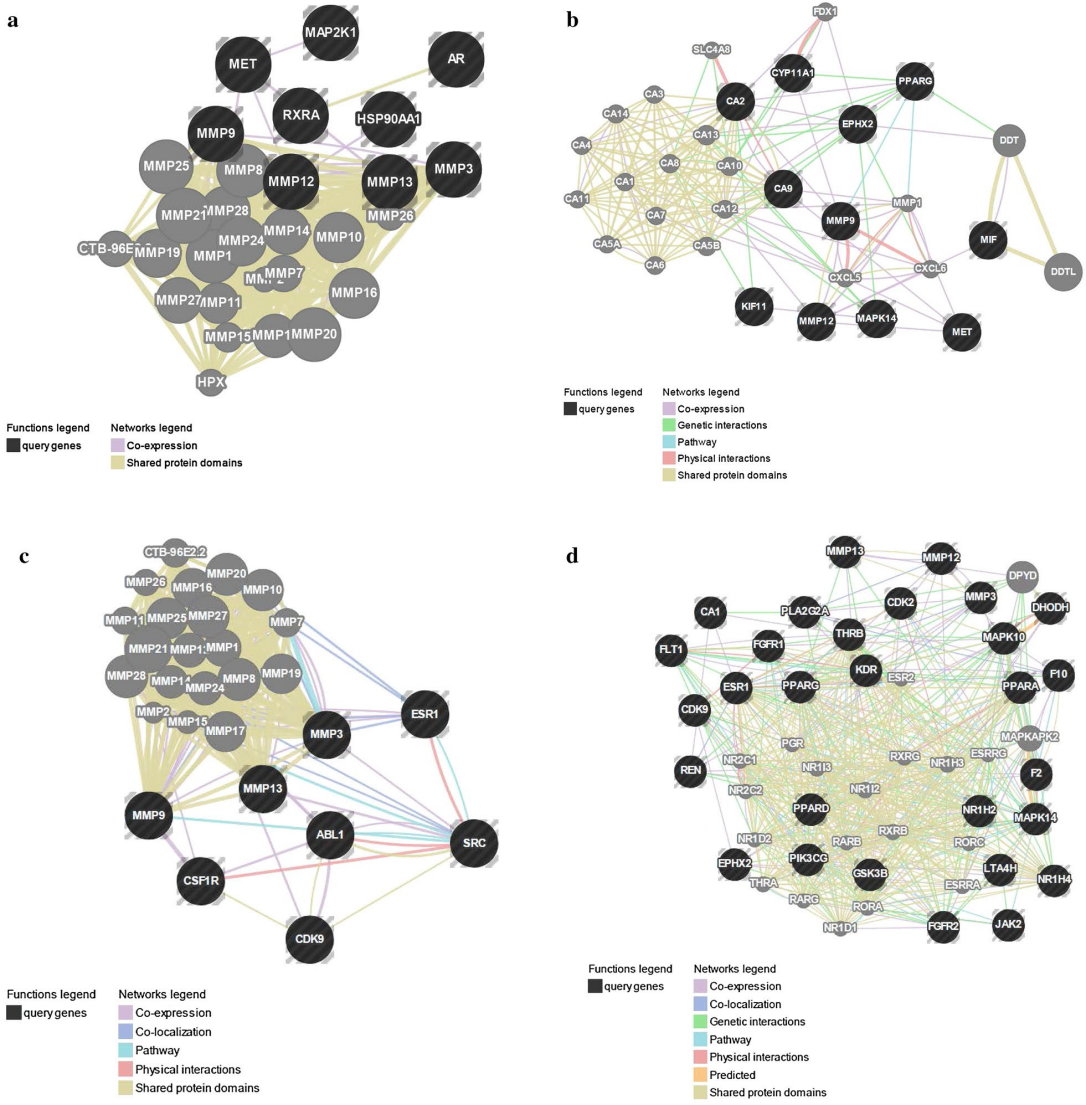
Network images for the interaction of targets related to four disease categories fished by profiling (**a** prostate cancer; **b** kidney diseases; **c** osteoporosis; **d** cardiovascular diseases)

All the gene interactions of these targets were analyzed using GeneMANIA (Fig. 5; Additional file 3). Epidemiological studies have found that consumption of maca could reduce the risk of prostate cancer, which might be associated with aromatic glucosinolate content [7, 8]. Animal experiments in mice [43] and rats [7, 44, 45] showed that maca reduced TE level in a dose-dependent manner and induced prostatic hyperplasia. Red maca aqueous extracts can also reduce ventral prostate size in normal and TE-treated rats [7].

In this study, nine targets annotated by the TTD database were related to prostate cancer. One of the most common targets used in the treatment of prostate diseases is the androgen receptor (AR). In some cell types, testosterone is converted by 5-alpha-reductase into dihydrotestosterone, which is an even more potent agonist for AR activation than testosterone [46]. AR is a sequence-specific DNA-binding protein involved in cellular proliferation in prostate cancer and in the development of secondary sexual characteristics through activation by dihydrotestosterone [47]. AR is also involved in the regulation of the adhesion of prostate cancer cells to the extracellular matrix and to the invasion of prostate cancer cells through its influence on the expression of specific integrin subunits [48]. There is increasing evidence that the genus *Lepidium* could reduce the risk of prostate cancer development [49, 50]. Research also suggests that other cruciferous plants from the genus *Lepidium* could be used as important alternative treatments for prostate diseases [51]. Growth of the prostate is a hormone-mediated phenomenon regulated by both androgens and estrogens [52]. A recent report indicates that maca’s effect on ventral prostate size may partly be a result of the action of glucosinolate metabolites on AR [28]. In this study, AR was fished by MA-4, MA-24, and MA-44 (Fig. 5a).

Stromelysin-1, also known as matrix metalloproteinase-3 (MMP-3), is an enzyme that activates other targeted matrix metalloproteinases (MMPs): MMP-9, MMP-12, and MMP-13 [53]. The expression of MMPs is primarily regulated at the level of transcription, where the promoter of the gene responds to various stimuli, including growth factors, cytokines, tumor promoters, and oncogene products [54]. MMPs are associated with various physiological and pathological processes, such as morphogenesis, angiogenesis, tissue repair, cirrhosis, arthritis, and metastasis [55]. MMPs also play a significant role in the development and metastasis of prostate cancer [56]. In particular, MMP-9 has been shown experimentally to be involved in prostate cancer [57]. These four targets share similar protein domains and were fished by MA-18, MA-25, MA-44, and MA-45 (Fig. 5b).

Proto-oncogene tyrosine-protein kinase Src (c-Src) is a non-receptor tyrosine kinase protein that may be involved in cancer progression by promoting other signals [58]. C-Src is highly expressed in malignant prostate cells [59]. When primary prostate cells were treated with a c-Src inhibitor in vitro, their proliferation, migration, and invasive potential were reduced [60].

As c-Src is a solid druggable target, several c-Src tyrosine kinase inhibitors have been utilized therapeutically [61]. Dasatinib has been approved for the treatment of chronic myeloid leukemia [62], which is an Src family inhibitor. Based on the binding results of those compounds that fished c-Src, we can predict that MA-24 and MA-25 may be pharmacologically similar to Dasatinib.

### Selected targets related to kidney diseases

Renal tubular acidosis (RTA) is a metabolic acidosis caused by impaired excretion of acid by the kidney. Carbonic anhydrase II (CA2) is one of the 14 forms of human α carbonic anhydrases and the one with the highest catalytic activity. The physiological functions of CA2 include pH regulation, CO_2_ and H_2_CO_3_ transport, and maintenance of H_2_O and electrolyte balance. CA2 deficiency syndrome can lead to osteoporosis, RTA, and cerebral calcification.

In inherited CA2 deficiency, isolated proximal RTA presents with osteoporosis (owing to impaired osteoclast function), cerebral calcification, and variable levels of mental retardation. Although this form of inherited RTA is clinically more proximal, it can also present with a mixed proximal and distal phenotype, which reflects the presence of CA2 in cells all along the renal tubule. CA2 was fished by MA-18, MA-24, MA-38, MA-44, and MA-45 (Fig. 5c). Kidney status directly affects the reproductive function, especially sexual behavior. Oral administration of a purified lipidic extract from maca could enhance sexual behavior by increasing the number of complete intromissions in normal mice and decreasing the latent period of erection in erectile dysfunction male rats [63].

### Selected targets related to osteoporosis

Osteoporosis is a skeletal fragility disorder and is common in elderly people. Its prevalence is increasing as more individuals are developing low bone mineral density [64]. The edible part of maca, the hypocotyl, has been widely used to treat osteoporosis [65]. Ethanol extract of maca has anti-osteoporotic activity and indicated maca alkaloids, steroids, glucosinolates, isothiocyanates, and macamides are probably responsible for its biological functions [19].

Estrogen receptor α (ER-α) binds to estrogens and regulates bone homeostasis and prevents postmenopausal bone loss [66, 67]. Estrogen deficiency is a major determinant of bone loss in postmenopausal women [68, 69]. In one ovariectomized rats experiment, ER-α was the predominant ER form expressed in mesenchymal stem cells [70]. Co-expression of ER-α with other genes indicates its activator function in the osteogenic differentiation of mesenchymal stem cells, which causes osteoporosis [71]. Estradiol, estrone, and raloxifene bind to the alpha receptor. However, because the ER’s helix 12 domain plays a crucial role in determining its interactions with co-activators and co-repressors, different ER combinations may respond differently to various ligands, which may translate into tissue selective agonistic and antagonistic effects [72, 73]. For example, tamoxifen is an antagonist in the breast and is used as a breast cancer treatment, but it is an ER agonist in bone and therefore prevents osteoporosis [69]. Recent studies have suggested that maca contains phyto-estrogens, which may have estrogenic activity [74, 75]. We found that three compounds were connected to ER: MA-19, MA-24, and MA-25 (Fig. 5d).

Enzymatic cleavage by MMPs is involved in the destruction of articular cartilage, and the high expression of MMP-9 and MMP-13 could be detected in pathologic synovium and cartilage samples [76]. Several natural substances containing maca extract tested in vitro are effective agents, as evidenced by the strong regulation of MMP-9 and MMP-13 [77]. In osteoclast migration, MMPs control the cell–matrix interactions required in the model of osteoclast recruitment in primitive long bones [78]. As classical anti-osteoporotic agents, bisphosphonates are involved in the inhibition of the functions of several MMPs (MMP-3, -9, -12, and -13), which were mapped in this virtual screening.

### Prediction of the function of maca compounds in the treatment of cardiovascular diseases

A total of 29 targets related to CVD were mapped. Maca could be used in the treatment of CVD characterized by atherogenic lipoprotein profile, and showed relevant angiotensin I-converting enzyme inhibitory activities, indicating potential anti-hypertension activity; however, the mechanisms of these activities are still to be clarified. This result indicated that maca might have significant potential for the treatment of CVD.

Kinase activity mediated by mitogen-activated protein kinase 14 (MAPK14), also called p38α, has been identified in many tissues [79]. p38α is mainly activated through MAPK kinase kinase cascades and exerts its biological function via downstream substrate phosphorylation [80]. Pharmacological and genetic inhibition of p38α has revealed its biological significance regarding physiological functions and its potential for targeting p38α in human diseases, especially CVD [81–83]. MAPK14 activity regulates myocyte cytokinesis and promotes cell-cycle exit during maturation in the newborn mouse heart [84]. MAPK14 has also been associated with cell-cycle arrest in mammalian cardiomyocytes [85], and its inhibition might be a strategy to promote cardiac regeneration in response to injury [86]. Furthermore, MAPK14 promoted myocyte apoptosis and cardiomyocyte hypertrophy, and targeted IRS-1-mediated Akt signaling and promoted myocyte death under chronic insulin stimulation in vitro [87, 88].

Peroxisome proliferator-activated receptors (PPARs) are a group of nuclear receptor proteins [89], including PPARα, PPARδ, and PPARγ, whose ligand and DNA-binding domains share 60–80 % homology [90, 91]. PPARs are widely expressed in the vasculature, myocardium, and the immune cells, such as monocytes and macrophages [92]. Additionally, PPAR-retinoid X receptor heterodimers repress CLOCK/BMAL1 gene expression [93]. Hence, PPARs could regulate the expression of a series of genes involved in metabolism that impact cardiovascular physiology [94]. Different PPAR isoforms are observed in various cardiovascular pathologies, such as atherosclerosis, hypertension, and cardiac hypertrophy [95]. Both PPARα and PPARγ are expressed in endothelial cells, vascular smooth muscle cells, and monocytes/macrophages [96, 97]. In atherosclerosis, activation of these two proteins reduces leukocyte recruitment and cell adhesion [98]. Both regulate cytokine-induced genes (such as VCAM-1 and tissue factor), and PPARα and PPARγ inhibit the expressions of tumor necrosis factor-α and MCP-1, respectively [99]. PPARδ activation decreases the expressions of MCP-1, ICAM-1, and inflammatory cytokines and attenuates atherosclerosis development [100].

The potential use of PPAR agonists and dual PPAR agonists, including PPARα/γ, PPARα/δ, and PPARδ/γ dual agonists, in the treatment of CVD has recently received attention [101]. Compounds that are capable of targeting more than one PPAR isotype and are effective at treating CVD have emerged as an interesting and efficient treatment approach. Both MAPK14 and PPARs are related to a series of maca compounds (Table 4).

**Table 4:** Compounds fished by MAPK14 and PPARs

Targets related to CVD	Compounds
MAPK14	MA-1, 6, 9, 12, 21, 23, 24, 25, 36, 37, 38, 39, 40, 43
PPARα	MA-26
PPARγ	MA-1, 18, 24, 36
PPARδ	MA-4

## Conclusion

In silico target fishing identified maca’s traditional effects on treatment and prevention of osteoporosis, prostate cancer, and kidney diseases, and its potential function of treating cardiovascular diseases, as the most important of this herb’s possible activities.

## Additional files

10.1186/s13020-016-0112-y IPA druggable datasheet.
10.1186/s13020-016-0112-y WEGA validation scores.
10.1186/s13020-016-0112-y Total GeneMANIA report of maca.

## Abbreviations

AR: androgen receptor
BMD: bone mineral density
CA2: carbonic anhydrase II
CHARMM: chemistry at Harvard macromolecular mechanics
c-Src: tyrosine-protein kinase Src
CST: cell signaling technology
CVD: cardiovascular disease
DS: discovery studio
ER-α: estrogen receptor α
IPA: ingenuity pathway analysis
KEGG: Kyoto encyclopedia of genes and genomes
LPE: latent period of erection
MAPK14: mitogen-activated protein kinase 14
MMFF94x: Merck molecular force field 94×
MMPs: matrix metalloproteinases
MOE: molecular operating environment
MSCs: mesenchymal stem cells
PDB: protein data bank
PPARs: peroxisome proliferator-activated receptors
RMSD: root mean square deviation
RTA: renal tubular acidosis
TE: testosterone enanthate
TTD: therapeutic target database
WEGA: weighted Gaussian algorithm

## References

[1] Hermann M, Heller J (1997). Andean roots and tubers: ahipa, arracacha, maca and yacon.

[2] Esparza E, Hadzich A, Kofer W, Mithöfer A, Cosio EG (2015). Bioactive maca (*Lepidium meyenii*) alkamides are a result of traditional Andean postharvest drying practices. Phytochemistry.

[3] León J (1964). The, “Maca” (Lepidium meyenii), A little known food plant of Peru. Econ Bot.

[4] Gonzales GF, Singh VK, Govil JN, Ahmad K, Sharma RK (2007). Biological effects of *Lepidium meyenii*, maca, a plant from the highlands of Peru. Nat Prod.

[5] Sandoval M, Okuhama NN, Angeles FM, Melchor VV, Condezo LA, Lao J, Miller MJS (2002). Antioxidant activity of the cruciferous vegetable Maca (*Lepidium meyenii*). Food Chem.

[6] Piacente S, Carbone V, Plaza A, Zampelli A, Pizza C (2002). Investigation of the tuber constituents of maca (*Lepidium meyenii* Walp.). J Agric Food Chem.

[7] Gonzales GF, Miranda S, Nieto J, Fernández G, Yucra S, Rubio J, Yi P, Gasco M (2005). Red maca (*Lepidium meyenii*) reduced prostate size in rats. Reprod Biol Endocrinol..

[8] Večeřa R, Orolin J, Škottová N, Kazdová L, Oliyarnik O, Ulrichová J, Šimánek V (2007). The influence of maca (*Lepidium meyenii*) on antioxidant status, lipid and glucose metabolism in rat. Plant Food Hum Nutr..

[9] Wu H, Kelley CJ, Pino-Figueroa A, Vu HD, Maher TJ (2013). Macamides and their synthetic analogs: evaluation of in vitro FAAH inhibition. Bioorg Med Chem..

[10] Cicero AFG, Bandieri E, Arletti R (2001). *Lepidium meyenii* Walp. improves sexual behaviour in male rats independently from its action on spontaneous locomotor activity. J Ethnopharmacol.

[11] Ruiz-Luna AC, Salazar S, Aspajo NJ, Rubio J, Gasco M, Gonzales GF (2005). *Lepidium meyenii* (Maca) increases litter size in normal adult female mice. Reprod Biol Endocrinol..

[12] Lee KJ, Dabrowski K, Rinchard J, Gomez C, Guz L, Vilchez C (2004). Supplementation of maca (*Lepidium meyenii*) tuber meal in diets improves growth rate and survival of rainbow trout *Oncorhynchus mykiss* (Walbaum) alevins and juveniles. Aquac Res.

[13] Clément C, Kneubühler J, Urwyler A, Witschi U, Kreuzer M (2010). Effect of maca supplementation on bovine sperm quantity and quality followed over two spermatogenic cycles. Theriogenology.

[14] Gonzales C, Rubio J, Gasco M, Nieto J, Yucra S, Gonzales GF (2006). Effect of short-term and long-term treatments with three ecotypes of *Lepidium meyenii* (MACA) on spermatogenesis in rats. J Ethnopharmacol.

[15] Gasco M, Villegas L, Yucra S, Rubio J, Gonzales GF (2007). Dose-response effect of Red Maca (*Lepidium meyenii*) on benign prostatic hyperplasia induced by testosterone enanthate. Phytomedicine.

[16] Lembè DM, Gasco M, Gonzales GF (2012). Fertility and estrogenic activity of Turraeanthus africanus in combination with *Lepidium meyenii* (Black maca) in female mice. Eur J Integr Med..

[17] Rubio J, Qiong W, Liu XM, Jiang Z, Dang HX, Chen SL, Gonzales GF (2011). Aqueous extract of black maca (*Lepidium meyenii*) on memory impairment induced by ovariectomy in mice. Evid Based Complement Alternat Med..

[18] Rubio J, Dang H, Gong M, Liu XM, Chen SL, Gonzales GF (2007). Aqueous and hydroalcoholic extracts of Black Maca (*Lepidium meyenii*) improve scopolamine-induced memory impairment in mice. Food Chem Toxicol.

[19] Rosales-Hartshorn M (2015). Maca: botanical medicine from the Andes. Adv Food Tech Nutr Sci Open J..

[20] Lentz A, Gravitt K, Carson CC, Marson L (2007). Acute and chronic dosing of *Lepidium meyenii* (Maca) on male rat sexual behavior. J Sex Med..

[21] Shin BC, Lee MS, Yang EJ, Lim HS, Ernst E (2010). Maca (*L. meyenii*) for improving sexual function: a systematic review. BMC Complement Altern Med..

[22] Dror O, Shulman-Peleg A, Nussinov R, Wolfson HJ (2004). Predicting molecular interactions in silico: I. A guide to pharmacophore identification and its applications to drug design. Curr Med Chem.

[23] Taha MO, Bustanji Y, Al-Bakri AG, Yousef AM, Zalloum WA, Al-Masri IM, Atallah N (2007). Discovery of new potent human protein tyrosine phosphatase inhibitors via pharmacophore and QSAR analysis followed by in silico screening. J Mol Graph Model.

[24] Muhammad I, Zhao JP, Dunbar DC, Khan IA (2002). Constituents of *Lepidium meyenii* ‘maca’. Phytochemistry.

[25] Cui BL, Zheng BL, He K, Zheng QY (2003). Imidazole alkaloids from *Lepidium meyenii*. J Nat Prod.

[26] Zhao JP, Muhammad I, Dunbar DC, Mustafa J, Khan IA (2005). New alkamides from maca (*Lepidium meyenii*). J Agric Food Chem.

[27] McCollom MM, Villinski JR, McPhail KL, Craker LE, Gafner S (2005). Analysis of macamides in samples of Maca (*Lepidium meyenii*) by HPLC-UV-MS/MS. Phytochem Anal.

[28] Tellez MR, Khan IA, Kobaisy M, Schrader KK, Dayan FE, Osbrink W (2002). Composition of the essential oil of *Lepidium meyenii* (Walp.). Phytochemistry.

[29] Gonzales GF, Gonzales-Castañeda C (2009). The methyltetrahydro-β-carbolines in Maca (*Lepidium meyenii*). Evid Based Complement Alternat Med..

[30] Ganzera M, Zhao J, Muhammad I, Khan IA (2002). Chemical profiling and standardization of *Lepidium meyenii* (Maca) by reversed phase high performance liquid chromatography. Chem Pharm Bull.

[31] Gonzales GF (2012). Ethnobiology and ethnopharmacology of *Lepidium meyenii* (Maca), a plant from the Peruvian highlands. Evid Based Complement Alternat Med.

[32] O’Boyle NM, Banck M, James CA, Morley C, Vandermeersch T, Hutchison GR (2011). Open babel: an open chemical toolbox. J Cheminform.

[33] Guner OF (2000). Pharmacophore perception, development, and use in drug design,.

[34] Giannakakou P, Gussio R, Nogales E, Downing KH, Zaharevitz D, Bollbuck B, Poy G, Sackett D, Nicolaou K, Fojo T (2000). A common pharmacophore for epothilone and taxanes: molecular basis for drug resistance conferred by tubulin mutations in human cancer cells. Proc Natl Acad Sci USA.

[35] http://www.rcsb.org/pdb/home/home.do.

[36] Yan X, Li J, Liu Z, Zheng M, Ge H, Xu J (2013). Enhancing molecular shape comparison by weighted Gaussian functions. J Chem Inf Model.

[37] http://www.kegg.jp.

[38] http://www.cellsignal.com/contents/science/cst-pathways/science-pathways.

[39] Warde-Farley D, Donaldson SL, Comes O, Zuberi K, Badrawi R, Chao P, Franz M, Grouios C, Kazi F, Lopes CT, Maitland A, Mostafavi S, Montojo J, Shao Q, Wright G, Bader GD, Morris Q (2010). The GeneMANIA prediction server: biological network integration for gene prioritization and predicting gene function. Nucleic Acids Res.

[40] Zhu F, Shi Z, Qin C, Tao L, Liu X, Xu F, Zhang L, Song Y, Liu XH, Zhang JX, Han BC, Zhang P, Chen YZ (2012). Therapeutic target database update 2012: a resource for facilitating target-oriented drug discovery. Nucleic Acids Res.

[41] Knox C, Law V, Jewison T, Liu P, Ly S, Frolkis A, Pon A, Banco K, Mak C, Neveu V, Djoumbou Y, Eisner R, Guo AC, Wishart DS (2011). DrugBank 3.0: a comprehensive resource for ‘omics’ research on drugs. Nucleic Acids Res.

[42] Xu J (2002). A new approach to finding natural chemical structure classes. J Med Chem.

[43] Gonzales GF, Gasco M, Malheiros-Pereira A, Gonzales-Castañeda C (2008). Antagonistic effect of *Lepidium meyenii* (red maca) on prostatic hyperplasia in adult mice. Andrologia..

[44] Gonzales GF, Vasquez V, Rodriguez D, Maldonado C, Mormontoy J, Portella J, Pajuelo M, Villegas L, Gasco M, Mathur P (2007). Effect of two different extracts of red maca in male rats with testosterone-induced prostatic hyperplasia. Asian J Androl..

[45] Gasco M, Villegas L, Yucra S, Rubio J, Gonzales G (2007). Dose-response effect of Red Maca (*Lepidium meyenii*) on benign prostatic hyperplasia induced by testosterone enanthate. Phytomedicine.

[46] Davis SR, Worsley R (2014). Androgen treatment of postmenopausal women. J Steroid Biochem..

[47] Fu M, Wang CG, Reutens AT, Wang J, Angeletti RH, Siconolfi-Baez L, Ogryzko V, Avantaggiati ML, Pestell RG (2000). p300 and p300/cAMP-response element-binding protein-associated factor acetylate the androgen receptor at sites governing hormone-dependent transactivation. J Biol Chem.

[48] Nagakawa O, Akashi T, Hayakawa Y, Junicho A, Koizumi K, Fujiuchi Y, Furuya Y, Matsuda T, Fuse H, Saiki I (2004). Differential expression of integrin subunits in DU-145/AR prostate cancer cells. Oncol Rep.

[49] Azimi H, Khakshur AA, Aghdasi I, Fallah-Tafti M, Abdollahi M (2012). A review of animal and human studies for management of benign prostatic hyperplasia with natural products: perspective of new pharmacological agents. Inflamm Allergy Drug Targets.

[50] Shrivastava A, Gupta VB (2012). Various treatment options for benign prostatic hyperplasia: a current update. J Midlife Health..

[51] Marti S, Fernandez CC, Perez-Fernandez R (2004). Effect of an integral suspension of *Lepidium latifolium* on prostate hyperplasia in rats. Fitoterapia.

[52] Lund TD, Munson DJ, Adlercreutz H, Handa RJ, Lephart ED (2004). Androgen receptor expression in the rat prostate is down-regulated by dietary phytoestrogens. Reprod Biol Endocrinol..

[53] Egeblad M, Werb Z (2002). New functions for the matrix metalloproteinases in cancer progression. Nat Rev Cancer.

[54] Matrisian LM (1990). Metalloproteinases and their inhibitors in matrix remodeling. Trends Genet.

[55] Eguchi T, Kubota S, Kawata K, Mukudai Y, Uehara J, Ohgawara T, Ibaragi S, Sasaki A, Kuboki T, Takigawa M (2008). Novel transcription factor-like function of human matrix metalloproteinase 3 regulating the *CTGF*/*CCN2* gene. Mol Cell Biol.

[56] Lokeshwar BL (1999). MMP inhibition in prostate cancer. Ann NY Acad Sci..

[57] Kong DJ, Li YW, Wang ZW, Banerjee S, Sarkar FH (2007). Inhibition of angiogenesis and invasion by 3,3′-diindolylmethane is mediated by the nuclear factor-κB downstream target genes *MMP*-*9* and *uPA* that regulated bioavailability of vascular endothelial growth factor in prostate cancer. Cancer Res.

[58] Devary Y, Gottlieb RA, Smeal T, Karin M (1992). The mammalian ultraviolet response is triggered by activation of Src tyrosine kinases. Cell.

[59] Wheeler DL, Iida M, Dunn EF (2009). The role of Src in solid tumors. Oncologist..

[60] Park SI, Zhang J, Phillips KA, Araujo JC, Najjar AM, Volgin AY, Gelovani JG, Kim SJ, Wang Z, Gallick GE (2008). Targeting Src family kinases inhibits growth and lymph node metastases of prostate cancer in an orthotopic nude mouse model. Cancer Res.

[61] Musumeci F, Schenone S, Brullo C, Botta M (2012). An update on dual Src/Abl inhibitors. Future Med Chem..

[62] Breccia M, Salaroli A, Molica M, Alimena G (2013). Systematic review of dasatinib in chronic myeloid leukemia. Onco Targets Ther..

[63] Zheng BL, He K, Kim CH, Rogers LL, Shao Y, Huang ZY, Lu Y, Yan SJ, Qien LC, Zheng QY (2000). Effect of a lipidic extract from *lepidium meyenii* on sexual behavior in mice and rats. Urology..

[64] Hodgson SF, Watts NB, Bilezikian JP, Clarke BL, Gray TK, Harris DW, Johnston CC, Kleerekoper M, Lindsay R, Luckey MM, McClung MR, Nankin HR, Petak SM, Recker RR (2003). American association of clinical endocrinologists medical guidelines for clinical practice for the prevention and treatment of postmenopausal osteoporosis: 2001 edition, with selected updates for 2003. Endocr Pract..

[65] Zhang YZ, Yu LJ, Ao MZ, Jin WW (2006). Effect of ethanol extract of *Lepidium meyenii* Walp. on osteoporosis in ovariectomized rat. J Ethnopharmacol.

[66] Dahlman-Wright K, Cavailles V, Fuqua SA, Jordan VC, Katzenellenbogen JA, Korach KS, Maggi A, Muramatsu M, Parker MG, Gustafsson JA (2006). International union of pharmacology LXIV. Estrogen receptors. Pharmacol Rev.

[67] Eriksen EF, Colvard DS, Berg NJ, Graham ML, Mann KG, Spelsberg TC, Riggs BL (1988). Evidence of estrogen receptors in normal human osteoblast-like cells. Science.

[68] Sims NA, Dupont S, Krust A, Clement-Lacroix P, Minet D, Resche-Rigon M, Gaillard-Kelly M, Baron R (2002). Deletion of estrogen receptors reveals a regulatory role for estrogen receptors-β in bone remodeling in females but not in males. Bone.

[69] Deroo BJ, Korach KS (2006). Estrogen receptors and human disease. J Clin Invest..

[70] Clark CR, MacLusky NJ, Parsons B, Naftolin F (1981). Effects of estrogen deprivation on brain estrogen and progestin receptor levels and the activation of female sexual behavior. Horm Behav.

[71] Zhou S, Zilberman Y, Wassermann K, Bain SD, Sadovsky Y, Gazit D (2001). Estrogen modulates estrogen receptor α and β expression, osteogenic activity, and apoptosis in mesenchymal stem cells (MSCs) of osteoporotic mice. J Cell Biochem Suppl.

[72] Bakas P, Liapis A, Vlahopoulos S, Giner M, Logotheti S, Creatsas G, Meligova AK, Alexis MN, Zoumpourlis V (2008). Estrogen receptor α and β in uterine fibroids: a basis for altered estrogen responsiveness. Fertil Steril.

[73] Shang Y, Brown M (2002). Molecular determinants for the tissue specificity of SERMs. Science.

[74] Rochira V, Balestrieri A, Madeo B, Baraldi E, Faustini-Fustini M, Granata AR, Carani C (2001). Congenital estrogen deficiency: in search of the estrogen role in human male reproduction. Mol Cell Endocrinol.

[75] Cutillo DM. Method for metabolic correction. US Patent 20,150,313,949. 2015.

[76] Oh H, Yang S, Park M, Chun JS (2008). Matrix metalloproteinase (MMP)-12 regulates MMP-9 expression in interleukin-1β-treated articular chondrocytes. J Cell Biochem.

[77] Akhtar N, Miller MJ, Haqqi TM (2011). Effect of a Herbal-Leucine mix on the IL-1β-induced cartilage degradation and inflammatory gene expression in human chondrocytes. BMC Complement Altern Med..

[78] Delaissé JM, Andersen TL, Engsig MT, Henriksen K, Troen T, Blavier L (2003). Matrix metalloproteinases (MMP) and cathepsin K contribute differently to osteoclastic activities. Microsc Res Tech.

[79] Lee JC, Laydon JT, McDonnell PC, Gallagher TF, Kumar S, Green D, McNulty D, Blumenthal MJ, Heys JR, Landvatter SW (1994). A protein kinase involved in the regulation of inflammatory cytokine biosynthesis. Nature.

[80] Han J, Lee JD, Bibbs L, Ulevitch RJ (1994). A MAP kinase targeted by endotoxin and hyperosmolarity in mammalian cells. Science.

[81] Alexa A (2013). Characterisation of mapk14/mapk11 mediated cardioprotection using RNAi. Circulation.

[82] Waterworth DM, Li L, Scott R, Warren L, Gillson C, Aponte J, Sarov-Blat L, Sprecher D, Dupuis J, Reiner A, Psaty BM, Tracy RP, Lin H, McPherson R, Chissoe S, Wareham N, Ehm MG (2014). A low-frequency variant in MAPK14 provides mechanistic evidence of a link with myeloperoxidase: a prognostic cardiovascular risk marker. J Am Heart Assoc..

[83] Muslin AJ (2008). MAPK signalling in cardiovascular health and disease: molecular mechanisms and therapeutic targets. Clin Sci.

[84] Liu Z, Fan HY, Wang Y, Richards JS (2010). Targeted disruption of *Mapk14* (*p38 MAPK α*) in Granulosa cells and Cumulus cells causes cell-specific changes in gene expression profiles that rescue COC expansion and maintain fertility. Mol Endocrinol.

[85] Wagner EF, Nebreda ÁR (2009). Signal integration by JNK and p38 MAPK pathways in cancer development. Nat Rev Cancer.

[86] Jopling C, Boue S, Izpisua Belmonte JC (2011). Dedifferentiation, transdifferentiation and reprogramming: three routes to regeneration. Nat Rev Mol Cell Biol.

[87] Knowlton DL, Tang K, Henstock PV, Subramanian RR (2011). miRNA alterations modify kinase activation in the IGF-1 pathway and correlate with colorectal cancer stage and progression in patients. J Cancer..

[88] Földes G, Mioulane M, Wright JS, Liu AQ, Novak P, Merkely B, Gorelik J, Schneider MD, Ali NN, Harding SE (2011). Modulation of human embryonic stem cell-derived cardiomyocyte growth: a testbed for studying human cardiac hypertrophy?. J Mol Cell Cardiol.

[89] Michalik L, Auwerx J, Berger JP, Chatterjee VK, Glass CK, Gonzalez FJ, Grimaldi PA, Kadowaki T, Lazar MA, O’Rahilly S, Palmer CN, Plutzy J, Reddy JK, Spiegelman BM, Staels B, Wahli W (2006). International Union of Pharmacology LXI. Peroxisome proliferator-activated receptors. Pharmacol Rev.

[90] Dreyer C, Krey G, Keller H, Givel F, Helftenbein G, Wahli W (1992). Control of the peroxisomal β-oxidation pathway by a novel family of nuclear hormone receptors. Cell.

[91] Ziouzenkova O, Perrey S, Marx N, Bacqueville D, Plutzky J (2002). Peroxisome proliferator-activated receptors. Curr Atheroscler Rep..

[92] Abdelrahman M, Sivarajah A, Thiemermann C (2005). Beneficial effects of PPAR-γ ligands in ischemia-reperfusion injury, inflammation and shock. Cardiovasc Res.

[93] Nakamura K, Inoue I, Takahashi S, Komoda T, Katayama S (2008). Cryptochrome and period proteins are regulated by the CLOCK/BMAL1 Gene: crosstalk between the PPARs/RXRα-regulated and CLOCK/BMAL1-regulated system. PPAR Res..

[94] Chandra V, Huang P, Hamuro Y, Raghuram S, Wang Y, Burris TP, Rastinejad F (2008). Structure of the intact PPAR-γ-RXR-α nuclear receptor complex on DNA. Nature.

[95] Asakawa M, Takano H, Nagai T, Uozumi H, Hasegawa H, Kubota N, Saito T, Masuda Y, Kadowaki T, Komuro I (2002). Peroxisome proliferator-activated receptor γ plays a critical role in inhibition of cardiac hypertrophy in vitro and in vivo. Circulation.

[96] Marx N, Sukhova GK, Collins T, Libby P, Plutzky J (1999). PPARα activators inhibit cytokine-induced vascular cell adhesion molecule-1 expression in human endothelial cells. Circulation.

[97] Marx N, Bourcier T, Sukhova GK, Libby P, Plutzky J (1999). PPARγ activation in human endothelial cells increases plasminogen activator inhibitor type-1 expression PPARγ as a potential mediator in vascular disease. Arterioscler Thromb Vasc Biol.

[98] Kersten S, Desvergne B, Wahli W (2000). Roles of PPARs in health and disease. Nature.

[99] Tham DM, Martin-McNulty B, Wang YX, Wilson DW, Vergona R, Sullivan ME, Dole W, Rutledge JC (2002). Angiotensin II is associated with activation of NF-κB-mediated genes and downregulation of PPARs. Physiol Genomics.

[100] Rival Y, Benéteau N, Taillandier T, Pezet M, Dupont-Passelaigue E, Patoiseau JF, Junquéro D, Colpaert FC, Delhon A (2002). PPARα and PPARδ activators inhibit cytokine-induced nuclear translocation of NF-κB and expression of VCAM-1 in EAhy926 endothelial cells. Eur J Pharmacol.

[101] Henke BR (2004). Peroxisome proliferator-activated receptor α/γ dual agonists for the treatment of type 2 diabetes. J Med Chem.

