# Supplementary material for: In silico profiling for secondary metabolites from Lepidium meyenii (maca) by the pharmacophore and ligand-shape-based joint approach
Source: Chin Med. 2016 Sep 26;11:42. doi: 10.1186/s13020-016-0112-y (PMC5037646; doi:10.1186/s13020-016-0112-y)
Supplement: Supplementary file 3 — 10.1186/s13020-016-0112-y Total GeneMANIA report of maca. [file 13020_2016_112_MOESM3_ESM.pdf]

GeneMANIA

Created on: 23 July 2015 10:25:46
Last database update: 1 June 2014 20:00:00
Application version: 3.1.2.8

Report of GeneMANIA search

Network image

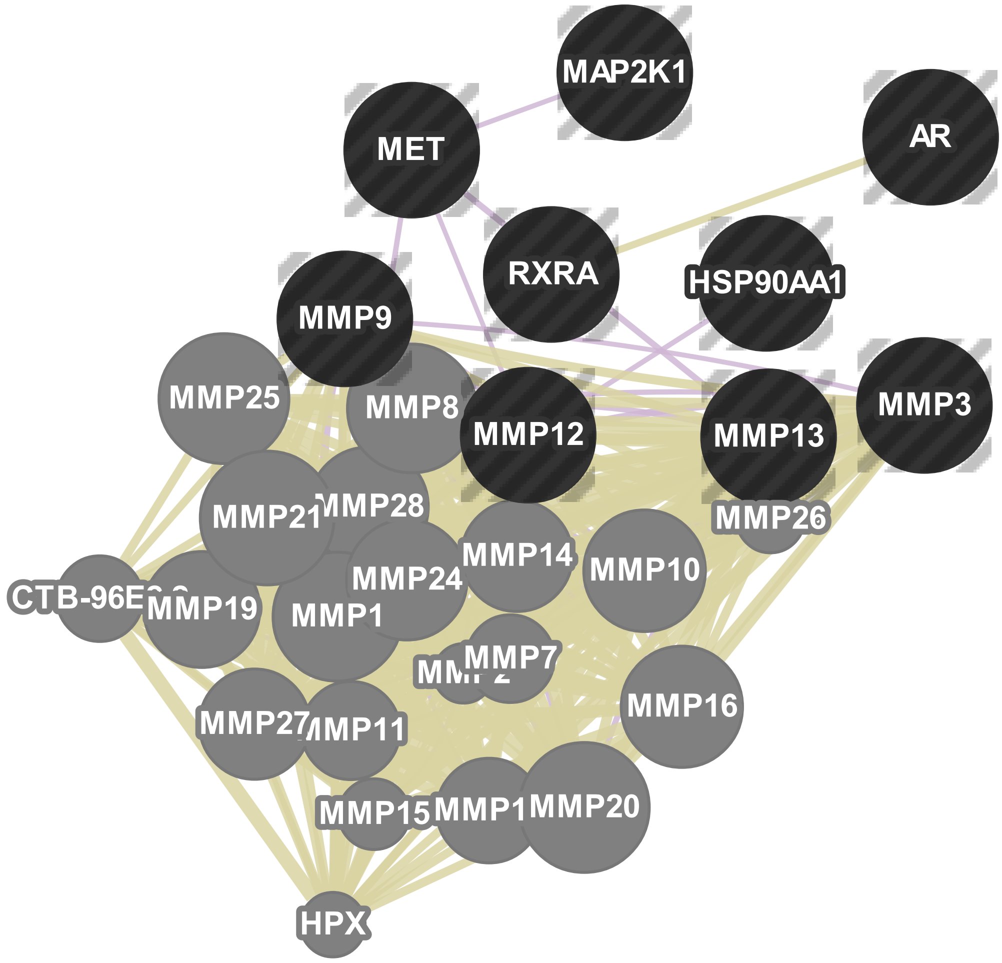

Functions legend
- query genes

Networks legend
- Co-expression
- Shared protein domains

Search parameters

Organism: H. sapiens (human)

Genes: P07900; P14780; P08581; P45452; P08254; Q02750; P10275; P19793; P39900

Networks:

Attributes:

Co-expression:
Alizadeh-Staudt-2000 Arijs-Rutgeerts-2009 Bahr-Bowler-2013 Bild-Nevins-2006 B Boldrick-Relman-2002 Burington-Shaughnessy-2008 Chen-Brown-2002 Cheok-Evans-2003 Gysin-McMahon-2012 Innocenti-Brown-2011 Kang-Willman-2010 Mallon-McKay-2013 Perou-Botstein-1999 Ramaswamy-Golub-2001 Rieger-Chu-2004 Roth-Zlotnik-2006 Salaverria-Siebert-2011 Smirnov-Cheung-2009 Wang-Maris-2006 Wu-Garvey-2007

Co-localization:
Johnson-Shoemaker-2003 Satoh-Yamamoto-2013 Schadt-Shoemaker-2004

Genetic interactions:
BIOGRID-SMALL-SCALE-STUDIES IREF-SMALL-SCALE-STUDIES Lin-Boeke-2012 A Lin-Boeke-2012 B Lin-Smith-2010 Toyoshima-Grandori-2012 Willingham-Muchowski-2003

Pathway:
PATHWAYCOMMONS-CELL_MAP PATHWAYCOMMONS-HUMANCYC PATHWAYCOMMONS-IMID PATHWAYCOMMONS-NCI_NATURE PATHWAYCOMMONS-REACTOME Wu-Stein-2010

Physical interactions:
Abu-Odeh-Aqeilan-2014 Agrawal-Sedivy-2010 Aichem-Groettrup-2012 Albers-Koegl-2005 Alexandru-Deshaies-2008 Altun-Kessler-2011 Andresen-Flores-Morales-2014 Arbuckle-Grant-2010 Arora-Mercola-2008 BIOGRID-SMALL-SCALE-STUDIES Bandyopadhyay-Ideker-2010 Bantscheff-Drewes-2011 Barr-Knapp-2009 Barrios-Rodiles-Wrana-2005 Behrends-Harper-2010 Behzadnia-Lührmann-2007 Bennett-Harper-2010 Benzinger-Hermeking-2005 Berggård-James-2006 Bett-Hay-2013 Blandin-Richard-2013 Bouwmeester-Superti-Furga-2004 Brajenovic-Drewes-2004 Brehme-Superti-Furga-2009 Bruderer-Hay-2011 Byron-Humphries-2012 Cai-Conaway-2007 Camargo-Brandon-2007 Cannavo-Jiricny-2007 Cao-Chinnaiyan-2014 Chen-Ge-2013 Chen-Naus-2012 Chen-Zhang-2013 Cheng-Chen-2010 Cloutier-Coulombe-2013 Colland-Gauthier-2004 Couzens-Gingras-2013 Cox-Rizzino-2013 Danielsen-Nielsen-2011 Dyer-Sobral-2010 Emanuele-Elledge-2011 Ewing-Figeys-2007 Fenner-Prehn-2010 Foerster-Ritter-2013 Foster-Marshall-2013 Freibaum-Taylor-2010 Gao-Reinberg-2012 Gautier-Hall-2009 Giannone-Liu-2010 Glatter-Gstaiger-2009 Gloeckner-Ueffing-2007 Goehler-Wanker-2004 Golebiowski-Hay-2009 Goudreault-Gingras-2009 Grant-2010 Greco-Cristea-2011 Havrylov-Redowicz-2009 Havugimana-Emili-2012 Hayes-Urbé-2012 Hegele-Stelzl-2012 A Hegele-Stelzl-2012 B Humphries-Humphries-2009 Hutchins-Peters-2010 IREF-BIND IREF-BIOGRID IREF-DIP IREF-HPRD IREF-INNATEDB IREF-INTACT IREF-MATRIXDB IREF-MPPI IREF-OPHID IREF-PUBMED IREF-SMALL-SCALE-STUDIES Ingham-Pawson-2005 Jeronimo-Coulombe-2007 Jin-Pawson-2004 Jones-MacBeath-2006 Joshi-Cristea-2013 Jäger-Krogan-2012 Kahle-Zoghbi-2011 Kim-Gygi-2011 Kneissl-Grummt-2003 Koch-Hermeking-2007 Kristensen-Foster-2012 Lau-Ronai-2012 Lee-Doedens-2011 Lee-Songyang-2011 Lehner-Sanderson-2004 A Lehner-Sanderson-2004 B Leng-Wang-2014 Li-Dorf-2011 A Li-Dorf-2011 B Lim-Zoghbi-2006 Liu-Wang-2012 Loch-Strickler-2012 Lopitz-Otsoa-Rodriguez-2012 Lu-Zhang-2013 Mak-Moffat-2010 Malovannaya-Qin-2010 Markson-Sanderson-2009 Maréchal-Zou-2014 Matafora-Bachi-2009 Matsumoto-Nakayama-2005 McCracken-Blencowe-2005 McFarland-Nussbaum-2008 Meek-Piwnica-Worms-2004 Meierhofer-Kaiser-2008 Miyamoto-Sato-Yanagawa-2010 Nakayama-Ohara-2002 Nakayasu-Adkins-2013 Napolitano-Meroni-2011 Nathan-Goldberg-2013 Neganova-Lako-2011 Newman-Keating-2003 Olma-Pintard-2009 Oláh-Ovádi-2011 Oshikawa-Nakayama-2012 Ouyang-Gill-2009 Panigrahi-Pati-2012 Perez-Hernandez-Yáñez-Mó-2013 Persaud-Rotin-2009 Pichlmair-Superti-Furga-2011 Pichlmair-Superti-Furga-2012 Pilot-Storck-Goillot-2010 Povlsen-Choudhary-2012 Ramachandran-LaBaer-2004 Ravasi-Hayashizaki-2010 Reinke-Keating-2013 Richter-Chrzanowska-Lightowlers-2010 Roux-Burke-2012 Rowbotham-Mermoud-2011 Roy-Parent-2013 Rual-Vidal-2005 A Rual-Vidal-2005 B San-Marina-Minden-2008 Sang-Jackson-2011 Sato-Conaway-2004 Shi-Qin-2011 Singh-Moore-2012 Soler-López-Aloy-2011 Sowa-Harper-2009 Stehling-Lill-2012 Stelzl-Wanker-2005 Suter-Wanker-2013 Taipale-Lindquist-2012 Takahashi-Conaway-2011 Tarallo-Weisz-2011 Tatham-Hay-2011 Teixeira-Gomes-2010 Thalappilly-Dusetti-2008 Tsai-Cristea-2012 Udeshi-Carr-2012 Vandamme-Angrand-2011 Vanderwerf-Bagby-2009 Varjosalo-Gstaiger-2013 A Varjosalo-Gstaiger-2013 B Varjosalo-Superti-Furga-2013 Venkatesan-Vidal-2009 Vinayagam-Wanker-2011 Wagner-Choudhary-2011 Wallach-Kramer-2013 Wang-Balch-2006 Wang-He-2008 Wang-Yang-2011 Weimann-Stelzl-2013 A Weimann-Stelzl-2013 B Weinmann-Meister-2009 Wilker-Yaffe-2007 Wong-O'Bryan-2012 Woods-Monteiro-2012 Woodsmith-Sanderson-2012 Wu-Li-2007 Wu-Ma-2012 Xiao-Lefkowitz-2007

Xie-Cong-2013 Xu-Jaffrey-2010 Xu-Ye-2012 Yang-Chen-2010 Yatim-Benkirane-2012 Yu-Chow-2013 Yu-Vidal-2011 Zanon-Pichler-2013 Zhang-Zou-2011 Zhao-Krug-2005 Zhao-Yang-2011 Zhou-Conrads-2004 Zhou-Liang-2012 de Hoog-Mann-2004 van Wijk-Timmers-2009

Predicted:
I2D-BIND-Fly2Human I2D-BIND-Mouse2Human I2D-BIND-Rat2Human I2D-BIND-Worm2Human I2D-BIND-Yeast2Human I2D-BioGRID-Fly2Human I2D-BioGRID-Mouse2Human I2D-BioGRID-Rat2Human I2D-BioGRID-Worm2Human I2D-BioGRID-Yeast2Human I2D-Chen-Pawson-2009-PiwiScreen-Mouse2Human I2D-Formstecher-Daviet-2005-Embryo-Fly2Human I2D-Giot-Rothbert-2003-Low-Fly2Human I2D-INNATEDB-Mouse2Human I2D-IntAct-Fly2Human I2D-IntAct-Mouse2Human I2D-IntAct-Rat2Human I2D-IntAct-Worm2Human I2D-IntAct-Yeast2Human I2D-Krogan-Greenblatt-2006-Core-Yeast2Human I2D-Krogan-Greenblatt-2006-NonCore-Yeast2Human I2D-Li-Vidal-2004-CORE-1-Worm2Human I2D-Li-Vidal-2004-non-core-Worm2Human I2D-MGI-Mouse2Human I2D-MINT-Fly2Human I2D-MINT-Mouse2Human I2D-MINT-Rat2Human I2D-MINT-Worm2Human I2D-MINT-Yeast2Human I2D-Manual-Mouse2Human I2D-Manual-Rat2Human I2D-Ptacek-Snyder-2005-Yeast2Human I2D-Tarassov-PCA-Yeast2Human I2D-Tewari-Vidal-2004-TGFb-Worm2Human I2D-Wang-Orkin-2006-EScmplx-Mouse2Human I2D-Wang-Orkin-2006-EScmplxlow-Mouse2Human I2D-Yu-Vidal-2008-GoldStd-Yeast2Human I2D-vonMering-Bork-2002-High-Yeast2Human I2D-vonMering-Bork-2002-Low-Yeast2Human I2D-vonMering-Bork-2002-Medium-Yeast2Human Stuart-Kim-2003

Shared protein domains:
INTERPRO PFAM

Network weighting: Automatically selected weighting method (Assigned based on query genes)

Number of gene results: 20

Networks

**Shared protein domains**	**67.23 %**
**PFAM**	**34.59 %**

Source: Pearson correlation with 543,464 interactions from Pfam

INTERPRO **32.64 %**

Source: Pearson correlation with 562,560 interactions from InterPro

Co-expression **32.77 %**

Arijs-Rutgeerts-2009 **8.33 %**

Mucosal gene expression of antimicrobial peptides in inflammatory bowel disease before and after first infliximab treatment. Arijs et al. (2009). *PLoS One*.

Source: Pearson correlation with 653,194 interactions from GEO

Tags: immune system

Wang-Maris-2006 **5.86 %**

Integrative genomics identifies distinct molecular classes of neuroblastoma and shows that multiple genes are targeted by regional alterations in DNA copy number. Wang et al. (2006). *Cancer Res*.

Source: Pearson correlation with 264,234 interactions from GEO

Tags: transcription factors; cancer

Ramaswamy-Golub-2001 **5.24 %**

Multiclass cancer diagnosis using tumor gene expression signatures. Ramaswamy et al. (2001). *Proc Natl Acad Sci U S A*.

Source: Pearson correlation with 270,142 interactions from supplementary material

Tags: cancer

Salaverria-Siebert-2011 **4.88 %**

Translocations activating IRF4 identify a subtype of germinal center-derived B-cell lymphoma affecting predominantly children and young adults. Salaverria et al. (2011). *Blood*.

Source: Pearson correlation with 514,070 interactions from GEO

Tags: transcription factors; cancer

Chen-Brown-2002 **3.70 %**

Gene expression patterns in human liver cancers. Chen et al. (2002). *Mol Biol Cell*.

Source: Pearson correlation with 275,649 interactions from supplementary material

Tags: cell proliferation; transcription factors; liver; cancer

Boldrick-Relman-2002 **2.44 %**

Stereotyped and specific gene expression programs in human innate immune responses to bacteria. Boldrick et al. (2002). *Proc Natl Acad Sci U S A*.

Source: Pearson correlation with 108,543 interactions from supplementary material

Tags: immune system

Alizadeh-Staudt-2000 **2.31 %**

Distinct types of diffuse large B-cell lymphoma identified by gene expression profiling. Alizadeh et al. (2000). *Nature*.

Source: Pearson correlation with 88,888 interactions from supplementary material

Tags: cultured cells; cancer

Attributes

Attribute	Gene

Genes

MMP13 (P45452) matrix metallopeptidase 13 (collagenase 3) [Source:HGNC Symbol;Acc:7159]

Functions:
1 2 3 4 5 6 7 8 9

Synonyms: ENSG00000137745; ENSG00000262325; ENSP00000260302; ENSP00000339672; ENSP00000460695; ENSP00000461440; 4322; MMP13; NP_002418; NM_002427; CLG3; MMP13_HUMAN; P45452;

More at Entrez

AR (P10275) androgen receptor [Source:HGNC Symbol;Acc:644]

Functions:
1

Synonyms: ENSG00000169083; ENSP00000363822; ENSP00000379358; ENSP00000379359; ENSP00000421155; ENSP00000425199; 367; AR; NP_000035; NP_001011645; NM_000044; NM_001011645; AIS; DHTR; HUMARA; NR3C4; SBMA; SMAX1; ANDR_HUMAN; P10275;

More at Entrez

MMP3 (P08254) matrix metallopeptidase 3 (stromelysin 1, progelatinase) [Source:HGNC Symbol;Acc:7173]

Functions:
1 2 3 4 5 6 7 8 9

Synonyms: ENSG00000149968; ENSG00000263313; ENSP00000299855; ENSP00000398346; ENSP00000435255; ENSP00000458496; ENSP00000459512; ENSP00000459712; 4314; MMP3; NP_002413; NM_002422; STMY; STMY1; MMP3_HUMAN; P08254;

More at Entrez

MMP12 (P39900) matrix metallopeptidase 12 (macrophage elastase) [Source:HGNC Symbol;Acc:7158]

Functions:
1 2 3 4 5 6 7 8 9 10 11 12

Synonyms: ENSG00000262406; ENSP00000458585; 4321; MMP12; NP_002417; NM_002426; HME; MMP12_HUMAN; P39900;

More at Entrez

RXRA (P19793) retinoid X receptor, alpha [Source:HGNC Symbol;Acc:10477]

Functions:
1

Synonyms: ENSG00000186350; ENSG00000269571; ENSP00000419692; ENSP00000442123; ENSP00000469111; ENSP00000470812; 6256; RXRA; NP_002948; NM_002957; NR2B1; P19793; RXRA_HUMAN;

More at Entrez

HSP90AA1 (P07900) heat shock protein 90kDa alpha (cytosolic), class A member 1 [Source:HGNC Symbol;Acc:5253]

Functions:
1

Synonyms: ENSG00000080824; ENSP00000216281; ENSP00000335153; ENSP00000396189; ENSP00000450712; ENSP00000451400; ENSP00000452241; 3320; HSP90AA1; NP_001017963; NP_005339; NM_001017963; NM_005348; FLJ31884; Hsp89; Hsp90; HSP90N; HSPC1; HSPCA; HS90A_HUMAN; P07900;

More at Entrez

MET (P08581) met proto-oncogene [Source:HGNC Symbol;Acc:7029]

Functions:
1

Synonyms: ENSG00000105976; ENSP00000317272; ENSP00000380860; ENSP00000398140;

ENSP00000398776; ENSP00000410980; ENSP00000413857; ENSP00000445020; 4233; MET; NP_000236; NP_001120972; NM_000245; NM_001127500; HGFR; RCCP2; MET_HUMAN; P08581;

More at Entrez

MMP9 (P14780) matrix metallopeptidase 9 (gelatinase B, 92kDa gelatinase, 92kDa type IV collagenase) [Source:HGN

Functions:
1 2 3 4 5 6 7 8 9 10 11 12

Synonyms: ENSG00000100985; ENSP00000361405; 4318; MMP9; NP_004985; NM_004994; CLG4B; MMP9_HUMAN; P14780;

More at Entrez

MAP2K1 (Q02750) mitogen-activated protein kinase kinase 1 [Source:HGNC Symbol;Acc:6840]

Functions:
1 15

Synonyms: ENSG00000169032; ENSP00000302486; ENSP00000456438; 5604; MAP2K1; NP_002746; NM_002755; MAPKK1; MEK1; PRKMK1; MP2K1_HUMAN; Q02750;

More at Entrez

MMP21 matrix metallopeptidase 21 [Source:HGNC Symbol;Acc:14357] 4.76

Functions:

Synonyms: ENSG00000154485; ENSP00000357798; 118856; MMP21; NP_671724; NM_147191; MMP21_HUMAN; Q8N119;

More at Entrez

MMP25 matrix metallopeptidase 25 [Source:HGNC Symbol;Acc:14246] 4.71

Functions:
13 14

Synonyms: ENSG00000008516; ENSP00000337816; 64386; MMP25; NP_071913; NM_022468; MMPL1; MT6-MMP; MMP25_HUMAN; Q9NPA2;

More at Entrez

MMP20 matrix metallopeptidase 20 [Source:HGNC Symbol;Acc:7167] 4.71

Functions:
2 3 4 5 6 7 8 9

Synonyms: ENSG00000137674; ENSP00000260228; 9313; MMP20; NP_004762; NM_004771; MMP20_HUMAN; O60882;

More at Entrez

MMP1 matrix metallopeptidase 1 (interstitial collagenase) [Source:HGNC Symbol;Acc:7155] 4.70

Functions:
2 3 4 5 6 7 8 9 10 11 12

Synonyms: ENSG00000196611; ENSP00000322788; 4312; MMP1; NP_001139410; NP_002412; NM_001145938; NM_002421; MMP1_HUMAN; P03956;

More at Entrez

MMP8 matrix metallopeptidase 8 (neutrophil collagenase) [Source:HGNC Symbol;Acc:7175] 4.69

Functions:
2 3 4 5 6 7 8 9 11

Synonyms: ENSG00000118113; ENSP00000236826; ENSP00000401004; ENSP00000431431; ENSP00000431938; ENSP00000433812; ENSP00000437173; 4317; MMP8; NP_002415; NM_002424; CLG1; MMP8_HUMAN; P22894;

More at Entrez

MMP10 matrix metallopeptidase 10 (stromelysin 2) [Source:HGNC Symbol;Acc:7156] 4.59

Functions:

	2	3	4	5	6	7	8	9						

Synonyms: ENSG00000166670; ENSP00000279441; ENSP00000441485; 4319; MMP10; NP_002416; NM_002425; STMY2; MMP10_HUMAN; P09238;

More at Entrez

MMP16 matrix metallopeptidase 16 (membrane-inserted) [Source:HGNC Symbol;Acc:7162] 4.58

Functions:

			4				8	9						15

Synonyms: ENSG00000156103; ENSP00000286614; ENSP00000429147; 4325; MMP16; NP_005932; NM_005941; C8orf57; DKFZp761D112; MT3-MMP; MMP16_HUMAN; P51512;

More at Entrez

MMP24 matrix metallopeptidase 24 (membrane-inserted) [Source:HGNC Symbol;Acc:7172] 4.57

Functions:

														15

Synonyms: ENSG00000125966; ENSP00000246186; 10893; MMP24; NP_006681; NM_006690; MT5-MMP; MMP24_HUMAN; Q9Y5R2;

More at Entrez

MMP28 matrix metallopeptidase 28 [Source:HGNC Symbol;Acc:14366] 4.55

Functions:

									10	11	12	13	14	

Synonyms: ENSG00000129270; ENSG00000271447; ENSP00000250144; ENSP00000464731; ENSP00000466155; ENSP00000466671; ENSP00000467034; ENSP00000468241; ENSP00000468249; ENSP00000468709; ENSP00000473853; 79148; MMP28; NP_001027449; NP_077278; NM_001032278; NM_024302; EPILYSIN; MM28; MMP-25; MMP-28; MMP28_HUMAN; Q9H239;

More at Entrez

MMP19 matrix metallopeptidase 19 [Source:HGNC Symbol;Acc:7165] 4.51

Functions:

	2	3	4	5	6	7	8	9				13	14	

Synonyms: ENSG00000123342; ENSP00000313437; ENSP00000377736; ENSP00000386625; ENSP00000446776; ENSP00000446979; ENSP00000447363; ENSP00000449752; 4327; MMP19; NP_001259030; NP_002420; NM_002429; MMP18; RASI-1; MMP19_HUMAN; Q99542;

More at Entrez

MMP14 matrix metallopeptidase 14 (membrane-inserted) [Source:HGNC Symbol;Acc:7160] 4.43

Functions:

	2	3	4	5	6	7	8	9						

Synonyms: ENSG00000157227; ENSP00000308208; ENSP00000446989; ENSP00000450323; 4323; MMP14; NP_004986; NM_004995; MT1-MMP; MMP14_HUMAN; P50281;

More at Entrez

MMP27 matrix metallopeptidase 27 [Source:HGNC Symbol;Acc:14250] 4.43

Functions:

Synonyms: ENSG00000137675; ENSP00000260229; 64066; MMP27; NP_071405; NM_022122; MMP27_HUMAN; Q9H306;

More at Entrez

MMP17 matrix metallopeptidase 17 (membrane-inserted) [Source:HGNC Symbol;Acc:7163] 4.34

Functions:

														15

Synonyms: ENSG00000198598; ENSP00000353767; ENSP00000439542; ENSP00000441106;

ENSP00000441710; ENSP00000442104; ENSP00000443727; ENSP00000444603; ENSP00000445620; 4326; MMP17; NP_057239; NM_016155; MT4-MMP; MMP17_HUMAN; Q9ULZ9;

More at Entrez

MMP11 matrix metallopeptidase 11 (stromelysin 3) [Source:HGNC Symbol;Acc:7157] 4.25

Functions:
2 3 4 5 6 7 8 9 13

Synonyms: ENSG00000099953; ENSP00000215743; ENSP00000408070; ENSP00000409860; ENSP00000412107; 4320; MMP11; NP_005931; NM_005940; STMY3; MMP11_HUMAN; P24347;

More at Entrez

MMP7 matrix metallopeptidase 7 (matrilysin, uterine) [Source:HGNC Symbol;Acc:7174] 4.10

Functions:
2 3 4 5 6 7 8 9

Synonyms: ENSG00000137673; ENSP00000260227; 4316; MMP7; NP_002414; NM_002423; MPSL1; PUMP-1; MMP7_HUMAN; P09237;

More at Entrez

CTB-96E2.2 Uncharacterized protein [Source:UniProtKB/TrEMBL;Acc:H0YJW9] 4.07

Functions:

Synonyms: ENSG00000262318; ENSG00000273171; ENSP00000452347; ENSP00000460756; CTB-96E2.2;

More at Ensembl

MMP15 matrix metallopeptidase 15 (membrane-inserted) [Source:HGNC Symbol;Acc:7161] 3.86

Functions:
2 3 4 5 6 7 8 9 15

Synonyms: ENSG00000102996; ENSP00000219271; ENSP00000457084; 4324; MMP15; NP_002419; NM_002428; MT2-MMP; MTMMP2; SMCP-2; MMP15_HUMAN; P51511;

More at Entrez

MMP26 matrix metallopeptidase 26 [Source:HGNC Symbol;Acc:14249] 3.81

Functions:
13 14

Synonyms: ENSG00000167346; ENSP00000300762; ENSP00000369753; 56547; MMP26; NP_068573; NM_021801; endometase; MGC126590; MGC126592; MMP26_HUMAN; Q9NRE1;

More at Entrez

HPX hemopexin [Source:HGNC Symbol;Acc:5171] 3.77

Functions:

Synonyms: ENSG00000110169; ENSP00000265983; 3263; HPX; NP_000604; NM_000613; HEMO_HUMAN; P02790;

More at Entrez

MMP2 matrix metallopeptidase 2 (gelatinase A, 72kDa gelatinase, 72kDa type IV collagenase) [Source:HGNC Sym 3.69

Functions:
2 3 4 5 6 7 8 9 11

Synonyms: ENSG00000087245; ENSP00000219070; ENSP00000394237; ENSP00000444143; ENSP00000456096; ENSP00000456518; ENSP00000457949; ENSP00000461421; ENSP00000461915; 4313; MMP2; NP_001121363; NP_004521; NM_001127891; NM_004530; CLG4; CLG4A; TBE-1; MMP2_HUMAN; P08253;

More at Entrez

Functions

Function	FDR	Coverage
1 query genes	n/a	9 / 9
2 collagen catabolic process	7.64E-24	14 / 65
3 multicellular organismal catabolic process	1.5E-23	14 / 71
4 extracellular matrix disassembly	1.3E-22	15 / 119
5 collagen metabolic process	1.4E-22	14 / 86
6 multicellular organismal macromolecule metabolic process	2.22E-22	14 / 90
7 multicellular organismal metabolic process	4.88E-22	14 / 96
8 extracellular matrix organization	5.03E-17	15 / 290
9 extracellular structure organization	5.03E-17	15 / 291
10 metalloendopeptidase activity	1.51E-4	4 / 27
11 endopeptidase activity	2.57E-4	6 / 163
12 metallopeptidase activity	1.05E-2	4 / 80
13 extracellular matrix	2.14E-2	5 / 208
14 proteinaceous extracellular matrix	3.15E-2	4 / 110
15 enzyme activator activity	3.43E-2	5 / 237

Interactions

Gene 1	Gene 2	Weight	Network group	Networks
MMP1	MMP12	0.10282072611153126	Co-expression	Arijs-Rutgeerts-2009<br>Ramaswamy-Golub-2001<br>Wang-Maris-2006
MMP1	MMP3	0.08665502583608031	Co-expression	Arijs-Rutgeerts-2009<br>Boldrick-Relman-2002<br>Ramaswamy-Golub-2001<br>Salaverria-Siebert-2011
MMP10	MMP1	0.07005718909204006	Co-expression	Arijs-Rutgeerts-2009<br>Ramaswamy-Golub-2001<br>Salaverria-Siebert-2011
MMP10	MMP3	0.06580262444913387	Co-expression	Arijs-Rutgeerts-2009<br>Salaverria-Siebert-2011
MMP9	MMP12	0.059718070551753044	Co-expression	Arijs-Rutgeerts-2009<br>Chen-Brown-2002<br>Wang-Maris-2006
MMP1	MMP9	0.05919322092086077	Co-expression	Alizadeh-Staudt-2000<br>Arijs-Rutgeerts-2009<br>Chen-Brown-2002<br>Wang-Maris-2006
MMP12	MMP3	0.043236750178039074	Co-expression	Arijs-Rutgeerts-2009<br>Boldrick-Relman-2002<br>Ramaswamy-Golub-2001
MMP10	MMP8	0.03840585984289646	Co-expression	Arijs-Rutgeerts-2009<br>Salaverria-Siebert-2011
MET	MMP13	0.027094952762126923	Co-expression	Salaverria-Siebert-2011
MMP7	MMP3	0.02531757391989231	Co-expression	Alizadeh-Staudt-2000
MMP7	MMP12	0.02388218231499195	Co-expression	Ramaswamy-Golub-2001
MMP20	MMP13	0.023868322372436523	Co-expression	Salaverria-Siebert-2011
MMP27	MMP1	0.022067232057452202	Co-expression	Chen-Brown-2002
MMP14	MMP3	0.02110777236521244	Co-expression	Alizadeh-Staudt-2000
MMP8	MMP13	0.021025409922003746	Co-expression	Arijs-Rutgeerts-2009
MMP8	MMP1	0.020457519218325615	Co-expression	Arijs-Rutgeerts-2009
MMP2	MMP1	0.020202286541461945	Co-expression	Alizadeh-Staudt-2000<br>Salaverria-Siebert-2011
MMP8	MMP20	0.01942802220582962	Co-expression	Wang-Maris-2006
MMP10	MMP12	0.019088277593255043	Co-expression	Arijs-Rutgeerts-2009
MMP8	MMP9	0.019027601927518845	Co-expression	Arijs-Rutgeerts-2009
MMP7	MMP14	0.01883828453719616	Co-expression	Salaverria-Siebert-2011
MMP8	MMP12	0.017592325806617737	Co-expression	Arijs-Rutgeerts-2009
MMP10	MMP9	0.016696784645318985	Co-expression	Arijs-Rutgeerts-2009
MMP20	MMP9	0.01613892987370491	Co-expression	Wang-Maris-2006
MMP10	MMP13	0.015926940366625786	Co-expression	Ramaswamy-Golub-2001
MMP7	MMP10	0.015549114905297756	Co-expression	Arijs-Rutgeerts-2009
MMP1	MET	0.015004313550889492	Co-expression	Salaverria-Siebert-2011
MMP8	MMP3	0.01430840790271759	Co-expression	Arijs-Rutgeerts-2009
MMP7	MMP11	0.01417256984859705	Co-expression	Ramaswamy-Golub-2001
HSP90AA1	MMP12	0.012880648486316204	Co-expression	Alizadeh-Staudt-2000<br>Boldrick-Relman-2002
MAP2K1	MET	0.010279510170221329	Co-expression	Arijs-Rutgeerts-2009
MMP14	MMP19	0.01020832546055317	Co-expression	Wang-Maris-2006
MMP9	MMP3	0.009068215265870094	Co-expression	Arijs-Rutgeerts-2009
MMP2	MMP15	0.009007873013615608	Co-expression	Alizadeh-Staudt-2000
MMP17	MMP24	0.008745565079152584	Co-expression	Salaverria-Siebert-2011
MMP15	MMP9	0.008615892380475998	Co-expression	Alizadeh-Staudt-2000
MMP2	MMP19	0.008321760222315788	Co-expression	Salaverria-Siebert-2011
MMP2	MMP14	0.00780183169990778	Co-expression	Salaverria-Siebert-2011

MMP2	MMP7	0.007076840847730637	Co-expression	Salaverria-Siebert-2011
MET	MMP12	0.0065786163322627544	Co-expression	Alizadeh-Staudt-2000
MMP2	MMP9	0.006450654938817024	Co-expression	Alizadeh-Staudt-2000
MMP2	MMP3	0.005963992793112993	Co-expression	Salaverria-Siebert-2011
MMP26	MMP27	0.0031631295569241047	Co-expression	Arijs-Rutgeerts-2009
HPX	CTB-96E2.2	0.1462436467409134	Shared protein domains	INTERPRO<br>PFAM
MMP24	MMP16	0.11205314472317696	Shared protein domains	INTERPRO<br>PFAM
MMP14	MMP16	0.11205314472317696	Shared protein domains	INTERPRO<br>PFAM
MMP14	MMP24	0.11205314472317696	Shared protein domains	INTERPRO<br>PFAM
MMP15	MMP16	0.10263661295175552	Shared protein domains	INTERPRO<br>PFAM
MMP15	MMP24	0.10263661295175552	Shared protein domains	INTERPRO<br>PFAM
MMP15	MMP14	0.10263661295175552	Shared protein domains	INTERPRO<br>PFAM
MMP26	MMP7	0.09782937541604042	Shared protein domains	INTERPRO<br>PFAM
MMP19	MMP20	0.08680696785449982	Shared protein domains	INTERPRO<br>PFAM
MMP17	MMP20	0.08680696785449982	Shared protein domains	INTERPRO<br>PFAM
MMP17	MMP19	0.08680696785449982	Shared protein domains	INTERPRO<br>PFAM
MMP20	MMP25	0.08679590746760368	Shared protein domains	INTERPRO<br>PFAM
MMP19	MMP25	0.08679590746760368	Shared protein domains	INTERPRO<br>PFAM
MMP17	MMP25	0.08679590746760368	Shared protein domains	INTERPRO<br>PFAM
MMP20	MMP13	0.08678418025374413	Shared protein domains	INTERPRO<br>PFAM
MMP19	MMP13	0.08678418025374413	Shared protein domains	INTERPRO<br>PFAM
MMP17	MMP13	0.08678418025374413	Shared protein domains	INTERPRO<br>PFAM
MMP25	MMP13	0.08677312359213829	Shared protein domains	INTERPRO<br>PFAM
MMP20	MMP12	0.08663327991962433	Shared protein domains	INTERPRO<br>PFAM
MMP19	MMP12	0.08663327991962433	Shared protein domains	INTERPRO<br>PFAM
MMP17	MMP12	0.08663327991962433	Shared protein domains	INTERPRO<br>PFAM
MMP25	MMP12	0.08662226796150208	Shared protein domains	INTERPRO<br>PFAM
MMP12	MMP13	0.0866105854511261	Shared protein domains	INTERPRO<br>PFAM
MMP27	MMP20	0.08649903163313866	Shared protein domains	INTERPRO<br>PFAM
MMP27	MMP19	0.08649903163313866	Shared protein domains	INTERPRO<br>PFAM
MMP17	MMP27	0.08649903163313866	Shared protein domains	INTERPRO<br>PFAM
MMP27	MMP25	0.08648804947733879	Shared protein domains	INTERPRO<br>PFAM
MMP27	MMP13	0.0864764079451561	Shared protein domains	INTERPRO<br>PFAM
MMP20	MMP21	0.08641837537288666	Shared protein domains	INTERPRO

				PFAM
MMP19	MMP21	0.08641837537288666	Shared protein domains	INTERPRO PFAM
MMP17	MMP21	0.08641837537288666	Shared protein domains	INTERPRO PFAM
MMP25	MMP21	0.08640741556882858	Shared protein domains	INTERPRO PFAM
MMP21	MMP13	0.08639579266309738	Shared protein domains	INTERPRO PFAM
MMP20	MMP3	0.08634748309850693	Shared protein domains	INTERPRO PFAM
MMP19	MMP3	0.08634748309850693	Shared protein domains	INTERPRO PFAM
MMP17	MMP3	0.08634748309850693	Shared protein domains	INTERPRO PFAM
MMP8	MMP20	0.08633934706449509	Shared protein domains	INTERPRO PFAM
MMP19	MMP8	0.08633934706449509	Shared protein domains	INTERPRO PFAM
MMP17	MMP8	0.08633934706449509	Shared protein domains	INTERPRO PFAM
MMP25	MMP3	0.08633654192090034	Shared protein domains	INTERPRO PFAM
MMP8	MMP25	0.0863284096121788	Shared protein domains	INTERPRO PFAM
MMP27	MMP12	0.08632660284638405	Shared protein domains	INTERPRO PFAM
MMP3	MMP13	0.08632494136691093	Shared protein domains	INTERPRO PFAM
MMP8	MMP13	0.08631680905818939	Shared protein domains	INTERPRO PFAM
MMP1	MMP20	0.08628861606121063	Shared protein domains	INTERPRO PFAM
MMP19	MMP1	0.08628861606121063	Shared protein domains	INTERPRO PFAM
MMP17	MMP1	0.08628861606121063	Shared protein domains	INTERPRO PFAM
MMP1	MMP25	0.08627768978476524	Shared protein domains	INTERPRO PFAM
MMP1	MMP13	0.08626610413193703	Shared protein domains	INTERPRO PFAM
MMP21	MMP12	0.08624627441167831	Shared protein domains	INTERPRO PFAM
MMP12	MMP3	0.08617567270994186	Shared protein domains	INTERPRO PFAM
MMP8	MMP12	0.08616757020354271	Shared protein domains	INTERPRO PFAM
MMP1	MMP12	0.08611704409122467	Shared protein domains	INTERPRO PFAM
MMP27	MMP21	0.08611324802041054	Shared protein domains	INTERPRO PFAM
MMP27	MMP3	0.086042869836092	Shared protein domains	INTERPRO PFAM
MMP27	MMP8	0.08603479340672493	Shared protein domains	INTERPRO PFAM
MMP27	MMP1	0.08598442748188972	Shared protein domains	INTERPRO PFAM
MMP21	MMP3	0.08596308156847954	Shared protein domains	INTERPRO PFAM
MMP8	MMP21	0.08595502376556396	Shared protein domains	INTERPRO PFAM
MMP1	MMP21	0.08590475097298622	Shared protein domains	INTERPRO

				PFAM
MMP8	MMP3	0.08588491007685661	Shared protein domains	INTERPRO PFAM
MMP1	MMP3	0.08583472296595573	Shared protein domains	INTERPRO PFAM
MMP8	MMP1	0.08582668751478195	Shared protein domains	INTERPRO PFAM
MMP10	MMP20	0.08571403473615646	Shared protein domains	INTERPRO PFAM
MMP19	MMP10	0.08571403473615646	Shared protein domains	INTERPRO PFAM
MMP17	MMP10	0.08571403473615646	Shared protein domains	INTERPRO PFAM
MMP10	MMP25	0.08570325374603271	Shared protein domains	INTERPRO PFAM
MMP10	MMP13	0.08569182828068733	Shared protein domains	INTERPRO PFAM
MMP10	MMP12	0.08554480597376823	Shared protein domains	INTERPRO PFAM
MMP27	MMP10	0.08541399985551834	Shared protein domains	INTERPRO PFAM
MMP10	MMP21	0.08533541485667229	Shared protein domains	INTERPRO PFAM
MMP10	MMP3	0.0852663442492485	Shared protein domains	INTERPRO PFAM
MMP10	MMP8	0.08525841683149338	Shared protein domains	INTERPRO PFAM
MMP10	MMP1	0.08520898595452309	Shared protein domains	INTERPRO PFAM
MMP16	MMP20	0.0835627056658268	Shared protein domains	INTERPRO PFAM
MMP24	MMP20	0.0835627056658268	Shared protein domains	INTERPRO PFAM
MMP19	MMP16	0.0835627056658268	Shared protein domains	INTERPRO PFAM
MMP19	MMP24	0.0835627056658268	Shared protein domains	INTERPRO PFAM
MMP14	MMP20	0.0835627056658268	Shared protein domains	INTERPRO PFAM
MMP14	MMP19	0.0835627056658268	Shared protein domains	INTERPRO PFAM
MMP17	MMP16	0.0835627056658268	Shared protein domains	INTERPRO PFAM
MMP17	MMP24	0.0835627056658268	Shared protein domains	INTERPRO PFAM
MMP17	MMP14	0.0835627056658268	Shared protein domains	INTERPRO PFAM
MMP16	MMP25	0.08355189487338066	Shared protein domains	INTERPRO PFAM
MMP24	MMP25	0.08355189487338066	Shared protein domains	INTERPRO PFAM
MMP14	MMP25	0.08355189487338066	Shared protein domains	INTERPRO PFAM
MMP16	MMP13	0.083540428429842	Shared protein domains	INTERPRO PFAM
MMP24	MMP13	0.083540428429842	Shared protein domains	INTERPRO PFAM
MMP14	MMP13	0.083540428429842	Shared protein domains	INTERPRO PFAM
MMP16	MMP12	0.0833929292857647	Shared protein domains	INTERPRO PFAM
MMP24	MMP12	0.0833929292857647	Shared protein domains	INTERPRO PFAM

MMP14	MMP12	0.0833929292857647	Shared protein domains	INTERPRO PFAM
MMP27	MMP16	0.08326169848442078	Shared protein domains	INTERPRO PFAM
MMP27	MMP24	0.08326169848442078	Shared protein domains	INTERPRO PFAM
MMP27	MMP14	0.08326169848442078	Shared protein domains	INTERPRO PFAM
MMP16	MMP21	0.08318285644054413	Shared protein domains	INTERPRO PFAM
MMP24	MMP21	0.08318285644054413	Shared protein domains	INTERPRO PFAM
MMP14	MMP21	0.08318285644054413	Shared protein domains	INTERPRO PFAM
MMP16	MMP3	0.08311356231570244	Shared protein domains	INTERPRO PFAM
MMP24	MMP3	0.08311356231570244	Shared protein domains	INTERPRO PFAM
MMP14	MMP3	0.08311356231570244	Shared protein domains	INTERPRO PFAM
MMP16	MMP8	0.08310561254620552	Shared protein domains	INTERPRO PFAM
MMP24	MMP8	0.08310561254620552	Shared protein domains	INTERPRO PFAM
MMP14	MMP8	0.08310561254620552	Shared protein domains	INTERPRO PFAM
MMP16	MMP1	0.0830560214817524	Shared protein domains	INTERPRO PFAM
MMP24	MMP1	0.0830560214817524	Shared protein domains	INTERPRO PFAM
MMP14	MMP1	0.0830560214817524	Shared protein domains	INTERPRO PFAM
MMP2	MMP9	0.08281752467155457	Shared protein domains	INTERPRO PFAM
MMP16	MMP10	0.08249437436461449	Shared protein domains	INTERPRO PFAM
MMP24	MMP10	0.08249437436461449	Shared protein domains	INTERPRO PFAM
MMP14	MMP10	0.08249437436461449	Shared protein domains	INTERPRO PFAM
MMP28	MMP20	0.08162881433963776	Shared protein domains	INTERPRO PFAM
MMP19	MMP28	0.08162881433963776	Shared protein domains	INTERPRO PFAM
MMP17	MMP28	0.08162881433963776	Shared protein domains	INTERPRO PFAM
MMP28	MMP25	0.08161871135234833	Shared protein domains	INTERPRO PFAM
MMP28	MMP13	0.08160799741744995	Shared protein domains	INTERPRO PFAM
MMP28	MMP12	0.08147017657756805	Shared protein domains	INTERPRO PFAM
MMP27	MMP28	0.08134755864739418	Shared protein domains	INTERPRO PFAM
MMP28	MMP21	0.0812738947570324	Shared protein domains	INTERPRO PFAM
MMP28	MMP3	0.08120914548635483	Shared protein domains	INTERPRO PFAM
MMP28	MMP8	0.0812017135322094	Shared protein domains	INTERPRO PFAM
MMP28	MMP1	0.08115537837147713	Shared protein domains	INTERPRO PFAM

MMP28	MMP10	0.0806305892765522	Shared protein domains	INTERPRO<br>PFAM
MMP28	MMP16	0.07854435965418816	Shared protein domains	INTERPRO<br>PFAM
MMP28	MMP24	0.07854435965418816	Shared protein domains	INTERPRO<br>PFAM
MMP14	MMP28	0.07854435965418816	Shared protein domains	INTERPRO<br>PFAM
MMP11	MMP20	0.07805588468909264	Shared protein domains	INTERPRO<br>PFAM
MMP11	MMP19	0.07805588468909264	Shared protein domains	INTERPRO<br>PFAM
MMP11	MMP17	0.07805588468909264	Shared protein domains	INTERPRO<br>PFAM
MMP11	MMP25	0.07804539054632187	Shared protein domains	INTERPRO<br>PFAM
MMP11	MMP13	0.07803426682949066	Shared protein domains	INTERPRO<br>PFAM
MMP11	MMP12	0.07789112254977226	Shared protein domains	INTERPRO<br>PFAM
MMP11	MMP27	0.07776376977562904	Shared protein domains	INTERPRO<br>PFAM
MMP11	MMP21	0.07768725603818893	Shared protein domains	INTERPRO<br>PFAM
MMP11	MMP3	0.0776200033724308	Shared protein domains	INTERPRO<br>PFAM
MMP11	MMP8	0.07761228829622269	Shared protein domains	INTERPRO<br>PFAM
MMP11	MMP1	0.07756416127085686	Shared protein domains	INTERPRO<br>PFAM
MMP7	MMP20	0.07719079032540321	Shared protein domains	INTERPRO<br>PFAM
MMP7	MMP19	0.07719079032540321	Shared protein domains	INTERPRO<br>PFAM
MMP7	MMP17	0.07719079032540321	Shared protein domains	INTERPRO<br>PFAM
MMP7	MMP25	0.07718152925372124	Shared protein domains	INTERPRO<br>PFAM
MMP7	MMP13	0.0771716982126236	Shared protein domains	INTERPRO<br>PFAM
MMP7	MMP12	0.07704530656337738	Shared protein domains	INTERPRO<br>PFAM
MMP11	MMP10	0.077019102871418	Shared protein domains	INTERPRO<br>PFAM
MMP7	MMP27	0.07693285122513771	Shared protein domains	INTERPRO<br>PFAM
MMP7	MMP21	0.0768652856349945	Shared protein domains	INTERPRO<br>PFAM
MMP7	MMP3	0.07680590450763702	Shared protein domains	INTERPRO<br>PFAM
MMP7	MMP8	0.07679909467697144	Shared protein domains	INTERPRO<br>PFAM
MMP7	MMP1	0.07675659656524658	Shared protein domains	INTERPRO<br>PFAM
MMP15	MMP20	0.07667861878871918	Shared protein domains	INTERPRO<br>PFAM
MMP15	MMP19	0.07667861878871918	Shared protein domains	INTERPRO<br>PFAM
MMP15	MMP17	0.07667861878871918	Shared protein domains	INTERPRO<br>PFAM
MMP15	MMP25	0.07666851952672005	Shared protein domains	INTERPRO<br>PFAM

MMP15	MMP13	0.07665780559182167	Shared protein domains	INTERPRO PFAM
MMP15	MMP12	0.07651997357606888	Shared protein domains	INTERPRO PFAM
MMP15	MMP27	0.07639734819531441	Shared protein domains	INTERPRO PFAM
MMP15	MMP21	0.07632367685437202	Shared protein domains	INTERPRO PFAM
MMP7	MMP10	0.0762753002345562	Shared protein domains	INTERPRO PFAM
MMP15	MMP3	0.07625892385840416	Shared protein domains	INTERPRO PFAM
MMP15	MMP8	0.07625149190425873	Shared protein domains	INTERPRO PFAM
MMP15	MMP1	0.07620515301823616	Shared protein domains	INTERPRO PFAM
MMP15	MMP10	0.07568032294511795	Shared protein domains	INTERPRO PFAM
MMP11	MMP16	0.07519971951842308	Shared protein domains	INTERPRO PFAM
MMP11	MMP24	0.07519971951842308	Shared protein domains	INTERPRO PFAM
MMP11	MMP14	0.07519971951842308	Shared protein domains	INTERPRO PFAM
CTB-96E2.2	MMP28	0.0751018114387989	Shared protein domains	INTERPRO PFAM
HPX	MMP28	0.07510174438357353	Shared protein domains	INTERPRO PFAM
CTB-96E2.2	MMP11	0.07482390105724335	Shared protein domains	INTERPRO PFAM
HPX	MMP11	0.07482384517788887	Shared protein domains	INTERPRO PFAM
MMP7	MMP16	0.07424148172140121	Shared protein domains	INTERPRO PFAM
MMP7	MMP24	0.07424148172140121	Shared protein domains	INTERPRO PFAM
MMP7	MMP14	0.07424148172140121	Shared protein domains	INTERPRO PFAM
MMP15	MMP28	0.07203641533851624	Shared protein domains	INTERPRO PFAM
MMP26	MMP11	0.07163614593446255	Shared protein domains	INTERPRO PFAM
MMP20	MMP9	0.06948774307966232	Shared protein domains	INTERPRO PFAM
MMP19	MMP9	0.06948774307966232	Shared protein domains	INTERPRO PFAM
MMP17	MMP9	0.06948774307966232	Shared protein domains	INTERPRO PFAM
MMP2	MMP20	0.06948769092559814	Shared protein domains	INTERPRO PFAM
MMP2	MMP19	0.06948769092559814	Shared protein domains	INTERPRO PFAM
MMP2	MMP17	0.06948769092559814	Shared protein domains	INTERPRO PFAM
MMP25	MMP9	0.06947918981313705	Shared protein domains	INTERPRO PFAM
MMP2	MMP25	0.06947914138436317	Shared protein domains	INTERPRO PFAM
MMP9	MMP13	0.0694701261818409	Shared protein domains	INTERPRO PFAM
MMP2	MMP13	0.06947007402777672	Shared protein domains	INTERPRO PFAM

MMP9	MMP12	0.06935350596904755	Shared protein domains	INTERPRO PFAM
MMP2	MMP12	0.06935345754027367	Shared protein domains	INTERPRO PFAM
MMP27	MMP9	0.06924974918365479	Shared protein domains	INTERPRO PFAM
MMP2	MMP27	0.0692497007548809	Shared protein domains	INTERPRO PFAM
MMP21	MMP9	0.06918741390109062	Shared protein domains	INTERPRO PFAM
MMP2	MMP21	0.06918736174702644	Shared protein domains	INTERPRO PFAM
MMP9	MMP3	0.06913262233138084	Shared protein domains	INTERPRO PFAM
MMP2	MMP3	0.06913257390260696	Shared protein domains	INTERPRO PFAM
MMP8	MMP9	0.06912633776664734	Shared protein domains	INTERPRO PFAM
MMP2	MMP8	0.06912628933787346	Shared protein domains	INTERPRO PFAM
MMP1	MMP9	0.06908712908625603	Shared protein domains	INTERPRO PFAM
MMP2	MMP1	0.06908708065748215	Shared protein domains	INTERPRO PFAM
MMP15	MMP11	0.06907225027680397	Shared protein domains	INTERPRO PFAM
MMP10	MMP9	0.06864306330680847	Shared protein domains	INTERPRO PFAM
MMP2	MMP10	0.0686430111527443	Shared protein domains	INTERPRO PFAM
MMP26	MMP20	0.06816204264760017	Shared protein domains	INTERPRO PFAM
MMP26	MMP19	0.06816204264760017	Shared protein domains	INTERPRO PFAM
MMP26	MMP17	0.06816204264760017	Shared protein domains	INTERPRO PFAM
MMP26	MMP25	0.06815265864133835	Shared protein domains	INTERPRO PFAM
MMP26	MMP13	0.06814270839095116	Shared protein domains	INTERPRO PFAM
MMP15	MMP7	0.06805405765771866	Shared protein domains	INTERPRO PFAM
MMP26	MMP12	0.06801469251513481	Shared protein domains	INTERPRO PFAM
MMP26	MMP27	0.06790079548954964	Shared protein domains	INTERPRO PFAM
MMP26	MMP21	0.06783236563205719	Shared protein domains	INTERPRO PFAM
MMP26	MMP3	0.06777221709489822	Shared protein domains	INTERPRO PFAM
MMP26	MMP8	0.06776531785726547	Shared protein domains	INTERPRO PFAM
MMP26	MMP1	0.06772227585315704	Shared protein domains	INTERPRO PFAM
MMP26	MMP10	0.06723480299115181	Shared protein domains	INTERPRO PFAM
MMP28	MMP9	0.06704460456967354	Shared protein domains	INTERPRO PFAM
MMP2	MMP28	0.06704459711909294	Shared protein domains	INTERPRO PFAM
MMP2	MMP7	0.06695974990725517	Shared protein domains	INTERPRO PFAM
MMP7	MMP9	0.06695970147848129	Shared protein domains	INTERPRO

				PFAM
MMP11	MMP28	0.06691209971904755	Shared protein domains	INTERPRO PFAM
MMP16	MMP9	0.06685641780495644	Shared protein domains	INTERPRO PFAM
MMP24	MMP9	0.06685641780495644	Shared protein domains	INTERPRO PFAM
MMP14	MMP9	0.06685641780495644	Shared protein domains	INTERPRO PFAM
MMP2	MMP16	0.06685636937618256	Shared protein domains	INTERPRO PFAM
MMP2	MMP24	0.06685636937618256	Shared protein domains	INTERPRO PFAM
MMP2	MMP14	0.06685636937618256	Shared protein domains	INTERPRO PFAM
MMP26	MMP16	0.065691988915205	Shared protein domains	INTERPRO PFAM
MMP26	MMP24	0.065691988915205	Shared protein domains	INTERPRO PFAM
MMP26	MMP14	0.065691988915205	Shared protein domains	INTERPRO PFAM
MMP7	MMP28	0.06513834558427334	Shared protein domains	INTERPRO PFAM
CTB-96E2.2	MMP20	0.06170181185007095	Shared protein domains	INTERPRO PFAM
CTB-96E2.2	MMP19	0.06170181185007095	Shared protein domains	INTERPRO PFAM
CTB-96E2.2	MMP17	0.06170181185007095	Shared protein domains	INTERPRO PFAM
HPX	MMP20	0.061701761558651924	Shared protein domains	INTERPRO PFAM
HPX	MMP19	0.061701761558651924	Shared protein domains	INTERPRO PFAM
HPX	MMP17	0.061701761558651924	Shared protein domains	INTERPRO PFAM
CTB-96E2.2	MMP25	0.061694083735346794	Shared protein domains	INTERPRO PFAM
HPX	MMP25	0.061694033443927765	Shared protein domains	INTERPRO PFAM
CTB-96E2.2	MMP13	0.06168588809669018	Shared protein domains	INTERPRO PFAM
HPX	MMP13	0.06168584153056145	Shared protein domains	INTERPRO PFAM
CTB-96E2.2	MMP12	0.06158047541975975	Shared protein domains	INTERPRO PFAM
HPX	MMP12	0.06158042699098587	Shared protein domains	INTERPRO PFAM
CTB-96E2.2	MMP27	0.06148669123649597	Shared protein domains	INTERPRO PFAM
HPX	MMP27	0.06148664280772209	Shared protein domains	INTERPRO PFAM
CTB-96E2.2	MMP21	0.061430344358086586	Shared protein domains	INTERPRO PFAM
HPX	MMP21	0.0614302940666756	Shared protein domains	INTERPRO PFAM
MMP2	MMP11	0.06138291954994202	Shared protein domains	INTERPRO PFAM
MMP11	MMP9	0.06138289161026478	Shared protein domains	INTERPRO PFAM
CTB-96E2.2	MMP3	0.06138082034885883	Shared protein domains	INTERPRO PFAM
HPX	MMP3	0.06138077192008495	Shared protein domains	INTERPRO PFAM

CTB-96E2.2	MMP8	0.06137513741850853	Shared protein domains	INTERPRO PFAM
HPX	MMP8	0.06137508898973465	Shared protein domains	INTERPRO PFAM
CTB-96E2.2	MMP1	0.06133969500660896	Shared protein domains	INTERPRO PFAM
HPX	MMP1	0.06133964844048023	Shared protein domains	INTERPRO PFAM
MMP15	MMP9	0.06131063774228096	Shared protein domains	INTERPRO PFAM
MMP2	MMP15	0.06131059117615223	Shared protein domains	INTERPRO PFAM
CTB-96E2.2	MMP10	0.06093830242753029	Shared protein domains	INTERPRO PFAM
HPX	MMP10	0.06093825399875641	Shared protein domains	INTERPRO PFAM
MMP26	MMP15	0.060366034507751465	Shared protein domains	INTERPRO PFAM
MMP2	MMP26	0.05953052453696728	Shared protein domains	INTERPRO PFAM
MMP26	MMP9	0.059530315920710564	Shared protein domains	INTERPRO PFAM
CTB-96E2.2	MMP16	0.0593793373554945	Shared protein domains	INTERPRO PFAM
CTB-96E2.2	MMP24	0.0593793373554945	Shared protein domains	INTERPRO PFAM
CTB-96E2.2	MMP14	0.0593793373554945	Shared protein domains	INTERPRO PFAM
HPX	MMP16	0.05937929078936577	Shared protein domains	INTERPRO PFAM
HPX	MMP24	0.05937929078936577	Shared protein domains	INTERPRO PFAM
HPX	MMP14	0.05937929078936577	Shared protein domains	INTERPRO PFAM
MMP7	MMP11	0.05570451356470585	Shared protein domains	INTERPRO PFAM
MMP15	CTB-96E2.2	0.05446958169341087	Shared protein domains	INTERPRO PFAM
HPX	MMP15	0.05446953698992729	Shared protein domains	INTERPRO PFAM
MMP2	CTB-96E2.2	0.053628357127308846	Shared protein domains	INTERPRO PFAM
MMP2	HPX	0.053628310561180115	Shared protein domains	INTERPRO PFAM
CTB-96E2.2	MMP9	0.05362806096673012	Shared protein domains	INTERPRO PFAM
HPX	MMP9	0.053628016263246536	Shared protein domains	INTERPRO PFAM
MMP26	MMP28	0.047662071883678436	Shared protein domains	INTERPRO PFAM
RXRA	AR	0.041201964020729065	Shared protein domains	INTERPRO PFAM

Search results generated by the GeneMANIA algorithm (genemania.org)

GeneMANIA

Created on: 23 July 2015 10:27:48
Last database update: 1 June 2014 20:00:00
Application version: 3.1.2.8

Report of GeneMANIA search

Network image

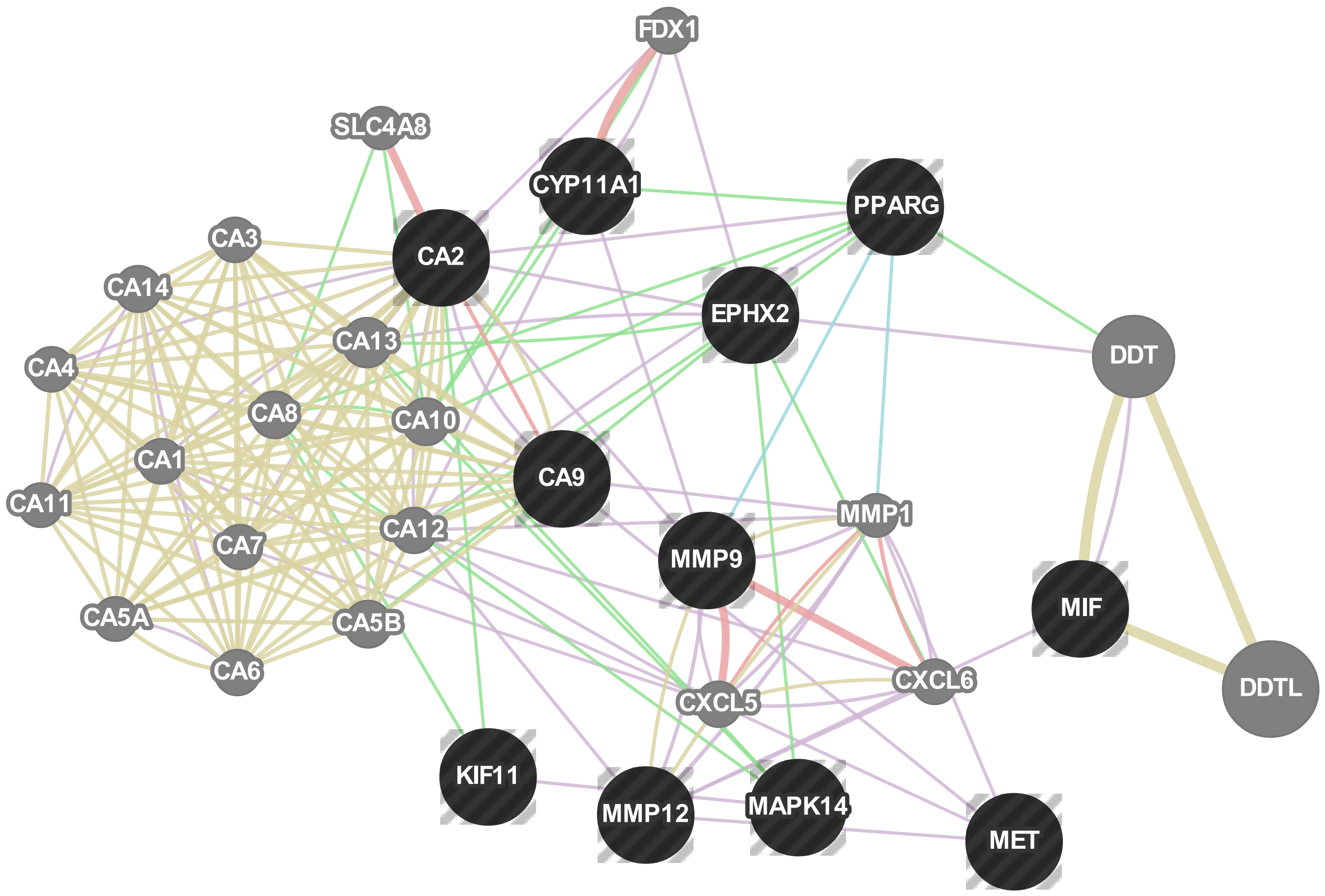

Functions legend
- query genes

Networks legend
- Co-expression
- Genetic interactions
- Pathway
- Physical interactions
- Shared protein domains

Search parameters

Organism: H. sapiens (human)

Genes: P14780; P00918; P08581; Q16790; Q16539; P34913; P37231; P52732; P05108; P14174; P39900

Networks:

Attributes:

Co-expression:
Alizadeh-Staudt-2000 Arijs-Rutgeerts-2009 Bahr-Bowler-2013 Bild-Nevins-2006 B Boldrick-Relman-2002 Burington-Shaughnessy-2008 Chen-Brown-2002 Cheok-Evans-2003 Gysin-McMahon-2012 Innocenti-Brown-2011 Kang-Willman-2010 Mallon-McKay-2013 Perou-Botstein-1999 Ramaswamy-Golub-2001 Rieger-Chu-2004 Roth-Zlotnik-2006 Salaverria-Siebert-2011 Smirnov-Cheung-2009 Wang-Maris-2006 Wu-Garvey-2007

Co-localization:
Johnson-Shoemaker-2003 Satoh-Yamamoto-2013 Schadt-Shoemaker-2004

Genetic interactions:
BIOGRID-SMALL-SCALE-STUDIES IREF-SMALL-SCALE-STUDIES Lin-Boeke-2012 A Lin-Boeke-2012 B Lin-Smith-2010 Toyoshima-Grandori-2012 Willingham-Muchowski-2003

Pathway:
PATHWAYCOMMONS-CELL_MAP PATHWAYCOMMONS-HUMANCYC PATHWAYCOMMONS-IMID PATHWAYCOMMONS-NCI_NATURE PATHWAYCOMMONS-REACTOME Wu-Stein-2010

Physical interactions:
Abu-Odeh-Aqeilan-2014 Agrawal-Sedivy-2010 Aichem-Groettrup-2012 Albers-Koegl-2005 Alexandru-Deshaies-2008 Altun-Kessler-2011 Andresen-Flores-Morales-2014 Arbuckle-Grant-2010 Arora-Mercola-2008 BIOGRID-SMALL-SCALE-STUDIES Bandyopadhyay-Ideker-2010 Bantscheff-Drewes-2011 Barr-Knapp-2009 Barrios-Rodiles-Wrana-2005 Behrends-Harper-2010 Behzadnia-Lührmann-2007 Bennett-Harper-2010 Benzinger-Hermeking-2005 Berggård-James-2006 Bett-Hay-2013 Blandin-Richard-2013 Bouwmeester-Superti-Furga-2004 Brajenovic-Drewes-2004 Brehme-Superti-Furga-2009 Bruderer-Hay-2011 Byron-Humphries-2012 Cai-Conaway-2007 Camargo-Brandon-2007 Cannavo-Jiricny-2007 Cao-Chinnaiyan-2014 Chen-Ge-2013 Chen-Naus-2012 Chen-Zhang-2013 Cheng-Chen-2010 Cloutier-Coulombe-2013 Colland-Gauthier-2004 Couzens-Gingras-2013 Cox-Rizzino-2013 Danielsen-Nielsen-2011 Dyer-Sobral-2010 Emanuele-Elledge-2011 Ewing-Figeys-2007 Fenner-Prehn-2010 Foerster-Ritter-2013 Foster-Marshall-2013 Freibaum-Taylor-2010 Gao-Reinberg-2012 Gautier-Hall-2009 Giannone-Liu-2010 Glatter-Gstaiger-2009 Gloeckner-Ueffing-2007 Goehler-Wanker-2004 Golebiowski-Hay-2009 Goudreault-Gingras-2009 Grant-2010 Greco-Cristea-2011 Havrylov-Redowicz-2009 Havugimana-Emili-2012 Hayes-Urbé-2012 Hegele-Stelzl-2012 A Hegele-Stelzl-2012 B Humphries-Humphries-2009 Hutchins-Peters-2010 IREF-BIND IREF-BIOGRID IREF-DIP IREF-HPRD IREF-INNATEDB IREF-INTACT IREF-MATRIXDB IREF-MPPI IREF-OPHID IREF-PUBMED IREF-SMALL-SCALE-STUDIES Ingham-Pawson-2005 Jeronimo-Coulombe-2007 Jin-Pawson-2004 Jones-MacBeath-2006 Joshi-Cristea-2013 Jäger-Krogan-2012 Kahle-Zoghbi-2011 Kim-Gygi-2011 Kneissl-Grummt-2003 Koch-Hermeking-2007 Kristensen-Foster-2012 Lau-Ronai-2012 Lee-Doedens-2011 Lee-Songyang-2011 Lehner-Sanderson-2004 A Lehner-Sanderson-2004 B Leng-Wang-2014 Li-Dorf-2011 A Li-Dorf-2011 B Lim-Zoghbi-2006 Liu-Wang-2012 Loch-Strickler-2012 Lopitz-Otsoa-Rodriguez-2012 Lu-Zhang-2013 Mak-Moffat-2010 Malovannaya-Qin-2010 Markson-Sanderson-2009 Maréchal-Zou-2014 Matafora-Bachi-2009 Matsumoto-Nakayama-2005 McCracken-Blencowe-2005 McFarland-Nussbaum-2008 Meek-Piwnica-Worms-2004 Meierhofer-Kaiser-2008 Miyamoto-Sato-Yanagawa-2010 Nakayama-Ohara-2002 Nakayasu-Adkins-2013 Napolitano-Meroni-2011 Nathan-Goldberg-2013 Neganova-Lako-2011 Newman-Keating-2003 Olma-Pintard-2009 Oláh-Ovádi-2011 Oshikawa-Nakayama-2012 Ouyang-Gill-2009 Panigrahi-Pati-2012 Perez-Hernandez-Yáñez-Mó-2013 Persaud-Rotin-2009 Pichlmair-Superti-Furga-2011 Pichlmair-Superti-Furga-2012 Pilot-Storck-Goillot-2010 Povlsen-Choudhary-2012 Ramachandran-LaBaer-2004 Ravasi-Hayashizaki-2010 Reinke-Keating-2013 Richter-Chrzanowska-Lightowlers-2010 Roux-Burke-2012 Rowbotham-Mermoud-2011 Roy-Parent-2013 Rual-Vidal-2005 A Rual-Vidal-2005 B San-Marina-Minden-2008 Sang-Jackson-2011 Sato-Conaway-2004 Shi-Qin-2011 Singh-Moore-2012 Soler-López-Aloy-2011 Sowa-Harper-2009 Stehling-Lill-2012 Stelzl-Wanker-2005 Suter-Wanker-2013 Taipale-Lindquist-2012 Takahashi-Conaway-2011 Tarallo-Weisz-2011 Tatham-Hay-2011 Teixeira-Gomes-2010 Thalappilly-Dusetti-2008 Tsai-Cristea-2012 Udeshi-Carr-2012 Vandamme-Angrand-2011 Vanderwerf-Bagby-2009 Varjosalo-Gstaiger-2013 A Varjosalo-Gstaiger-2013 B Varjosalo-Superti-Furga-2013 Venkatesan-Vidal-2009 Vinayagam-Wanker-2011 Wagner-Choudhary-2011 Wallach-Kramer-2013 Wang-Balch-2006 Wang-He-2008 Wang-Yang-2011 Weimann-Stelzl-2013 A Weimann-Stelzl-2013 B Weinmann-Meister-2009 Wilker-Yaffe-2007 Wong-O'Bryan-2012 Woods-Monteiro-2012 Woodsmith-Sanderson-2012 Wu-Li-2007 Wu-Ma-2012 Xiao-Lefkowitz-2007

Xie-Cong-2013 Xu-Jaffrey-2010 Xu-Ye-2012 Yang-Chen-2010 Yatim-Benkirane-2012 Yu-Chow-2013 Yu-Vidal-2011 Zanon-Pichler-2013 Zhang-Zou-2011 Zhao-Krug-2005 Zhao-Yang-2011 Zhou-Conrads-2004 Zhou-Liang-2012 de Hoog-Mann-2004 van Wijk-Timmers-2009

Predicted:
I2D-BIND-Fly2Human I2D-BIND-Mouse2Human I2D-BIND-Rat2Human I2D-BIND-Worm2Human I2D-BIND-Yeast2Human I2D-BioGRID-Fly2Human I2D-BioGRID-Mouse2Human I2D-BioGRID-Rat2Human I2D-BioGRID-Worm2Human I2D-BioGRID-Yeast2Human I2D-Chen-Pawson-2009-PiwiScreen-Mouse2Human I2D-Formstecher-Daviet-2005-Embryo-Fly2Human I2D-Giot-Rothbert-2003-Low-Fly2Human I2D-INNATEDB-Mouse2Human I2D-IntAct-Fly2Human I2D-IntAct-Mouse2Human I2D-IntAct-Rat2Human I2D-IntAct-Worm2Human I2D-IntAct-Yeast2Human I2D-Krogan-Greenblatt-2006-Core-Yeast2Human I2D-Krogan-Greenblatt-2006-NonCore-Yeast2Human I2D-Li-Vidal-2004-CORE-1-Worm2Human I2D-Li-Vidal-2004-non-core-Worm2Human I2D-MGI-Mouse2Human I2D-MINT-Fly2Human I2D-MINT-Mouse2Human I2D-MINT-Rat2Human I2D-MINT-Worm2Human I2D-MINT-Yeast2Human I2D-Manual-Mouse2Human I2D-Manual-Rat2Human I2D-Ptacek-Snyder-2005-Yeast2Human I2D-Tarassov-PCA-Yeast2Human I2D-Tewari-Vidal-2004-TGFb-Worm2Human I2D-Wang-Orkin-2006-EScmplx-Mouse2Human I2D-Wang-Orkin-2006-EScmplxlow-Mouse2Human I2D-Yu-Vidal-2008-GoldStd-Yeast2Human I2D-vonMering-Bork-2002-High-Yeast2Human I2D-vonMering-Bork-2002-Low-Yeast2Human I2D-vonMering-Bork-2002-Medium-Yeast2Human Stuart-Kim-2003

Shared protein domains:
INTERPRO PFAM

Network weighting: Automatically selected weighting method (Assigned based on query genes)

Number of gene results: 20

Networks

Co-expression 65.86 %

Mallon-McKay-2013 10.43 %

StemCellDB: the human pluripotent stem cell database at the National Institutes of Health. Mallon et al. (2013). *Stem Cell Res*.

Source: Pearson correlation with 567,140 interactions from GEO

Tags: cultured cells; stem cells; cell line

Chen-Brown-2002 10.03 %

Gene expression patterns in human liver cancers. Chen et al. (2002). *Mol Biol Cell*.

Source: Pearson correlation with 275,649 interactions from supplementary material

Tags: cell proliferation; transcription factors; liver; cancer

Wang-Maris-2006 9.70 %

Integrative genomics identifies distinct molecular classes of neuroblastoma and shows that multiple genes are targeted by regional alterations in DNA copy number. Wang et al. (2006). *Cancer Res*.

Source: Pearson correlation with 264,234 interactions from GEO

Tags: transcription factors; cancer

Bild-Nevins-2006 B 7.47 %

Oncogenic pathway signatures in human cancers as a guide to targeted therapies. Bild et al. (2006). *Nature*.

Note: One of 3 datasets produced from this publication.

Source: Pearson correlation with 282,582 interactions from GEO

Tags: cultured cells; signal transduction; cancer; epithelial cells; cell line; disease; breast; transcription factors; breast cancer

Salaverria-Siebert-2011 6.55 %

Translocations activating IRF4 identify a subtype of germinal center-derived B-cell lymphoma affecting predominantly children and young adults. Salaverria et al. (2011). *Blood*.

Source: Pearson correlation with 514,070 interactions from GEO

Tags: transcription factors; cancer

Ramaswamy-Golub-2001 6.24 %

Multiclass cancer diagnosis using tumor gene expression signatures. Ramaswamy et al. (2001). *Proc Natl Acad Sci U S A*.

Source: Pearson correlation with 270,142 interactions from supplementary material

Tags: cancer

Arijs-Rutgeerts-2009 4.45 %

Mucosal gene expression of antimicrobial peptides in inflammatory bowel disease before and after first infliximab treatment. Arijs et al. (2009). *PLoS One*.

Source: Pearson correlation with 653,194 interactions from GEO

Tags: immune system

Alizadeh-Staudt-2000 4.04 %

Distinct types of diffuse large B-cell lymphoma identified by gene expression profiling. Alizadeh et al. (2000). *Nature*.

Source: Pearson correlation with 88,888 interactions from supplementary material

Tags: cultured cells; cancer

Rieger-Chu-2004 3.04 %

Toxicity from radiation therapy associated with abnormal transcriptional responses to DNA damage. Rieger et al. (2004). *Proc Natl Acad Sci U S A*.

Source: Pearson correlation with 259,055 interactions from GEO

Tags: cultured cells; cell line

Roth-Zlotnik-2006 **2.47 %**

Gene expression analyses reveal molecular relationships among 20 regions of the human CNS. Roth et al. (2006). *Neurogenetics*.

Source: Pearson correlation with 666,614 interactions from GEO

Boldrick-Relman-2002 **1.45 %**

Stereotyped and specific gene expression programs in human innate immune responses to bacteria. Boldrick et al. (2002). *Proc Natl Acad Sci U S A*.

Source: Pearson correlation with 108,543 interactions from supplementary material

Tags: immune system

Shared protein domains **24.06 %**

INTERPRO **12.35 %**

Source: Pearson correlation with 562,560 interactions from InterPro

PFAM **11.71 %**

Source: Pearson correlation with 543,464 interactions from Pfam

Physical interactions **7.64 %**

IREF-BIOGRID **6.91 %**

Source: Direct interaction with 127,207 interactions from iRefIndex

IREF-SMALL-SCALE-STUDIES **0.73 %**

Source: Direct interaction with 69,119 interactions from iRefIndex

Pathway **2.11 %**

Wu-Stein-2010 **2.11 %**

A human functional protein interaction network and its application to cancer data analysis. Wu et al. (2010). *Genome Biol*.

Source: 78,183 interactions from supplementary material

Tags: transcription factors; cancer

Genetic interactions **0.33 %**

Lin-Smith-2010 **0.33 %**

A genome-wide map of human genetic interactions inferred from radiation hybrid genotypes. Lin et al. (2010). *Genome Res*.

Source: 4,836,794 interactions from supplementary material

Attributes

Attribute	Gene

Genes

MET (P08581) met proto-oncogene [Source:HGNC Symbol;Acc:7029]

Functions:
1

Synonyms: ENSG00000105976; ENSP00000317272; ENSP00000380860; ENSP00000398140; ENSP00000398776; ENSP00000410980; ENSP00000413857; ENSP00000445020; 4233; MET; NP_000236; NP_001120972; NM_000245; NM_001127500; HGFR; RCCP2; MET_HUMAN; P08581;

More at Entrez

CYP11A1 (P05108) cytochrome P450, family 11, subfamily A, polypeptide 1 [Source:HGNC Symbol;Acc:2590]

Functions:
1 8 10

Synonyms: ENSG00000140459; ENSP00000268053; ENSP00000351455; ENSP00000388018; ENSP00000391081; ENSP00000402064; ENSP00000405488; ENSP00000439750; ENSP00000456598; ENSP00000456941; 1583; CYP11A1; NP_000772; NP_001093243; NM_000781; NM_001099773; CYP11A; P450SCC; CP11A_HUMAN; P05108;

More at Entrez

MMP12 (P39900) matrix metallopeptidase 12 (macrophage elastase) [Source:HGNC Symbol;Acc:7158]

Functions:
1 4 6 7 9

Synonyms: ENSG00000262406; ENSP00000458585; 4321; MMP12; NP_002417; NM_002426; HME; MMP12_HUMAN; P39900;

More at Entrez

CA2 (P00918) carbonic anhydrase II [Source:HGNC Symbol;Acc:1373]

Functions:
1 2 3

Synonyms: ENSG00000104267; ENSP00000285379; ENSP00000428443; ENSP00000428947; 760; CA2; NP_000058; NM_000067; CA-II; CAII; Car2; CAH2_HUMAN; P00918;

More at Entrez

CA9 (Q16790) carbonic anhydrase IX [Source:HGNC Symbol;Acc:1383]

Functions:
1 2 3

Synonyms: ENSG00000107159; ENSP00000367608; 768; CA9; NP_001207; NM_001216; CAIX; CAH9_HUMAN; Q16790;

More at Entrez

EPHX2 (P34913) epoxide hydrolase 2, cytoplasmic [Source:HGNC Symbol;Acc:3402]

Functions:
1 8 10

Synonyms: ENSG00000120915; ENSP00000369843; ENSP00000427956; ENSP00000428191; ENSP00000428875; ENSP00000430269; ENSP00000430302; ENSP00000430779; 2053; EPHX2; NP_001243411; NP_001243412; NP_001243413; NP_001970; NM_001256482; NM_001256483; NM_001256484; NM_001979; HYES_HUMAN; P34913;

More at Entrez

MIF (P14174) macrophage migration inhibitory factor (glycosylation-inhibiting factor) [Source:HGNC Symbol;Acc:7097]

Functions:
1 5

Synonyms: ENSG00000240972; ENSP00000215754; 4282; MIF; NP_002406; NM_002415; GLIF; MIF_HUMAN; P14174;

More at Entrez

PPARG (P37231) peroxisome proliferator-activated receptor gamma [Source:HGNC Symbol;Acc:9236]

Functions:

1 3

Synonyms: ENSG00000132170; ENSP00000287820; ENSP00000312472; ENSP00000380195; ENSP00000380196; ENSP00000380205; ENSP00000380207; ENSP00000380210; ENSP00000380218; ENSP00000380221; ENSP00000380224; ENSP00000392285; ENSP00000411931; ENSP00000438940; 5468; PPARG; NP_005028; NP_056953; NP_619725; NP_619726; NM_005037; NM_015869; NM_138711; NM_138712; NR1C3; PPARG1; PPARG2; PPARgamma; P37231; PPARG_HUMAN;

More at Entrez

KIF11 (P52732) kinesin family member 11 [Source:HGNC Symbol;Acc:6388]

Functions:

1

Synonyms: ENSG00000138160; ENSP00000260731; 3832; KIF11; NP_004514; NM_004523; Eg5; HKSP; KNSL1; TRIP5; KIF11_HUMAN; P52732;

More at Entrez

MAPK14 (Q16539) mitogen-activated protein kinase 14 [Source:HGNC Symbol;Acc:6876]

Functions:

1

Synonyms: ENSG00000112062; ENSP00000229794; ENSP00000229795; ENSP00000308669; ENSP00000417065; ENSP00000417531; ENSP00000419141; ENSP00000419837; 1432; MAPK14; NP_001306; NP_620581; NP_620582; NP_620583; NM_001315; NM_139012; NM_139014; CSBP1; CSBP2; CSPB1; Mxi2; PRKM14; PRKM15; MK14_HUMAN; Q16539;

More at Entrez

MMP9 (P14780) matrix metallopeptidase 9 (gelatinase B, 92kDa gelatinase, 92kDa type IV collagenase) [Source:HGN

Functions:

1 4 6 7 9

Synonyms: ENSG00000100985; ENSP00000361405; 4318; MMP9; NP_004985; NM_004994; CLG4B; MMP9_HUMAN; P14780;

More at Entrez

DDTL D-dopachrome tautomerase-like [Source:HGNC Symbol;Acc:33446] 5.25

Functions:

Synonyms: ENSG00000099974; ENSP00000215770; 100037417; DDTL; NP_001077862; NM_001084393; A6NHG4; DDTL_HUMAN;

More at Entrez

DDT D-dopachrome tautomerase [Source:HGNC Symbol;Acc:2732] 4.13

Functions:

5

Synonyms: ENSG00000099977; ENSP00000215773; ENSP00000381386; ENSP00000384866; ENSP00000385714; ENSP00000399768; ENSP00000409440; 1652; DDT; NP_001077861; NP_001346; NM_001084392; NM_001355; DDCT; DOPD_HUMAN; P30046;

More at Entrez

CA13 carbonic anhydrase XIII [Source:HGNC Symbol;Acc:14914] 1.61

Functions:

2 3

Synonyms: ENSG00000185015; ENSP00000318912; 100507258; 377677; CA13; NP_940986; NM_198584; CAXIII; FLJ37995; MGC59868; CAH13_HUMAN; Q8N1Q1;

More at Entrez

CA5B carbonic anhydrase VB, mitochondrial [Source:HGNC Symbol;Acc:1378] 1.41

Functions:

2 3

Synonyms: ENSG00000169239; ENSP00000314099; ENSP00000417021; ENSP00000417236; ENSP00000417553; ENSP00000418622; 11238; CA5B; NP_009151; NM_007220; CAH5B_HUMAN; Q9Y2D0;

More at Entrez

CA10 carbonic anhydrase X [Source:HGNC Symbol;Acc:1369] 1.40

Functions:

Synonyms: ENSG00000154975; ENSP00000285273; ENSP00000340363; ENSP00000390666; ENSP00000405388; ENSP00000458145; ENSP00000459619; ENSP00000460238; ENSP00000461908; 56934; CA10; NP_001076002; NP_001076003; NP_064563; NM_001082533; NM_001082534; NM_020178; CA-RPX; CARPX; HUCEP-15; CAH10_HUMAN; Q9NS85;

More at Entrez

CA8 carbonic anhydrase VIII [Source:HGNC Symbol;Acc:1382] 1.38

Functions:

Synonyms: ENSG00000178538; ENSP00000314407; 767; CA8; NP_004047; NM_004056; CALS; CAH8_HUMAN; P35219;

More at Entrez

CA14 carbonic anhydrase XIV [Source:HGNC Symbol;Acc:1372] 1.33

Functions:

2 3

Synonyms: ENSG00000118298; ENSG00000264654; ENSP00000358107; ENSP00000462389; ENSP00000475238; ENSP00000475869; 23632; CA14; NP_036245; NM_012113; CAH14_HUMAN; Q9ULX7;

More at Entrez

FDX1 ferredoxin 1 [Source:HGNC Symbol;Acc:3638] 1.31

Functions:

8 10

Synonyms: ENSG00000137714; ENSP00000260270; 2230; FDX1; NP_004100; NM_004109; ADX; FDX; ADX_HUMAN; P10109;

More at Entrez

CXCL5 chemokine (C-X-C motif) ligand 5 [Source:HGNC Symbol;Acc:10642] 1.28

Functions:

Synonyms: ENSG00000163735; ENSP00000296027; 6374; CXCL5; NP_002985; NM_002994; ENA-78; SCYB5; CXCL5_HUMAN; P42830;

More at Entrez

CA6 carbonic anhydrase VI [Source:HGNC Symbol;Acc:1380] 1.28

Functions:

2 3 11

Synonyms: ENSG00000131686; ENSP00000366654; ENSP00000366661; ENSP00000366662; ENSP00000435280; ENSP00000447108; 765; CA6; NP_001206; NP_001257429; NP_001257430; NP_001257431; NM_001215; NM_001270500; NM_001270501; CAH6_HUMAN; P23280;

More at Entrez

CA12 carbonic anhydrase XII [Source:HGNC Symbol;Acc:1371] 1.28

Functions:

| 2 | 3 | | | | | | | | |

Synonyms: ENSG00000074410; ENSP00000178638; ENSP00000343088; ENSP00000403028; 771; CA12; NP_001209; NP_996808; NM_001218; NM_206925; HsT18816; CAH12_HUMAN; O43570;

More at Entrez

CXCL6 chemokine (C-X-C motif) ligand 6 [Source:HGNC Symbol;Acc:10643] 1.23

Functions:

Synonyms: ENSG00000124875; ENSP00000226317; ENSP00000424819; 6372; CXCL6; NP_002984; NM_002993; CKA-3; GCP-2; SCYB6; CXCL6_HUMAN; P80162;

More at Entrez

CA1 carbonic anhydrase I [Source:HGNC Symbol;Acc:1368] 1.22

Functions: 2 3

Synonyms: ENSG00000133742; ENSP00000256119; ENSP00000392338; ENSP00000401551; ENSP00000427773; ENSP00000427852; ENSP00000428923; ENSP00000429300; ENSP00000429688; ENSP00000429798; ENSP00000429843; ENSP00000430372; ENSP00000430471; ENSP00000430543; ENSP00000430571; ENSP00000430656; ENSP00000430710; ENSP00000430737; ENSP00000430861; ENSP00000430975; ENSP00000443517; 759; CA1; NP_001122301; NP_001122302; NP_001122303; NP_001158302; NP_001729; NM_001128829; NM_001128830; NM_001128831; NM_001164830; NM_001738; CAH1_HUMAN; P00915;

More at Entrez

CA4 carbonic anhydrase IV [Source:HGNC Symbol;Acc:1375] 1.22

Functions: 2 3 11

Synonyms: ENSG00000167434; ENSP00000300900; ENSP00000464757; ENSP00000465837; ENSP00000466964; ENSP00000467465; 762; CA4; NP_000708; NM_000717; CAIV; Car4; RP17; CAH4_HUMAN; P22748;

More at Entrez

CA3 carbonic anhydrase III, muscle specific [Source:HGNC Symbol;Acc:1374] 1.21

Functions: 2 3

Synonyms: ENSG00000164879; ENSP00000285381; ENSP00000429760; 761; CA3; NP_005172; NM_005181; CAIII; Car3; CAH3_HUMAN; P07451;

More at Entrez

CA7 carbonic anhydrase VII [Source:HGNC Symbol;Acc:1381] 1.20

Functions: 2 3

Synonyms: ENSG00000168748; ENSP00000345659; ENSP00000377632; ENSP00000447178; 766; CA7; NP_001014435; NP_005173; NM_001014435; NM_005182; CAH7_HUMAN; P43166;

More at Entrez

CA5A carbonic anhydrase VA, mitochondrial [Source:HGNC Symbol;Acc:1377] 1.16

Functions: 2 3

Synonyms: ENSG00000174990; ENSP00000309649; 763; CA5A; NP_001730; NM_001739; CA5; CAVA; CAH5A_HUMAN; P35218;

More at Entrez

CA11 carbonic anhydrase XI [Source:HGNC Symbol;Acc:1370] 1.14

Functions:

Synonyms: ENSG00000063180; ENSP00000084798; ENSP00000469234; 770; CA11; NP_001208; NM_001217; CARP2; CARPX1; CAH11_HUMAN; O75493;

More at Entrez

MMP1	matrix metallopeptidase 1 (interstitial collagenase) [Source:HGNC Symbol;Acc:7155]	1.07

Functions:

4 6 7 9

Synonyms: ENSG00000196611; ENSP00000322788; 4312; MMP1; NP_001139410; NP_002412; NM_001145938; NM_002421; MMP1_HUMAN; P03956;

More at Entrez

SLC4A8	solute carrier family 4, sodium bicarbonate cotransporter, member 8 [Source:HGNC Symbol;Acc:11034]	1.05

Functions:

2 3

Synonyms: ENSG00000050438; ENSP00000351483; ENSP00000378325; ENSP00000405812; ENSP00000441520; ENSP00000442561; 9498; SLC4A8; NP_001035049; NP_001245330; NP_001245331; NP_001245332; NP_001254544; NM_001039960; NM_001258401; NM_001258402; NM_001258403; NM_001267615; Q2Y0W8; S4A8_HUMAN;

More at Entrez

Functions

Function	FDR	Coverage
1 query genes	n/a	11 / 11
2 bicarbonate transport	3.19E-26	13 / 29
3 organic anion transport	6.75E-15	14 / 254
4 metalloendopeptidase activity	6.86E-2	3 / 27
5 intramolecular oxidoreductase activity, transposing C=C bonds	5.37E-1	2 / 10
6 multicellular organismal catabolic process	5.48E-1	3 / 71
7 collagen catabolic process	5.48E-1	3 / 65
8 cholesterol metabolic process	5.48E-1	3 / 69
9 metallopeptidase activity	6.07E-1	3 / 80
10 sterol metabolic process	6.07E-1	3 / 80
11 hydro-lyase activity	6.14E-1	2 / 17

Interactions

Gene 1	Gene 2	Weight	Network group	Networks
MMP1	MMP12	0.10282072611153126	Co-expression	Arijs-Rutgeerts-2009<br>Ramaswamy-Golub-2001<br>Wang-Maris-2006
MMP1	CXCL5	0.06496808491647243	Co-expression	Arijs-Rutgeerts-2009<br>Roth-Zlotnik-2006<br>Salaverria-Siebert-2011
MMP9	MMP12	0.059718070551753044	Co-expression	Arijs-Rutgeerts-2009<br>Chen-Brown-2002<br>Wang-Maris-2006
MMP1	MMP9	0.05919322092086077	Co-expression	Alizadeh-Staudt-2000<br>Arijs-Rutgeerts-2009<br>Chen-Brown-2002<br>Wang-Maris-2006
CXCL6	CXCL5	0.05336379539221525	Co-expression	Arijs-Rutgeerts-2009<br>Salaverria-Siebert-2011<br>Wang-Maris-2006
DDT	MIF	0.051807248033583164	Co-expression	Bild-Nevins-2006 B<br>Salaverria-Siebert-2011<br>Wang-Maris-2006
CA6	CA14	0.024239005520939827	Co-expression	Ramaswamy-Golub-2001
MMP9	CA2	0.02351631224155426	Co-expression	Bild-Nevins-2006 B
CA9	CA2	0.021494185552001	Co-expression	Salaverria-Siebert-2011
MMP1	CA12	0.020530197769403458	Co-expression	Salaverria-Siebert-2011
CA12	MMP12	0.020372465252876282	Co-expression	Chen-Brown-2002
FDX1	CYP11A1	0.01983389165252447	Co-expression	Mallon-McKay-2013<br>Roth-Zlotnik-2006
MAPK14	KIF11	0.01903084572404623	Co-expression	Chen-Brown-2002<br>Roth-Zlotnik-2006
CXCL5	MMP9	0.01895942911505699	Co-expression	Boldrick-Relman-2002
CA9	MET	0.01891346648335457	Co-expression	Ramaswamy-Golub-2001
CA13	EPHX2	0.01882065087556839	Co-expression	Mallon-McKay-2013
EPHX2	CA2	0.01672244258224964	Co-expression	Mallon-McKay-2013
CA12	CXCL5	0.016581028699874878	Co-expression	Wang-Maris-2006
CXCL6	CA12	0.016061080619692802	Co-expression	Wang-Maris-2006
MMP1	MET	0.015004313550889492	Co-expression	Salaverria-Siebert-2011
CA5A	CA6	0.013614140450954437	Co-expression	Ramaswamy-Golub-2001
CXCL6	MMP12	0.01343515794724226	Co-expression	Mallon-McKay-2013
PPARG	CA2	0.01244678907096386	Co-expression	Arijs-Rutgeerts-2009<br>Rieger-Chu-2004
MMP9	CYP11A1	0.011989451013505459	Co-expression	Mallon-McKay-2013
CA5B	CYP11A1	0.010530075058341026	Co-expression	Mallon-McKay-2013
MMP1	CA9	0.010340913198888302	Co-expression	Roth-Zlotnik-2006
FDX1	EPHX2	0.009916039183735847	Co-expression	Arijs-Rutgeerts-2009
FDX1	CA2	0.009201055392622948	Co-expression	Chen-Brown-2002
MIF	MMP12	0.009009147994220257	Co-expression	Alizadeh-Staudt-2000<br>Boldrick-Relman-2002
MMP1	CXCL6	0.008916885824874043	Co-expression	Arijs-Rutgeerts-2009<br>Boldrick-Relman-2002
CA11	CA14	0.008083850145339966	Co-expression	Mallon-McKay-2013
CA7	CA1	0.007495645899325609	Co-expression	Arijs-Rutgeerts-2009
MMP12	MET	0.0065786163322627544	Co-expression	Alizadeh-Staudt-2000
CXCL5	MET	0.006402609869837761	Co-expression	Bild-Nevins-2006 B
DDT	EPHX2	0.006105517968535423	Co-expression	Roth-Zlotnik-2006
CA12	PPARG	0.0056481570936739445	Co-expression	Arijs-Rutgeerts-2009
CA12	CA2	0.004792694002389908	Co-expression	Arijs-Rutgeerts-2009
CA1	CXCL5	0.004494811873883009	Co-expression	Bild-Nevins-2006 B

CA7	CXCL5	0.0037256747018545866	Co-expression	Bild-Nevins-2006 B
CA4	CA2	0.0037032561376690865	Co-expression	Arijs-Rutgeerts-2009
CA7	CA2	0.0033373881597071886	Co-expression	Arijs-Rutgeerts-2009
CA1	CA2	0.003328366205096245	Co-expression	Arijs-Rutgeerts-2009
CA5B	EPHX2	0.011935488320887089	Genetic interactions	Lin-Smith-2010
CA13	EPHX2	0.0029491730965673923	Genetic interactions	Lin-Smith-2010
CXCL6	EPHX2	0.0016794513212516904	Genetic interactions	Lin-Smith-2010
CA13	MAPK14	0.001627411344088614	Genetic interactions	Lin-Smith-2010
KIF11	CA2	0.0015893412055447698	Genetic interactions	Lin-Smith-2010
PPARG	CYP11A1	9.334263158962131E-4	Genetic interactions	Lin-Smith-2010
FDX1	CA10	8.189075160771608E-4	Genetic interactions	Lin-Smith-2010
SLC4A8	CA8	7.704430026933551E-4	Genetic interactions	Lin-Smith-2010
MAPK14	EPHX2	7.498431950807571E-4	Genetic interactions	Lin-Smith-2010
CA12	CA8	6.630378193221986E-4	Genetic interactions	Lin-Smith-2010
CA12	PPARG	6.485359044745564E-4	Genetic interactions	Lin-Smith-2010
CA8	KIF11	6.437188130803406E-4	Genetic interactions	Lin-Smith-2010
DDT	PPARG	6.367801688611507E-4	Genetic interactions	Lin-Smith-2010
CA10	CYP11A1	6.214058375917375E-4	Genetic interactions	Lin-Smith-2010
CA12	MAPK14	6.055634003132582E-4	Genetic interactions	Lin-Smith-2010
SLC4A8	CA10	5.016854265704751E-4	Genetic interactions	Lin-Smith-2010
CA8	PPARG	4.8519985284656286E-4	Genetic interactions	Lin-Smith-2010
CA8	CA10	3.230099973734468E-4	Genetic interactions	Lin-Smith-2010
CA10	PPARG	3.159451298415661E-4	Genetic interactions	Lin-Smith-2010
CA10	MAPK14	2.9501033714041114E-4	Genetic interactions	Lin-Smith-2010
MMP1	PPARG	0.018458472564816475	Pathway	Wu-Stein-2010
MMP9	PPARG	0.012255456298589706	Pathway	Wu-Stein-2010
FDX1	CYP11A1	0.7656212449073792	Physical interactions	IREF-BIOGRID<br>IREF-SMALL-SCALE-STUDIES
SLC4A8	CA2	0.6753623783588409	Physical interactions	IREF-BIOGRID<br>IREF-SMALL-SCALE-STUDIES
CXCL6	MMP9	0.6474498212337494	Physical interactions	IREF-BIOGRID<br>IREF-SMALL-SCALE-STUDIES
CXCL5	MMP9	0.61274204403162	Physical interactions	IREF-BIOGRID<br>IREF-SMALL-SCALE-STUDIES
MMP1	CXCL6	0.11795808374881744	Physical interactions	IREF-SMALL-SCALE-STUDIES
CA9	CA2	0.10896948724985123	Physical interactions	IREF-BIOGRID<br>IREF-SMALL-SCALE-STUDIES
MMP1	CXCL5	0.06495930254459381	Physical interactions	IREF-SMALL-SCALE-STUDIES
DDTL	MIF	0.9624011516571045	Shared protein domains	INTERPRO<br>PFAM
DDT	MIF	0.9624011516571045	Shared protein domains	INTERPRO<br>PFAM
DDT	DDTL	0.9624011516571045	Shared protein domains	INTERPRO<br>PFAM
CA9	CA2	0.13466692715883255	Shared protein domains	INTERPRO<br>PFAM
CA13	CA2	0.13466692715883255	Shared protein domains	INTERPRO<br>PFAM
CA13	CA9	0.13466692715883255	Shared protein domains	INTERPRO<br>PFAM
CA5B	CA2	0.13466692715883255	Shared protein domains	INTERPRO<br>PFAM
CA5B	CA9	0.13466692715883255	Shared protein domains	INTERPRO<br>PFAM
CA5B	CA13	0.13466692715883255	Shared protein domains	INTERPRO<br>PFAM
CA10	CA2	0.13466692715883255	Shared protein domains	INTERPRO<br>PFAM
CA10	CA9	0.13466692715883255	Shared protein domains	INTERPRO<br>PFAM

CA10	CA13	0.13466692715883255	Shared protein domains	INTERPRO<br>PFAM
CA10	CA5B	0.13466692715883255	Shared protein domains	INTERPRO<br>PFAM
CA8	CA2	0.13466692715883255	Shared protein domains	INTERPRO<br>PFAM
CA8	CA9	0.13466692715883255	Shared protein domains	INTERPRO<br>PFAM
CA8	CA13	0.13466692715883255	Shared protein domains	INTERPRO<br>PFAM
CA8	CA5B	0.13466692715883255	Shared protein domains	INTERPRO<br>PFAM
CA8	CA10	0.13466692715883255	Shared protein domains	INTERPRO<br>PFAM
CA14	CA2	0.13466692715883255	Shared protein domains	INTERPRO<br>PFAM
CA14	CA9	0.13466692715883255	Shared protein domains	INTERPRO<br>PFAM
CA14	CA13	0.13466692715883255	Shared protein domains	INTERPRO<br>PFAM
CA14	CA5B	0.13466692715883255	Shared protein domains	INTERPRO<br>PFAM
CA14	CA10	0.13466692715883255	Shared protein domains	INTERPRO<br>PFAM
CA14	CA8	0.13466692715883255	Shared protein domains	INTERPRO<br>PFAM
CA6	CA2	0.13466692715883255	Shared protein domains	INTERPRO<br>PFAM
CA6	CA9	0.13466692715883255	Shared protein domains	INTERPRO<br>PFAM
CA6	CA13	0.13466692715883255	Shared protein domains	INTERPRO<br>PFAM
CA6	CA5B	0.13466692715883255	Shared protein domains	INTERPRO<br>PFAM
CA6	CA10	0.13466692715883255	Shared protein domains	INTERPRO<br>PFAM
CA6	CA8	0.13466692715883255	Shared protein domains	INTERPRO<br>PFAM
CA6	CA14	0.13466692715883255	Shared protein domains	INTERPRO<br>PFAM
CA12	CA2	0.13466692715883255	Shared protein domains	INTERPRO<br>PFAM
CA12	CA9	0.13466692715883255	Shared protein domains	INTERPRO<br>PFAM
CA12	CA13	0.13466692715883255	Shared protein domains	INTERPRO<br>PFAM
CA12	CA5B	0.13466692715883255	Shared protein domains	INTERPRO<br>PFAM
CA12	CA10	0.13466692715883255	Shared protein domains	INTERPRO<br>PFAM
CA12	CA8	0.13466692715883255	Shared protein domains	INTERPRO<br>PFAM
CA12	CA14	0.13466692715883255	Shared protein domains	INTERPRO<br>PFAM
CA12	CA6	0.13466692715883255	Shared protein domains	INTERPRO<br>PFAM
CA1	CA2	0.13466692715883255	Shared protein domains	INTERPRO<br>PFAM
CA1	CA9	0.13466692715883255	Shared protein domains	INTERPRO<br>PFAM
CA1	CA13	0.13466692715883255	Shared protein domains	INTERPRO<br>PFAM
CA1	CA5B	0.13466692715883255	Shared protein domains	INTERPRO

				PFAM
CA1	CA10	0.13466692715883255	Shared protein domains	INTERPRO PFAM
CA1	CA8	0.13466692715883255	Shared protein domains	INTERPRO PFAM
CA1	CA14	0.13466692715883255	Shared protein domains	INTERPRO PFAM
CA1	CA6	0.13466692715883255	Shared protein domains	INTERPRO PFAM
CA1	CA12	0.13466692715883255	Shared protein domains	INTERPRO PFAM
CA4	CA2	0.13466692715883255	Shared protein domains	INTERPRO PFAM
CA4	CA9	0.13466692715883255	Shared protein domains	INTERPRO PFAM
CA4	CA13	0.13466692715883255	Shared protein domains	INTERPRO PFAM
CA4	CA5B	0.13466692715883255	Shared protein domains	INTERPRO PFAM
CA4	CA10	0.13466692715883255	Shared protein domains	INTERPRO PFAM
CA4	CA8	0.13466692715883255	Shared protein domains	INTERPRO PFAM
CA4	CA14	0.13466692715883255	Shared protein domains	INTERPRO PFAM
CA4	CA6	0.13466692715883255	Shared protein domains	INTERPRO PFAM
CA4	CA12	0.13466692715883255	Shared protein domains	INTERPRO PFAM
CA4	CA1	0.13466692715883255	Shared protein domains	INTERPRO PFAM
CA3	CA2	0.13466692715883255	Shared protein domains	INTERPRO PFAM
CA3	CA9	0.13466692715883255	Shared protein domains	INTERPRO PFAM
CA3	CA13	0.13466692715883255	Shared protein domains	INTERPRO PFAM
CA3	CA5B	0.13466692715883255	Shared protein domains	INTERPRO PFAM
CA3	CA10	0.13466692715883255	Shared protein domains	INTERPRO PFAM
CA3	CA8	0.13466692715883255	Shared protein domains	INTERPRO PFAM
CA3	CA14	0.13466692715883255	Shared protein domains	INTERPRO PFAM
CA3	CA6	0.13466692715883255	Shared protein domains	INTERPRO PFAM
CA3	CA12	0.13466692715883255	Shared protein domains	INTERPRO PFAM
CA3	CA1	0.13466692715883255	Shared protein domains	INTERPRO PFAM
CA3	CA4	0.13466692715883255	Shared protein domains	INTERPRO PFAM
CA7	CA2	0.13466692715883255	Shared protein domains	INTERPRO PFAM
CA7	CA9	0.13466692715883255	Shared protein domains	INTERPRO PFAM
CA7	CA13	0.13466692715883255	Shared protein domains	INTERPRO PFAM
CA7	CA5B	0.13466692715883255	Shared protein domains	INTERPRO PFAM
CA7	CA10	0.13466692715883255	Shared protein domains	INTERPRO

				PFAM
CA7	CA8	0.13466692715883255	Shared protein domains	INTERPRO PFAM
CA7	CA14	0.13466692715883255	Shared protein domains	INTERPRO PFAM
CA7	CA6	0.13466692715883255	Shared protein domains	INTERPRO PFAM
CA7	CA12	0.13466692715883255	Shared protein domains	INTERPRO PFAM
CA7	CA1	0.13466692715883255	Shared protein domains	INTERPRO PFAM
CA7	CA4	0.13466692715883255	Shared protein domains	INTERPRO PFAM
CA7	CA3	0.13466692715883255	Shared protein domains	INTERPRO PFAM
CA5A	CA2	0.13466692715883255	Shared protein domains	INTERPRO PFAM
CA5A	CA9	0.13466692715883255	Shared protein domains	INTERPRO PFAM
CA5A	CA13	0.13466692715883255	Shared protein domains	INTERPRO PFAM
CA5A	CA5B	0.13466692715883255	Shared protein domains	INTERPRO PFAM
CA5A	CA10	0.13466692715883255	Shared protein domains	INTERPRO PFAM
CA5A	CA8	0.13466692715883255	Shared protein domains	INTERPRO PFAM
CA5A	CA14	0.13466692715883255	Shared protein domains	INTERPRO PFAM
CA5A	CA6	0.13466692715883255	Shared protein domains	INTERPRO PFAM
CA5A	CA12	0.13466692715883255	Shared protein domains	INTERPRO PFAM
CA5A	CA1	0.13466692715883255	Shared protein domains	INTERPRO PFAM
CA5A	CA4	0.13466692715883255	Shared protein domains	INTERPRO PFAM
CA5A	CA3	0.13466692715883255	Shared protein domains	INTERPRO PFAM
CA5A	CA7	0.13466692715883255	Shared protein domains	INTERPRO PFAM
CA11	CA2	0.13466692715883255	Shared protein domains	INTERPRO PFAM
CA11	CA9	0.13466692715883255	Shared protein domains	INTERPRO PFAM
CA11	CA13	0.13466692715883255	Shared protein domains	INTERPRO PFAM
CA11	CA5B	0.13466692715883255	Shared protein domains	INTERPRO PFAM
CA11	CA10	0.13466692715883255	Shared protein domains	INTERPRO PFAM
CA11	CA8	0.13466692715883255	Shared protein domains	INTERPRO PFAM
CA11	CA14	0.13466692715883255	Shared protein domains	INTERPRO PFAM
CA11	CA6	0.13466692715883255	Shared protein domains	INTERPRO PFAM
CA11	CA12	0.13466692715883255	Shared protein domains	INTERPRO PFAM
CA11	CA1	0.13466692715883255	Shared protein domains	INTERPRO PFAM
CA11	CA4	0.13466692715883255	Shared protein domains	INTERPRO

				PFAM
CA11	CA3	0.13466692715883255	Shared protein domains	INTERPRO PFAM
CA11	CA7	0.13466692715883255	Shared protein domains	INTERPRO PFAM
CA11	CA5A	0.13466692715883255	Shared protein domains	INTERPRO PFAM
MMP1	MMP12	0.08611704409122467	Shared protein domains	INTERPRO PFAM
MMP9	MMP12	0.06935350596904755	Shared protein domains	INTERPRO PFAM
MMP1	MMP9	0.06908712908625603	Shared protein domains	INTERPRO PFAM
CXCL6	CXCL5	0.05678102932870388	Shared protein domains	INTERPRO PFAM

Search results generated by the GeneMANIA algorithm (genemania.org)

GENEMANIA

Created on: 23 July 2015 10:20:21
Last database update: 1 June 2014 20:00:00
Application version: 3.1.2.8

Report of GeneMANIA search

Network image

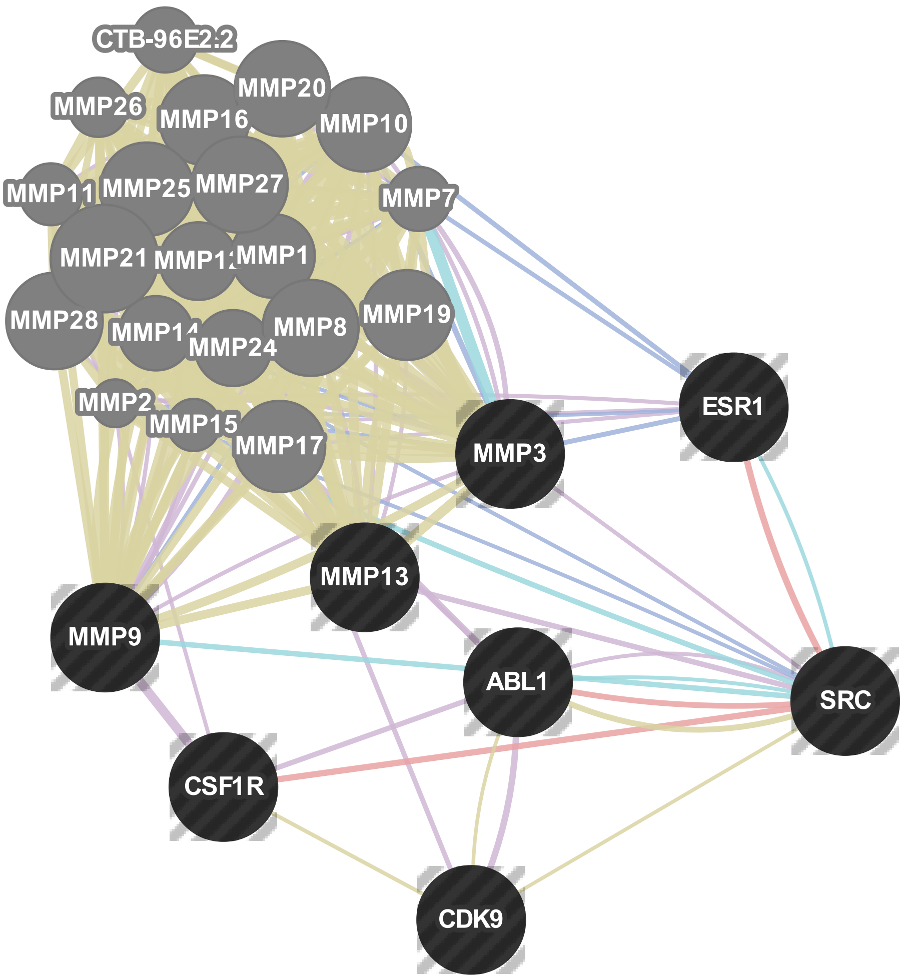

Functions legend

- query genes

Networks legend

- Co-expression
- Co-localization
- Pathway
- Physical interactions
- Shared protein domains

Search parameters

Organism: H. sapiens (human)

Genes: P03372; P00519; P14780; P50750; P45452; P08254; P07333; P12931

Networks:

Attributes:

Co-expression:
Alizadeh-Staudt-2000 Arijs-Rutgeerts-2009 Bahr-Bowler-2013 Bild-Nevins-2006 B
Boldrick-Relman-2002 Burington-Shaughnessy-2008 Chen-Brown-2002 Cheok-Evans-2003
Gysin-McMahon-2012 Innocenti-Brown-2011 Kang-Willman-2010 Mallon-McKay-2013
Perou-Botstein-1999 Ramaswamy-Golub-2001 Rieger-Chu-2004 Roth-Zlotnik-2006
Salaverria-Siebert-2011 Smirnov-Cheung-2009 Wang-Maris-2006 Wu-Garvey-2007

Co-localization:
Johnson-Shoemaker-2003 Satoh-Yamamoto-2013 Schadt-Shoemaker-2004

Genetic interactions:
BIOGRID-SMALL-SCALE-STUDIES IREF-SMALL-SCALE-STUDIES Lin-Boeke-2012 A Lin-Boeke-2012 B
Lin-Smith-2010 Toyoshima-Grandori-2012 Willingham-Muchowski-2003

Pathway:
PATHWAYCOMMONS-CELL_MAP PATHWAYCOMMONS-HUMANCYC PATHWAYCOMMONS-IMID
PATHWAYCOMMONS-NCI_NATURE PATHWAYCOMMONS-REACTOME Wu-Stein-2010

Physical interactions:
Abu-Odeh-Aqeilan-2014 Agrawal-Sedivy-2010 Aichem-Groettrup-2012 Albers-Koegl-2005
Alexandru-Deshaies-2008 Altun-Kessler-2011 Andresen-Flores-Morales-2014 Arbuckle-Grant-2010
Arora-Mercola-2008 BIOGRID-SMALL-SCALE-STUDIES Bandyopadhyay-Ideker-2010
Bantscheff-Drewes-2011 Barr-Knapp-2009 Barrios-Rodiles-Wrana-2005 Behrends-Harper-2010
Behzadnia-Lührmann-2007 Bennett-Harper-2010 Benzinger-Hermeking-2005 Berggård-James-2006
Bett-Hay-2013 Blandin-Richard-2013 Bouwmeester-Superti-Furga-2004 Brajenovic-Drewes-2004
Brehme-Superti-Furga-2009 Bruderer-Hay-2011 Byron-Humphries-2012 Cai-Conaway-2007
Camargo-Brandon-2007 Cannavo-Jiricny-2007 Cao-Chinnaiyan-2014 Chen-Ge-2013 Chen-Naus-2012
Chen-Zhang-2013 Cheng-Chen-2010 Cloutier-Coulombe-2013 Colland-Gauthier-2004
Couzens-Gingras-2013 Cox-Rizzino-2013 Danielsen-Nielsen-2011 Dyer-Sobral-2010
Emanuele-Elledge-2011 Ewing-Figeys-2007 Fenner-Prehn-2010 Foerster-Ritter-2013
Foster-Marshall-2013 Freibaum-Taylor-2010 Gao-Reinberg-2012 Gautier-Hall-2009 Giannone-Liu-2010
Glatter-Gstaiger-2009 Gloeckner-Ueffing-2007 Goehler-Wanker-2004 Golebiowski-Hay-2009
Goudreault-Gingras-2009 Grant-2010 Greco-Cristea-2011 Havrylov-Redowicz-2009
Havugimana-Emili-2012 Hayes-Urbé-2012 Hegele-Stelzl-2012 A Hegele-Stelzl-2012 B
Humphries-Humphries-2009 Hutchins-Peters-2010 IREF-BIND IREF-BIOGRID IREF-DIP IREF-HPRD
IREF-INNATEDB IREF-INTACT IREF-MATRIXDB IREF-MPPI IREF-OPHID IREF-PUBMED
IREF-SMALL-SCALE-STUDIES Ingham-Pawson-2005 Jeronimo-Coulombe-2007 Jin-Pawson-2004
Jones-MacBeath-2006 Joshi-Cristea-2013 Jäger-Krogan-2012 Kahle-Zoghbi-2011 Kim-Gygi-2011
Kneissl-Grummt-2003 Koch-Hermeking-2007 Kristensen-Foster-2012 Lau-Ronai-2012
Lee-Doedens-2011 Lee-Songyang-2011 Lehner-Sanderson-2004 A Lehner-Sanderson-2004 B
Leng-Wang-2014 Li-Dorf-2011 A Li-Dorf-2011 B Lim-Zoghbi-2006 Liu-Wang-2012
Loch-Strickler-2012 Lopitz-Otsoa-Rodriguez-2012 Lu-Zhang-2013 Mak-Moffat-2010
Malovannaya-Qin-2010 Markson-Sanderson-2009 Maréchal-Zou-2014 Matafora-Bachi-2009
Matsumoto-Nakayama-2005 McCracken-Blencowe-2005 McFarland-Nussbaum-2008
Meek-Piwnica-Worms-2004 Meierhofer-Kaiser-2008 Miyamoto-Sato-Yanagawa-2010
Nakayama-Ohara-2002 Nakayasu-Adkins-2013 Napolitano-Meroni-2011 Nathan-Goldberg-2013
Neganova-Lako-2011 Newman-Keating-2003 Olma-Pintard-2009 Oláh-Ovádi-2011
Oshikawa-Nakayama-2012 Ouyang-Gill-2009 Panigrahi-Pati-2012 Perez-Hernandez-Yáñez-Mó-2013
Persaud-Rotin-2009 Pichlmair-Superti-Furga-2011 Pichlmair-Superti-Furga-2012 Pilot-Storck-Goillot-2010
Povlsen-Choudhary-2012 Ramachandran-LaBaer-2004 Ravasi-Hayashizaki-2010 Reinke-Keating-2013
Richter-Chrzanowska-Lightowlers-2010 Roux-Burke-2012 Rowbotham-Mermoud-2011 Roy-Parent-2013
Rual-Vidal-2005 A Rual-Vidal-2005 B San-Marina-Minden-2008 Sang-Jackson-2011
Sato-Conaway-2004 Shi-Qin-2011 Singh-Moore-2012 Soler-López-Aloy-2011 Sowa-Harper-2009
Stehling-Lill-2012 Stelzl-Wanker-2005 Suter-Wanker-2013 Taipale-Lindquist-2012
Takahashi-Conaway-2011 Tarallo-Weisz-2011 Tatham-Hay-2011 Teixeira-Gomes-2010
Thalappilly-Dusetti-2008 Tsai-Cristea-2012 Udeshi-Carr-2012 Vandamme-Angrand-2011
Vanderwerf-Bagby-2009 Varjosalo-Gstaiger-2013 A Varjosalo-Gstaiger-2013 B
Varjosalo-Superti-Furga-2013 Venkatesan-Vidal-2009 Vinayagam-Wanker-2011 Wagner-Choudhary-2011
Wallach-Kramer-2013 Wang-Balch-2006 Wang-He-2008 Wang-Yang-2011 Weimann-Stelzl-2013 A
Weimann-Stelzl-2013 B Weinmann-Meister-2009 Wilker-Yaffe-2007 Wong-O'Bryan-2012
Woods-Monteiro-2012 Woodsmith-Sanderson-2012 Wu-Li-2007 Wu-Ma-2012 Xiao-Lefkowitz-2007

Xie-Cong-2013 Xu-Jaffrey-2010 Xu-Ye-2012 Yang-Chen-2010 Yatim-Benkirane-2012 Yu-Chow-2013 Yu-Vidal-2011 Zanon-Pichler-2013 Zhang-Zou-2011 Zhao-Krug-2005 Zhao-Yang-2011 Zhou-Conrads-2004 Zhou-Liang-2012 de Hoog-Mann-2004 van Wijk-Timmers-2009

Predicted:
I2D-BIND-Fly2Human I2D-BIND-Mouse2Human I2D-BIND-Rat2Human I2D-BIND-Worm2Human I2D-BIND-Yeast2Human I2D-BioGRID-Fly2Human I2D-BioGRID-Mouse2Human I2D-BioGRID-Rat2Human I2D-BioGRID-Worm2Human I2D-BioGRID-Yeast2Human I2D-Chen-Pawson-2009-PiwiScreen-Mouse2Human I2D-Formstecher-Daviet-2005-Embryo-Fly2Human I2D-Giot-Rothbert-2003-Low-Fly2Human I2D-INNATEDB-Mouse2Human I2D-IntAct-Fly2Human I2D-IntAct-Mouse2Human I2D-IntAct-Rat2Human I2D-IntAct-Worm2Human I2D-IntAct-Yeast2Human I2D-Krogan-Greenblatt-2006-Core-Yeast2Human I2D-Krogan-Greenblatt-2006-NonCore-Yeast2Human I2D-Li-Vidal-2004-CORE-1-Worm2Human I2D-Li-Vidal-2004-non-core-Worm2Human I2D-MGI-Mouse2Human I2D-MINT-Fly2Human I2D-MINT-Mouse2Human I2D-MINT-Rat2Human I2D-MINT-Worm2Human I2D-MINT-Yeast2Human I2D-Manual-Mouse2Human I2D-Manual-Rat2Human I2D-Ptacek-Snyder-2005-Yeast2Human I2D-Tarassov-PCA-Yeast2Human I2D-Tewari-Vidal-2004-TGFb-Worm2Human I2D-Wang-Orkin-2006-EScmplx-Mouse2Human I2D-Wang-Orkin-2006-EScmplxlow-Mouse2Human I2D-Yu-Vidal-2008-GoldStd-Yeast2Human I2D-vonMering-Bork-2002-High-Yeast2Human I2D-vonMering-Bork-2002-Low-Yeast2Human I2D-vonMering-Bork-2002-Medium-Yeast2Human Stuart-Kim-2003

Shared protein domains:
INTERPRO PFAM

Network weighting: Automatically selected weighting method (Assigned based on query genes)

Number of gene results: 20

Networks

Shared protein domains **47.52 %**

PFAM **25.45 %**

Source: Pearson correlation with 543,464 interactions from Pfam

INTERPRO **22.07 %**

Source: Pearson correlation with 562,560 interactions from InterPro

Co-expression **41.88 %**

Burington-Shaughnessy-2008 **6.05 %**

Tumor cell gene expression changes following short-term in vivo exposure to single agent chemotherapeutics are related to survival in multiple myeloma. Burington et al. (2008). *Clin Cancer Res*.

Source: Pearson correlation with 293,587 interactions from GEO

Tags: transcription factors; time series; cancer; chemotherapy

Bahr-Bowler-2013 **5.73 %**

Peripheral blood mononuclear cell gene expression in chronic obstructive pulmonary disease. Bahr et al. (2013). *Am J Respir Cell Mol Biol*.

Source: Pearson correlation with 278,447 interactions from GEO

Alizadeh-Staudt-2000 **5.46 %**

Distinct types of diffuse large B-cell lymphoma identified by gene expression profiling. Alizadeh et al. (2000). *Nature*.

Source: Pearson correlation with 88,888 interactions from supplementary material

Tags: cultured cells; cancer

Bild-Nevins-2006 B **5.08 %**

Oncogenic pathway signatures in human cancers as a guide to targeted therapies. Bild et al. (2006). *Nature*.

Note: One of 3 datasets produced from this publication.

Source: Pearson correlation with 282,582 interactions from GEO

Tags: cultured cells; signal transduction; cancer; epithelial cells; cell line; disease; breast; transcription factors; breast cancer

Boldrick-Relman-2002 **4.96 %**

Stereotyped and specific gene expression programs in human innate immune responses to bacteria. Boldrick et al. (2002). *Proc Natl Acad Sci U S A*.

Source: Pearson correlation with 108,543 interactions from supplementary material

Tags: immune system

Arijs-Rutgeerts-2009 **4.05 %**

Mucosal gene expression of antimicrobial peptides in inflammatory bowel disease before and after first infliximab treatment. Arijs et al. (2009). *PLoS One*.

Source: Pearson correlation with 653,194 interactions from GEO

Tags: immune system

Ramaswamy-Golub-2001 **3.89 %**

Multiclass cancer diagnosis using tumor gene expression signatures. Ramaswamy et al. (2001). *Proc Natl Acad Sci U S A*.

Source: Pearson correlation with 270,142 interactions from supplementary material

Tags: cancer

Chen-Brown-2002 **2.86 %**

Gene expression patterns in human liver cancers. Chen et al. (2002). *Mol Biol Cell*.

Source: Pearson correlation with 275,649 interactions from supplementary material

Tags: cell proliferation; transcription factors; liver; cancer

Perou-Botstein-1999 **2.68 %**

Distinctive gene expression patterns in human mammary epithelial cells and breast cancers. Perou et al. (1999). *Proc Natl Acad Sci U S A*.

Source: Pearson correlation with 62,886 interactions from supplementary material

Tags: cultured cells; cancer; epithelial cells; signal transduction; breast; stromal cells; transcription factors; breast cancer

Smirnov-Cheung-2009 **1.11 %**

Genetic analysis of radiation-induced changes in human gene expression. Smirnov et al. (2009). *Nature*.

Source: Pearson correlation with 463,390 interactions from GEO

Tags: transcription factors; cultured cells; cell line

Co-localization **4.22 %**

Johnson-Shoemaker-2003 **4.22 %**

Genome-wide survey of human alternative pre-mRNA splicing with exon junction microarrays. Johnson et al. (2003). *Science*.

Source: Pearson correlation with 426,640 interactions from GEO

Tags: cultured cells; cell line

Physical interactions **4.00 %**

IREF-INTACT **1.52 %**

Source: Direct interaction with 42,475 interactions from iRefIndex

IREF-DIP **1.18 %**

Source: Direct interaction with 3,719 interactions from iRefIndex

IREF-BIND **0.93 %**

Source: Direct interaction with 7,078 interactions from iRefIndex

IREF-BIOGRID **0.37 %**

Source: Direct interaction with 127,207 interactions from iRefIndex

Pathway **2.38 %**

PATHWAYCOMMONS-NCI_NATURE **1.48 %**

Source: Direct interaction with 10,109 interactions from Pathway Commons

Wu-Stein-2010 **0.90 %**

A human functional protein interaction network and its application to cancer data analysis. Wu et al. (2010). *Genome Biol*.

Source: 78,183 interactions from supplementary material

Tags: transcription factors; cancer

Attributes

Attribute	Gene

Genes

MMP13 (P45452) matrix metallopeptidase 13 (collagenase 3) [Source:HGNC Symbol;Acc:7159]

Functions:

1 2 3 4 5 6 7 8 9

Synonyms: ENSG00000137745; ENSG00000262325; ENSP00000260302; ENSP00000339672; ENSP00000460695; ENSP00000461440; 4322; MMP13; NP_002418; NM_002427; CLG3; MMP13_HUMAN; P45452;

More at Entrez

SRC (P12931) v-src avian sarcoma (Schmidt-Ruppin A-2) viral oncogene homolog [Source:HGNC Symbol;Acc:11283]

Functions:

1

Synonyms: ENSG00000197122; ENSP00000350941; ENSP00000353950; ENSP00000362659; ENSP00000362668; ENSP00000362680; ENSP00000408503; 6714; SRC; NP_005408; NP_938033; NM_005417; NM_198291; ASV; c-src; P12931; SRC_HUMAN;

More at Entrez

MMP3 (P08254) matrix metallopeptidase 3 (stromelysin 1, progelatinase) [Source:HGNC Symbol;Acc:7173]

Functions:

1 2 3 4 5 6 7 8 9

Synonyms: ENSG00000149968; ENSG00000263313; ENSP00000299855; ENSP00000398346; ENSP00000435255; ENSP00000458496; ENSP00000459512; ENSP00000459712; 4314; MMP3; NP_002413; NM_002422; STMY; STMY1; MMP3_HUMAN; P08254;

More at Entrez

ESR1 (P03372) estrogen receptor 1 [Source:HGNC Symbol;Acc:3467]

Functions:

1

Synonyms: ENSG00000091831; ENSP00000206249; ENSP00000342630; ENSP00000384064; ENSP00000385373; ENSP00000387500; ENSP00000394721; ENSP00000401995; ENSP00000405330; ENSP00000411105; ENSP00000415934; 2099; ESR1; NP_000116; NP_001116212; NP_001116213; NP_001116214; NM_000125; NM_001122740; NM_001122741; NM_001122742; Era; ESR; NR3A1; ESR1_HUMAN; P03372;

More at Entrez

MMP9 (P14780) matrix metallopeptidase 9 (gelatinase B, 92kDa gelatinase, 92kDa type IV collagenase) [Source:HGN

Functions:

1 2 3 4 5 6 7 8 9 10 11 12

Synonyms: ENSG00000100985; ENSP00000361405; 4318; MMP9; NP_004985; NM_004994; CLG4B; MMP9_HUMAN; P14780;

More at Entrez

CSF1R (P07333) colony stimulating factor 1 receptor [Source:HGNC Symbol;Acc:2433]

Functions:

1

Synonyms: ENSG00000182578; ENSP00000286301; ENSP00000421174; ENSP00000422212; ENSP00000427545; ENSP00000445282; 1436; CSF1R; NP_005202; NM_005211; C-FMS; CD115; CSFR; FMS; CSF1R_HUMAN; P07333;

More at Entrez

CDK9 (P50750) cyclin-dependent kinase 9 [Source:HGNC Symbol;Acc:1780]

Functions:

1

Synonyms: ENSG00000136807; ENSP00000362361; ENSP00000362362; ENSP00000395872; 1025; CDK9; NP_001252; NM_001261; C-2k; CDC2L4; PITALRE; TAK; CDK9_HUMAN; P50750;

More at Entrez

ABL1 (P00519) c-abl oncogene 1, non-receptor tyrosine kinase [Source:HGNC Symbol;Acc:76]

Functions:
1

Synonyms: ENSG00000097007; ENSP00000323315; ENSP00000361423; ENSP00000376971; 25; ABL1; NP_005148; NP_009297; NM_005157; NM_007313; c-ABL; JTK7; ABL1_HUMAN; P00519;

More at Entrez

MMP21 matrix metallopeptidase 21 [Source:HGNC Symbol;Acc:14357] 2.80

Functions:

Synonyms: ENSG00000154485; ENSP00000357798; 118856; MMP21; NP_671724; NM_147191; MMP21_HUMAN; Q8N119;

More at Entrez

MMP28 matrix metallopeptidase 28 [Source:HGNC Symbol;Acc:14366] 2.67

Functions:
10 11 12 13 14

Synonyms: ENSG00000129270; ENSG00000271447; ENSP00000250144; ENSP00000464731; ENSP00000466155; ENSP00000466671; ENSP00000467034; ENSP00000468241; ENSP00000468249; ENSP00000468709; ENSP00000473853; 79148; MMP28; NP_001027449; NP_077278; NM_001032278; NM_024302; EPILYSIN; MM28; MMP-25; MMP-28; MMP28_HUMAN; Q9H239;

More at Entrez

MMP27 matrix metallopeptidase 27 [Source:HGNC Symbol;Acc:14250] 2.66

Functions:

Synonyms: ENSG00000137675; ENSP00000260229; 64066; MMP27; NP_071405; NM_022122; MMP27_HUMAN; Q9H306;

More at Entrez

MMP8 matrix metallopeptidase 8 (neutrophil collagenase) [Source:HGNC Symbol;Acc:7175] 2.66

Functions:
2 3 4 5 6 7 8 9 11

Synonyms: ENSG00000118113; ENSP00000236826; ENSP00000401004; ENSP00000431431; ENSP00000431938; ENSP00000433812; ENSP00000437173; 4317; MMP8; NP_002415; NM_002424; CLG1; MMP8_HUMAN; P22894;

More at Entrez

MMP20 matrix metallopeptidase 20 [Source:HGNC Symbol;Acc:7167] 2.66

Functions:
2 3 4 5 6 7 8 9

Synonyms: ENSG00000137674; ENSP00000260228; 9313; MMP20; NP_004762; NM_004771; MMP20_HUMAN; O60882;

More at Entrez

MMP10 matrix metallopeptidase 10 (stromelysin 2) [Source:HGNC Symbol;Acc:7156] 2.64

Functions:
2 3 4 5 6 7 8 9

Synonyms: ENSG00000166670; ENSP00000279441; ENSP00000441485; 4319; MMP10; NP_002416; NM_002425; STMY2; MMP10_HUMAN; P09238;

More at Entrez

MMP25 matrix metallopeptidase 25 [Source:HGNC Symbol;Acc:14246] 2.63

Functions:
13 14

Synonyms: ENSG00000008516; ENSP00000337816; 64386; MMP25; NP_071913; NM_022468; MMPL1; MT6-MMP; MMP25_HUMAN; Q9NPA2;

More at Entrez

MMP17 matrix metallopeptidase 17 (membrane-inserted) [Source:HGNC Symbol;Acc:7163] 2.61

Functions:

Synonyms: ENSG00000198598; ENSP00000353767; ENSP00000439542; ENSP00000441106; ENSP00000441710; ENSP00000442104; ENSP00000443727; ENSP00000444603; ENSP00000445620; 4326; MMP17; NP_057239; NM_016155; MT4-MMP; MMP17_HUMAN; Q9ULZ9;

More at Entrez

MMP16 matrix metallopeptidase 16 (membrane-inserted) [Source:HGNC Symbol;Acc:7162] 2.59

Functions:
4 8 9

Synonyms: ENSG00000156103; ENSP00000286614; ENSP00000429147; 4325; MMP16; NP_005932; NM_005941; C8orf57; DKFZp761D112; MT3-MMP; MMP16_HUMAN; P51512;

More at Entrez

MMP19 matrix metallopeptidase 19 [Source:HGNC Symbol;Acc:7165] 2.59

Functions:
2 3 4 5 6 7 8 9 13 14

Synonyms: ENSG00000123342; ENSP00000313437; ENSP00000377736; ENSP00000386625; ENSP00000446776; ENSP00000446979; ENSP00000447363; ENSP00000449752; 4327; MMP19; NP_001259030; NP_002420; NM_002429; MMP18; RASI-1; MMP19_HUMAN; Q99542;

More at Entrez

MMP1 matrix metallopeptidase 1 (interstitial collagenase) [Source:HGNC Symbol;Acc:7155] 2.52

Functions:
2 3 4 5 6 7 8 9 10 11 12

Synonyms: ENSG00000196611; ENSP00000322788; 4312; MMP1; NP_001139410; NP_002412; NM_001145938; NM_002421; MMP1_HUMAN; P03956;

More at Entrez

MMP12 matrix metallopeptidase 12 (macrophage elastase) [Source:HGNC Symbol;Acc:7158] 2.45

Functions:
2 3 4 5 6 7 8 9 10 11 12

Synonyms: ENSG00000262406; ENSP00000458585; 4321; MMP12; NP_002417; NM_002426; HME; MMP12_HUMAN; P39900;

More at Entrez

MMP24 matrix metallopeptidase 24 (membrane-inserted) [Source:HGNC Symbol;Acc:7172] 2.43

Functions:

Synonyms: ENSG00000125966; ENSP00000246186; 10893; MMP24; NP_006681; NM_006690; MT5-MMP; MMP24_HUMAN; Q9Y5R2;

More at Entrez

MMP14 matrix metallopeptidase 14 (membrane-inserted) [Source:HGNC Symbol;Acc:7160] 2.41

Functions:
2 3 4 5 6 7 8 9

Synonyms: ENSG00000157227; ENSP00000308208; ENSP00000446989; ENSP00000450323; 4323; MMP14; NP_004986; NM_004995; MT1-MMP; MMP14_HUMAN; P50281;

More at Entrez

CTB-96E2.2 Uncharacterized protein [Source:UniProtKB/TrEMBL;Acc:H0YJW9] 2.30

Functions:

Synonyms: ENSG00000262318; ENSG00000273171; ENSP00000452347; ENSP00000460756; CTB-96E2.2;

More at Ensembl

MMP7 matrix metallopeptidase 7 (matrilysin, uterine) [Source:HGNC Symbol;Acc:7174] 2.29

Functions:
2 3 4 5 6 7 8 9

Synonyms: ENSG00000137673; ENSP00000260227; 4316; MMP7; NP_002414; NM_002423; MPSL1; PUMP-1; MMP7_HUMAN; P09237;

More at Entrez

MMP11 matrix metallopeptidase 11 (stromelysin 3) [Source:HGNC Symbol;Acc:7157] 2.26

Functions:
2 3 4 5 6 7 8 9 13

Synonyms: ENSG00000099953; ENSP00000215743; ENSP00000408070; ENSP00000409860; ENSP00000412107; 4320; MMP11; NP_005931; NM_005940; STMY3; MMP11_HUMAN; P24347;

More at Entrez

MMP26 matrix metallopeptidase 26 [Source:HGNC Symbol;Acc:14249] 2.23

Functions:
13 14

Synonyms: ENSG00000167346; ENSP00000300762; ENSP00000369753; 56547; MMP26; NP_068573; NM_021801; endometase; MGC126590; MGC126592; MMP26_HUMAN; Q9NRE1;

More at Entrez

MMP15 matrix metallopeptidase 15 (membrane-inserted) [Source:HGNC Symbol;Acc:7161] 2.15

Functions:
2 3 4 5 6 7 8 9

Synonyms: ENSG00000102996; ENSP00000219271; ENSP00000457084; 4324; MMP15; NP_002419; NM_002428; MT2-MMP; MTMMP2; SMCP-2; MMP15_HUMAN; P51511;

More at Entrez

MMP2 matrix metallopeptidase 2 (gelatinase A, 72kDa gelatinase, 72kDa type IV collagenase) [Source:HGNC Sym 2.10

Functions:
2 3 4 5 6 7 8 9 11

Synonyms: ENSG00000087245; ENSP00000219070; ENSP00000394237; ENSP00000444143; ENSP00000456096; ENSP00000456518; ENSP00000457949; ENSP00000461421; ENSP00000461915; 4313; MMP2; NP_001121363; NP_004521; NM_001127891; NM_004530; CLG4; CLG4A; TBE-1; MMP2_HUMAN; P08253;

More at Entrez

Functions

Function	FDR	Coverage
1 query genes	n/a	8 / 8
2 collagen catabolic process	3.96E-24	14 / 65
3 multicellular organismal catabolic process	7.78E-24	14 / 71
4 extracellular matrix disassembly	6.3E-23	15 / 119
5 collagen metabolic process	7.26E-23	14 / 86
6 multicellular organismal macromolecule metabolic process	1.15E-22	14 / 90
7 multicellular organismal metabolic process	2.54E-22	14 / 96
8 extracellular matrix organization	2.46E-17	15 / 290
9 extracellular structure organization	2.46E-17	15 / 291
10 metalloendopeptidase activity	1.3E-4	4 / 27
11 endopeptidase activity	2.05E-4	6 / 163
12 metallopeptidase activity	9.09E-3	4 / 80
13 extracellular matrix	1.78E-2	5 / 208
14 proteinaceous extracellular matrix	2.72E-2	4 / 110

Interactions

Gene 1	Gene 2	Weight	Network group	Networks
MMP1	MMP3	0.08315662806853652	Co-expression	Arijs-Rutgeerts-2009<br>Bild-Nevins-2006 B<br>Boldrick-Relman-2002<br>Ramaswamy-Golub-2001
CSF1R	MMP9	0.07780082011595368	Co-expression	Alizadeh-Staudt-2000<br>Arijs-Rutgeerts-2009<br>Bild-Nevins-2006 B<br>Boldrick-Relman-2002<br>Ramaswamy-Golub-2001
MMP12	MMP1	0.06619915179908276	Co-expression	Arijs-Rutgeerts-2009<br>Ramaswamy-Golub-2001
MMP8	MMP9	0.048001520335674286	Co-expression	Arijs-Rutgeerts-2009<br>Burington-Shaughnessy-2008
MMP1	MMP10	0.046535199508070946	Co-expression	Arijs-Rutgeerts-2009<br>Ramaswamy-Golub-2001
MMP12	MMP3	0.043236750178039074	Co-expression	Arijs-Rutgeerts-2009<br>Boldrick-Relman-2002<br>Ramaswamy-Golub-2001
MMP17	ABL1	0.039785776287317276	Co-expression	Alizadeh-Staudt-2000<br>Boldrick-Relman-2002
ABL1	CDK9	0.039413267746567726	Co-expression	Alizadeh-Staudt-2000<br>Bahr-Bowler-2013
MMP1	MMP27	0.03739033732563257	Co-expression	Chen-Brown-2002<br>Smirnov-Cheung-2009
MMP1	MMP9	0.035868809558451176	Co-expression	Alizadeh-Staudt-2000<br>Arijs-Rutgeerts-2009<br>Chen-Brown-2002
MMP7	MMP12	0.03300734143704176	Co-expression	Bild-Nevins-2006 B<br>Ramaswamy-Golub-2001
MMP12	MMP9	0.03089035488665104	Co-expression	Arijs-Rutgeerts-2009<br>Chen-Brown-2002
SRC	MMP13	0.02658381685614586	Co-expression	Burington-Shaughnessy-2008
MMP7	MMP3	0.02531757391989231	Co-expression	Alizadeh-Staudt-2000
MMP10	MMP8	0.02511771209537983	Co-expression	Arijs-Rutgeerts-2009
ABL1	CSF1R	0.024075699038803577	Co-expression	Chen-Brown-2002<br>Perou-Botstein-1999
MMP14	MMP3	0.02110777236521244	Co-expression	Alizadeh-Staudt-2000
MMP8	MMP13	0.021025409922003746	Co-expression	Arijs-Rutgeerts-2009
MMP1	MMP8	0.020457519218325615	Co-expression	Arijs-Rutgeerts-2009
MMP17	CDK9	0.020025674253702164	Co-expression	Alizadeh-Staudt-2000
MMP12	MMP10	0.019088277593255043	Co-expression	Arijs-Rutgeerts-2009
MMP14	ABL1	0.018521782010793686	Co-expression	Bild-Nevins-2006 B
MMP12	MMP8	0.017592325806617737	Co-expression	Arijs-Rutgeerts-2009
MMP10	MMP9	0.016696784645318985	Co-expression	Arijs-Rutgeerts-2009
MMP14	MMP13	0.016678461804986	Co-expression	Perou-Botstein-1999
MMP10	MMP13	0.015926940366625786	Co-expression	Ramaswamy-Golub-2001
MMP11	MMP1	0.01591363735496998	Co-expression	Bild-Nevins-2006 B
MMP7	MMP10	0.015549114905297756	Co-expression	Arijs-Rutgeerts-2009
MMP11	MMP14	0.015414537861943245	Co-expression	Bild-Nevins-2006 B
MMP8	MMP3	0.01430840790271759	Co-expression	Arijs-Rutgeerts-2009
MMP11	MMP7	0.01417256984859705	Co-expression	Ramaswamy-Golub-2001
MMP10	MMP3	0.0137209203094244	Co-expression	Arijs-Rutgeerts-2009
MMP2	MMP7	0.013622893951833248	Co-expression	Perou-Botstein-1999
MMP2	MMP1	0.012175364419817924	Co-expression	Alizadeh-Staudt-2000
MMP15	MMP9	0.01169783086515963	Co-expression	Alizadeh-Staudt-2000<br>Smirnov-Cheung-2009
MMP11	MMP10	0.011331639252603054	Co-expression	Bild-Nevins-2006 B

MMP15	MMP28	0.01097777672111988	Co-expression	Smirnov-Cheung-2009
MMP2	MMP25	0.010518291965126991	Co-expression	Burington-Shaughnessy-2008
MMP2	MMP3	0.0100712226703763	Co-expression	Bild-Nevins-2006 B
MMP2	MMP11	0.009795740246772766	Co-expression	Bild-Nevins-2006 B
MMP19	MMP13	0.009640984237194061	Co-expression	Burington-Shaughnessy-2008
MMP17	ESR1	0.009530636481940746	Co-expression	Perou-Botstein-1999
MMP9	MMP3	0.009068215265870094	Co-expression	Arijs-Rutgeerts-2009
MMP2	MMP15	0.009007873013615608	Co-expression	Alizadeh-Staudt-2000
MMP14	MMP9	0.00881821196526289	Co-expression	Smirnov-Cheung-2009
MMP2	ESR1	0.008663809858262539	Co-expression	Alizadeh-Staudt-2000
MMP15	MMP17	0.008392536081373692	Co-expression	Perou-Botstein-1999
MMP25	SRC	0.008341304957866669	Co-expression	Bild-Nevins-2006 B
MMP15	MMP25	0.007599165663123131	Co-expression	Bild-Nevins-2006 B
MMP15	ESR1	0.006821854971349239	Co-expression	Perou-Botstein-1999
MMP2	CSF1R	0.0067098094150424	Co-expression	Alizadeh-Staudt-2000
MMP2	MMP9	0.006450654938817024	Co-expression	Alizadeh-Staudt-2000
MMP11	MMP20	0.006288273259997368	Co-expression	Burington-Shaughnessy-2008
ABL1	SRC	0.005723239853978157	Co-expression	Smirnov-Cheung-2009
MMP26	MMP27	0.0031631295569241047	Co-expression	Arijs-Rutgeerts-2009
MMP11	MMP24	0.002233868231996894	Co-expression	Burington-Shaughnessy-2008
MMP10	MMP3	0.02944069728255272	Co-localization	Johnson-Shoemaker-2003
MMP10	ESR1	0.02022220380604267	Co-localization	Johnson-Shoemaker-2003
ESR1	MMP3	0.01997765526175499	Co-localization	Johnson-Shoemaker-2003
MMP7	ESR1	0.014174806885421276	Co-localization	Johnson-Shoemaker-2003
MMP24	MMP16	0.012489370070397854	Co-localization	Johnson-Shoemaker-2003
MMP8	MMP9	0.01098998636007309	Co-localization	Johnson-Shoemaker-2003
MMP14	MMP19	0.009298608638346195	Co-localization	Johnson-Shoemaker-2003
MMP14	SRC	0.007836904376745224	Co-localization	Johnson-Shoemaker-2003
MMP14	MMP3	0.00761366356164217	Co-localization	Johnson-Shoemaker-2003
MMP2	SRC	0.00693303719162941	Co-localization	Johnson-Shoemaker-2003
MMP14	MMP10	0.00656958157196641	Co-localization	Johnson-Shoemaker-2003
MMP7	MMP14	0.0065179262310266495	Co-localization	Johnson-Shoemaker-2003
MMP2	MMP3	0.006387967616319656	Co-localization	Johnson-Shoemaker-2003
MMP2	MMP7	0.005242008715867996	Co-localization	Johnson-Shoemaker-2003
MMP2	ESR1	0.005118488799780607	Co-localization	Johnson-Shoemaker-2003
MMP2	MMP14	0.0035257197450846434	Co-localization	Johnson-Shoemaker-2003
MMP7	MMP3	0.10168006271123886	Pathway	Wu-Stein-2010
MMP2	MMP14	0.02908765897154808	Pathway	Wu-Stein-2010
MMP2	SRC	0.027302810922265053	Pathway	PATHWAYCOMMONS-NCI_NATURE<br>Wu-Stein-2010
MMP9	SRC	0.026412139181047678	Pathway	PATHWAYCOMMONS-NCI_NATURE<br>Wu-Stein-2010
ESR1	SRC	0.0069710928946733475	Pathway	PATHWAYCOMMONS-NCI_NATURE<br>Wu-Stein-2010
ABL1	SRC	0.006119976751506329	Pathway	Wu-Stein-2010
ESR1	SRC	0.0397075938526541	Physical interactions	IREF-BIOGRID<br>IREF-DIP<br>IREF-INTACT
CSF1R	SRC	0.03715650178492069	Physical interactions	IREF-BIOGRID<br>IREF-INTACT
ABL1	SRC	0.03036228520795703	Physical interactions	IREF-BIND<br>IREF-BIOGRID<br>IREF-INTACT
MMP24	MMP16	0.11205314472317696	Shared protein domains	INTERPRO<br>PFAM
MMP14	MMP16	0.11205314472317696	Shared protein domains	INTERPRO

				PFAM
MMP14	MMP24	0.11205314472317696	Shared protein domains	INTERPRO PFAM
MMP15	MMP16	0.10263661295175552	Shared protein domains	INTERPRO PFAM
MMP15	MMP24	0.10263661295175552	Shared protein domains	INTERPRO PFAM
MMP15	MMP14	0.10263661295175552	Shared protein domains	INTERPRO PFAM
MMP26	MMP7	0.09782937541604042	Shared protein domains	INTERPRO PFAM
MMP17	MMP20	0.08680696785449982	Shared protein domains	INTERPRO PFAM
MMP19	MMP20	0.08680696785449982	Shared protein domains	INTERPRO PFAM
MMP19	MMP17	0.08680696785449982	Shared protein domains	INTERPRO PFAM
MMP25	MMP20	0.08679590746760368	Shared protein domains	INTERPRO PFAM
MMP17	MMP25	0.08679590746760368	Shared protein domains	INTERPRO PFAM
MMP19	MMP25	0.08679590746760368	Shared protein domains	INTERPRO PFAM
MMP20	MMP13	0.08678418025374413	Shared protein domains	INTERPRO PFAM
MMP17	MMP13	0.08678418025374413	Shared protein domains	INTERPRO PFAM
MMP19	MMP13	0.08678418025374413	Shared protein domains	INTERPRO PFAM
MMP25	MMP13	0.08677312359213829	Shared protein domains	INTERPRO PFAM
MMP12	MMP20	0.08663327991962433	Shared protein domains	INTERPRO PFAM
MMP12	MMP17	0.08663327991962433	Shared protein domains	INTERPRO PFAM
MMP12	MMP19	0.08663327991962433	Shared protein domains	INTERPRO PFAM
MMP12	MMP25	0.08662226796150208	Shared protein domains	INTERPRO PFAM
MMP12	MMP13	0.0866105854511261	Shared protein domains	INTERPRO PFAM
MMP20	MMP27	0.08649903163313866	Shared protein domains	INTERPRO PFAM
MMP17	MMP27	0.08649903163313866	Shared protein domains	INTERPRO PFAM
MMP19	MMP27	0.08649903163313866	Shared protein domains	INTERPRO PFAM
MMP25	MMP27	0.08648804947733879	Shared protein domains	INTERPRO PFAM
MMP27	MMP13	0.0864764079451561	Shared protein domains	INTERPRO PFAM
MMP20	MMP21	0.08641837537288666	Shared protein domains	INTERPRO PFAM
MMP17	MMP21	0.08641837537288666	Shared protein domains	INTERPRO PFAM
MMP19	MMP21	0.08641837537288666	Shared protein domains	INTERPRO PFAM
MMP25	MMP21	0.08640741556882858	Shared protein domains	INTERPRO PFAM
MMP21	MMP13	0.08639579266309738	Shared protein domains	INTERPRO PFAM
MMP20	MMP3	0.08634748309850693	Shared protein domains	INTERPRO

				PFAM
MMP17	MMP3	0.08634748309850693	Shared protein domains	INTERPRO PFAM
MMP19	MMP3	0.08634748309850693	Shared protein domains	INTERPRO PFAM
MMP20	MMP8	0.08633934706449509	Shared protein domains	INTERPRO PFAM
MMP17	MMP8	0.08633934706449509	Shared protein domains	INTERPRO PFAM
MMP19	MMP8	0.08633934706449509	Shared protein domains	INTERPRO PFAM
MMP25	MMP3	0.08633654192090034	Shared protein domains	INTERPRO PFAM
MMP25	MMP8	0.0863284096121788	Shared protein domains	INTERPRO PFAM
MMP12	MMP27	0.08632660284638405	Shared protein domains	INTERPRO PFAM
MMP3	MMP13	0.08632494136691093	Shared protein domains	INTERPRO PFAM
MMP8	MMP13	0.08631680905818939	Shared protein domains	INTERPRO PFAM
MMP1	MMP20	0.08628861606121063	Shared protein domains	INTERPRO PFAM
MMP1	MMP17	0.08628861606121063	Shared protein domains	INTERPRO PFAM
MMP1	MMP19	0.08628861606121063	Shared protein domains	INTERPRO PFAM
MMP1	MMP25	0.08627768978476524	Shared protein domains	INTERPRO PFAM
MMP1	MMP13	0.08626610413193703	Shared protein domains	INTERPRO PFAM
MMP12	MMP21	0.08624627441167831	Shared protein domains	INTERPRO PFAM
MMP12	MMP3	0.08617567270994186	Shared protein domains	INTERPRO PFAM
MMP12	MMP8	0.08616757020354271	Shared protein domains	INTERPRO PFAM
MMP12	MMP1	0.08611704409122467	Shared protein domains	INTERPRO PFAM
MMP27	MMP21	0.08611324802041054	Shared protein domains	INTERPRO PFAM
MMP27	MMP3	0.086042869836092	Shared protein domains	INTERPRO PFAM
MMP8	MMP27	0.08603479340672493	Shared protein domains	INTERPRO PFAM
MMP1	MMP27	0.08598442748188972	Shared protein domains	INTERPRO PFAM
MMP21	MMP3	0.08596308156847954	Shared protein domains	INTERPRO PFAM
MMP8	MMP21	0.08595502376556396	Shared protein domains	INTERPRO PFAM
MMP1	MMP21	0.08590475097298622	Shared protein domains	INTERPRO PFAM
MMP8	MMP3	0.08588491007685661	Shared protein domains	INTERPRO PFAM
MMP1	MMP3	0.08583472296595573	Shared protein domains	INTERPRO PFAM
MMP1	MMP8	0.08582668751478195	Shared protein domains	INTERPRO PFAM
MMP10	MMP20	0.08571403473615646	Shared protein domains	INTERPRO PFAM
MMP17	MMP10	0.08571403473615646	Shared protein domains	INTERPRO

				PFAM
MMP19	MMP10	0.08571403473615646	Shared protein domains	INTERPRO PFAM
MMP25	MMP10	0.08570325374603271	Shared protein domains	INTERPRO PFAM
MMP10	MMP13	0.08569182828068733	Shared protein domains	INTERPRO PFAM
MMP12	MMP10	0.08554480597376823	Shared protein domains	INTERPRO PFAM
MMP10	MMP27	0.08541399985551834	Shared protein domains	INTERPRO PFAM
MMP10	MMP21	0.08533541485667229	Shared protein domains	INTERPRO PFAM
MMP10	MMP3	0.0852663442492485	Shared protein domains	INTERPRO PFAM
MMP10	MMP8	0.08525841683149338	Shared protein domains	INTERPRO PFAM
MMP1	MMP10	0.08520898595452309	Shared protein domains	INTERPRO PFAM
MMP16	MMP20	0.0835627056658268	Shared protein domains	INTERPRO PFAM
MMP16	MMP17	0.0835627056658268	Shared protein domains	INTERPRO PFAM
MMP16	MMP19	0.0835627056658268	Shared protein domains	INTERPRO PFAM
MMP24	MMP20	0.0835627056658268	Shared protein domains	INTERPRO PFAM
MMP24	MMP17	0.0835627056658268	Shared protein domains	INTERPRO PFAM
MMP24	MMP19	0.0835627056658268	Shared protein domains	INTERPRO PFAM
MMP14	MMP20	0.0835627056658268	Shared protein domains	INTERPRO PFAM
MMP14	MMP17	0.0835627056658268	Shared protein domains	INTERPRO PFAM
MMP14	MMP19	0.0835627056658268	Shared protein domains	INTERPRO PFAM
MMP16	MMP25	0.08355189487338066	Shared protein domains	INTERPRO PFAM
MMP24	MMP25	0.08355189487338066	Shared protein domains	INTERPRO PFAM
MMP14	MMP25	0.08355189487338066	Shared protein domains	INTERPRO PFAM
MMP16	MMP13	0.083540428429842	Shared protein domains	INTERPRO PFAM
MMP24	MMP13	0.083540428429842	Shared protein domains	INTERPRO PFAM
MMP14	MMP13	0.083540428429842	Shared protein domains	INTERPRO PFAM
MMP12	MMP16	0.0833929292857647	Shared protein domains	INTERPRO PFAM
MMP24	MMP12	0.0833929292857647	Shared protein domains	INTERPRO PFAM
MMP14	MMP12	0.0833929292857647	Shared protein domains	INTERPRO PFAM
MMP16	MMP27	0.08326169848442078	Shared protein domains	INTERPRO PFAM
MMP24	MMP27	0.08326169848442078	Shared protein domains	INTERPRO PFAM
MMP14	MMP27	0.08326169848442078	Shared protein domains	INTERPRO PFAM
MMP16	MMP21	0.08318285644054413	Shared protein domains	INTERPRO

				PFAM
MMP24	MMP21	0.08318285644054413	Shared protein domains	INTERPRO PFAM
MMP14	MMP21	0.08318285644054413	Shared protein domains	INTERPRO PFAM
MMP16	MMP3	0.08311356231570244	Shared protein domains	INTERPRO PFAM
MMP24	MMP3	0.08311356231570244	Shared protein domains	INTERPRO PFAM
MMP14	MMP3	0.08311356231570244	Shared protein domains	INTERPRO PFAM
MMP16	MMP8	0.08310561254620552	Shared protein domains	INTERPRO PFAM
MMP24	MMP8	0.08310561254620552	Shared protein domains	INTERPRO PFAM
MMP14	MMP8	0.08310561254620552	Shared protein domains	INTERPRO PFAM
MMP1	MMP16	0.0830560214817524	Shared protein domains	INTERPRO PFAM
MMP24	MMP1	0.0830560214817524	Shared protein domains	INTERPRO PFAM
MMP14	MMP1	0.0830560214817524	Shared protein domains	INTERPRO PFAM
MMP2	MMP9	0.08281752467155457	Shared protein domains	INTERPRO PFAM
MMP16	MMP10	0.08249437436461449	Shared protein domains	INTERPRO PFAM
MMP24	MMP10	0.08249437436461449	Shared protein domains	INTERPRO PFAM
MMP14	MMP10	0.08249437436461449	Shared protein domains	INTERPRO PFAM
MMP20	MMP28	0.08162881433963776	Shared protein domains	INTERPRO PFAM
MMP17	MMP28	0.08162881433963776	Shared protein domains	INTERPRO PFAM
MMP19	MMP28	0.08162881433963776	Shared protein domains	INTERPRO PFAM
MMP25	MMP28	0.08161871135234833	Shared protein domains	INTERPRO PFAM
MMP28	MMP13	0.08160799741744995	Shared protein domains	INTERPRO PFAM
MMP12	MMP28	0.08147017657756805	Shared protein domains	INTERPRO PFAM
MMP27	MMP28	0.08134755864739418	Shared protein domains	INTERPRO PFAM
MMP28	MMP21	0.0812738947570324	Shared protein domains	INTERPRO PFAM
MMP28	MMP3	0.08120914548635483	Shared protein domains	INTERPRO PFAM
MMP8	MMP28	0.0812017135322094	Shared protein domains	INTERPRO PFAM
MMP1	MMP28	0.08115537837147713	Shared protein domains	INTERPRO PFAM
MMP10	MMP28	0.0806305892765522	Shared protein domains	INTERPRO PFAM
MMP16	MMP28	0.07854435965418816	Shared protein domains	INTERPRO PFAM
MMP24	MMP28	0.07854435965418816	Shared protein domains	INTERPRO PFAM
MMP14	MMP28	0.07854435965418816	Shared protein domains	INTERPRO PFAM
MMP11	MMP20	0.07805588468909264	Shared protein domains	INTERPRO PFAM

MMP11	MMP17	0.07805588468909264	Shared protein domains	INTERPRO PFAM
MMP11	MMP19	0.07805588468909264	Shared protein domains	INTERPRO PFAM
MMP11	MMP25	0.07804539054632187	Shared protein domains	INTERPRO PFAM
MMP11	MMP13	0.07803426682949066	Shared protein domains	INTERPRO PFAM
MMP11	MMP12	0.07789112254977226	Shared protein domains	INTERPRO PFAM
MMP11	MMP27	0.07776376977562904	Shared protein domains	INTERPRO PFAM
MMP11	MMP21	0.07768725603818893	Shared protein domains	INTERPRO PFAM
MMP11	MMP3	0.0776200033724308	Shared protein domains	INTERPRO PFAM
MMP11	MMP8	0.07761228829622269	Shared protein domains	INTERPRO PFAM
MMP11	MMP1	0.07756416127085686	Shared protein domains	INTERPRO PFAM
MMP7	MMP20	0.07719079032540321	Shared protein domains	INTERPRO PFAM
MMP7	MMP17	0.07719079032540321	Shared protein domains	INTERPRO PFAM
MMP7	MMP19	0.07719079032540321	Shared protein domains	INTERPRO PFAM
MMP7	MMP25	0.07718152925372124	Shared protein domains	INTERPRO PFAM
MMP7	MMP13	0.0771716982126236	Shared protein domains	INTERPRO PFAM
MMP7	MMP12	0.07704530656337738	Shared protein domains	INTERPRO PFAM
MMP11	MMP10	0.077019102871418	Shared protein domains	INTERPRO PFAM
MMP7	MMP27	0.07693285122513771	Shared protein domains	INTERPRO PFAM
MMP7	MMP21	0.0768652856349945	Shared protein domains	INTERPRO PFAM
MMP7	MMP3	0.07680590450763702	Shared protein domains	INTERPRO PFAM
MMP7	MMP8	0.07679909467697144	Shared protein domains	INTERPRO PFAM
MMP7	MMP1	0.07675659656524658	Shared protein domains	INTERPRO PFAM
MMP15	MMP20	0.07667861878871918	Shared protein domains	INTERPRO PFAM
MMP15	MMP17	0.07667861878871918	Shared protein domains	INTERPRO PFAM
MMP15	MMP19	0.07667861878871918	Shared protein domains	INTERPRO PFAM
MMP15	MMP25	0.07666851952672005	Shared protein domains	INTERPRO PFAM
MMP15	MMP13	0.07665780559182167	Shared protein domains	INTERPRO PFAM
MMP15	MMP12	0.07651997357606888	Shared protein domains	INTERPRO PFAM
MMP15	MMP27	0.07639734819531441	Shared protein domains	INTERPRO PFAM
MMP15	MMP21	0.07632367685437202	Shared protein domains	INTERPRO PFAM
MMP7	MMP10	0.0762753002345562	Shared protein domains	INTERPRO PFAM

MMP15	MMP3	0.07625892385840416	Shared protein domains	INTERPRO PFAM
MMP15	MMP8	0.07625149190425873	Shared protein domains	INTERPRO PFAM
MMP15	MMP1	0.07620515301823616	Shared protein domains	INTERPRO PFAM
MMP15	MMP10	0.07568032294511795	Shared protein domains	INTERPRO PFAM
MMP11	MMP16	0.07519971951842308	Shared protein domains	INTERPRO PFAM
MMP11	MMP24	0.07519971951842308	Shared protein domains	INTERPRO PFAM
MMP11	MMP14	0.07519971951842308	Shared protein domains	INTERPRO PFAM
CTB-96E2.2	MMP28	0.0751018114387989	Shared protein domains	INTERPRO PFAM
MMP11	CTB-96E2.2	0.07482390105724335	Shared protein domains	INTERPRO PFAM
MMP7	MMP16	0.07424148172140121	Shared protein domains	INTERPRO PFAM
MMP7	MMP24	0.07424148172140121	Shared protein domains	INTERPRO PFAM
MMP7	MMP14	0.07424148172140121	Shared protein domains	INTERPRO PFAM
MMP15	MMP28	0.07203641533851624	Shared protein domains	INTERPRO PFAM
MMP26	MMP11	0.07163614593446255	Shared protein domains	INTERPRO PFAM
MMP20	MMP9	0.06948774307966232	Shared protein domains	INTERPRO PFAM
MMP17	MMP9	0.06948774307966232	Shared protein domains	INTERPRO PFAM
MMP19	MMP9	0.06948774307966232	Shared protein domains	INTERPRO PFAM
MMP2	MMP20	0.06948769092559814	Shared protein domains	INTERPRO PFAM
MMP2	MMP17	0.06948769092559814	Shared protein domains	INTERPRO PFAM
MMP2	MMP19	0.06948769092559814	Shared protein domains	INTERPRO PFAM
MMP25	MMP9	0.06947918981313705	Shared protein domains	INTERPRO PFAM
MMP2	MMP25	0.06947914138436317	Shared protein domains	INTERPRO PFAM
MMP9	MMP13	0.0694701261818409	Shared protein domains	INTERPRO PFAM
MMP2	MMP13	0.06947007402777672	Shared protein domains	INTERPRO PFAM
MMP12	MMP9	0.06935350596904755	Shared protein domains	INTERPRO PFAM
MMP2	MMP12	0.06935345754027367	Shared protein domains	INTERPRO PFAM
MMP27	MMP9	0.06924974918365479	Shared protein domains	INTERPRO PFAM
MMP2	MMP27	0.0692497007548809	Shared protein domains	INTERPRO PFAM
MMP21	MMP9	0.06918741390109062	Shared protein domains	INTERPRO PFAM
MMP2	MMP21	0.06918736174702644	Shared protein domains	INTERPRO PFAM
MMP9	MMP3	0.06913262233138084	Shared protein domains	INTERPRO PFAM

MMP2	MMP3	0.06913257390260696	Shared protein domains	INTERPRO PFAM
MMP8	MMP9	0.06912633776664734	Shared protein domains	INTERPRO PFAM
MMP2	MMP8	0.06912628933787346	Shared protein domains	INTERPRO PFAM
MMP1	MMP9	0.06908712908625603	Shared protein domains	INTERPRO PFAM
MMP2	MMP1	0.06908708065748215	Shared protein domains	INTERPRO PFAM
MMP15	MMP11	0.06907225027680397	Shared protein domains	INTERPRO PFAM
MMP10	MMP9	0.06864306330680847	Shared protein domains	INTERPRO PFAM
MMP2	MMP10	0.0686430111527443	Shared protein domains	INTERPRO PFAM
MMP26	MMP20	0.06816204264760017	Shared protein domains	INTERPRO PFAM
MMP26	MMP17	0.06816204264760017	Shared protein domains	INTERPRO PFAM
MMP26	MMP19	0.06816204264760017	Shared protein domains	INTERPRO PFAM
MMP26	MMP25	0.06815265864133835	Shared protein domains	INTERPRO PFAM
MMP26	MMP13	0.06814270839095116	Shared protein domains	INTERPRO PFAM
MMP15	MMP7	0.06805405765771866	Shared protein domains	INTERPRO PFAM
MMP26	MMP12	0.06801469251513481	Shared protein domains	INTERPRO PFAM
MMP26	MMP27	0.06790079548954964	Shared protein domains	INTERPRO PFAM
MMP26	MMP21	0.06783236563205719	Shared protein domains	INTERPRO PFAM
MMP26	MMP3	0.06777221709489822	Shared protein domains	INTERPRO PFAM
MMP26	MMP8	0.06776531785726547	Shared protein domains	INTERPRO PFAM
MMP26	MMP1	0.06772227585315704	Shared protein domains	INTERPRO PFAM
MMP26	MMP10	0.06723480299115181	Shared protein domains	INTERPRO PFAM
MMP28	MMP9	0.06704460456967354	Shared protein domains	INTERPRO PFAM
MMP2	MMP28	0.06704459711909294	Shared protein domains	INTERPRO PFAM
MMP2	MMP7	0.06695974990725517	Shared protein domains	INTERPRO PFAM
MMP7	MMP9	0.06695970147848129	Shared protein domains	INTERPRO PFAM
MMP11	MMP28	0.06691209971904755	Shared protein domains	INTERPRO PFAM
MMP16	MMP9	0.06685641780495644	Shared protein domains	INTERPRO PFAM
MMP24	MMP9	0.06685641780495644	Shared protein domains	INTERPRO PFAM
MMP14	MMP9	0.06685641780495644	Shared protein domains	INTERPRO PFAM
MMP2	MMP16	0.06685636937618256	Shared protein domains	INTERPRO PFAM
MMP2	MMP24	0.06685636937618256	Shared protein domains	INTERPRO PFAM

MMP2	MMP14	0.06685636937618256	Shared protein domains	INTERPRO PFAM
MMP26	MMP16	0.065691988915205	Shared protein domains	INTERPRO PFAM
MMP26	MMP24	0.065691988915205	Shared protein domains	INTERPRO PFAM
MMP26	MMP14	0.065691988915205	Shared protein domains	INTERPRO PFAM
MMP7	MMP28	0.06513834558427334	Shared protein domains	INTERPRO PFAM
CTB-96E2.2	MMP20	0.06170181185007095	Shared protein domains	INTERPRO PFAM
CTB-96E2.2	MMP17	0.06170181185007095	Shared protein domains	INTERPRO PFAM
CTB-96E2.2	MMP19	0.06170181185007095	Shared protein domains	INTERPRO PFAM
CTB-96E2.2	MMP25	0.061694083735346794	Shared protein domains	INTERPRO PFAM
CTB-96E2.2	MMP13	0.06168588809669018	Shared protein domains	INTERPRO PFAM
CTB-96E2.2	MMP12	0.06158047541975975	Shared protein domains	INTERPRO PFAM
CTB-96E2.2	MMP27	0.06148669123649597	Shared protein domains	INTERPRO PFAM
CTB-96E2.2	MMP21	0.061430344358086586	Shared protein domains	INTERPRO PFAM
MMP2	MMP11	0.06138291954994202	Shared protein domains	INTERPRO PFAM
MMP11	MMP9	0.06138289161026478	Shared protein domains	INTERPRO PFAM
CTB-96E2.2	MMP3	0.06138082034885883	Shared protein domains	INTERPRO PFAM
CTB-96E2.2	MMP8	0.06137513741850853	Shared protein domains	INTERPRO PFAM
CTB-96E2.2	MMP1	0.06133969500660896	Shared protein domains	INTERPRO PFAM
MMP15	MMP9	0.06131063774228096	Shared protein domains	INTERPRO PFAM
MMP2	MMP15	0.06131059117615223	Shared protein domains	INTERPRO PFAM
CTB-96E2.2	MMP10	0.06093830242753029	Shared protein domains	INTERPRO PFAM
MMP15	MMP26	0.060366034507751465	Shared protein domains	INTERPRO PFAM
MMP2	MMP26	0.05953052453696728	Shared protein domains	INTERPRO PFAM
MMP26	MMP9	0.059530315920710564	Shared protein domains	INTERPRO PFAM
CTB-96E2.2	MMP16	0.0593793373554945	Shared protein domains	INTERPRO PFAM
CTB-96E2.2	MMP24	0.0593793373554945	Shared protein domains	INTERPRO PFAM
CTB-96E2.2	MMP14	0.0593793373554945	Shared protein domains	INTERPRO PFAM
MMP11	MMP7	0.05570451356470585	Shared protein domains	INTERPRO PFAM
MMP15	CTB-96E2.2	0.05446958169341087	Shared protein domains	INTERPRO PFAM
MMP2	CTB-96E2.2	0.053628357127308846	Shared protein domains	INTERPRO PFAM
CTB-96E2.2	MMP9	0.05362806096673012	Shared protein domains	INTERPRO PFAM

MMP26	MMP28	0.047662071883678436	Shared protein domains	INTERPRO<br>PFAM
ABL1	SRC	0.03220065124332905	Shared protein domains	INTERPRO<br>PFAM
CDK9	CSF1R	0.006974334828555584	Shared protein domains	INTERPRO<br>PFAM
ABL1	CDK9	0.003097539534792304	Shared protein domains	INTERPRO
CDK9	SRC	0.003078958485275507	Shared protein domains	INTERPRO

Search results generated by the GeneMANIA algorithm (genemania.org)

GeneMANIA

Created on: 23 July 2015 10:31:00
Last database update: 1 June 2014 20:00:00
Application version: 3.1.2.8

Report of GeneMANIA search

Network image

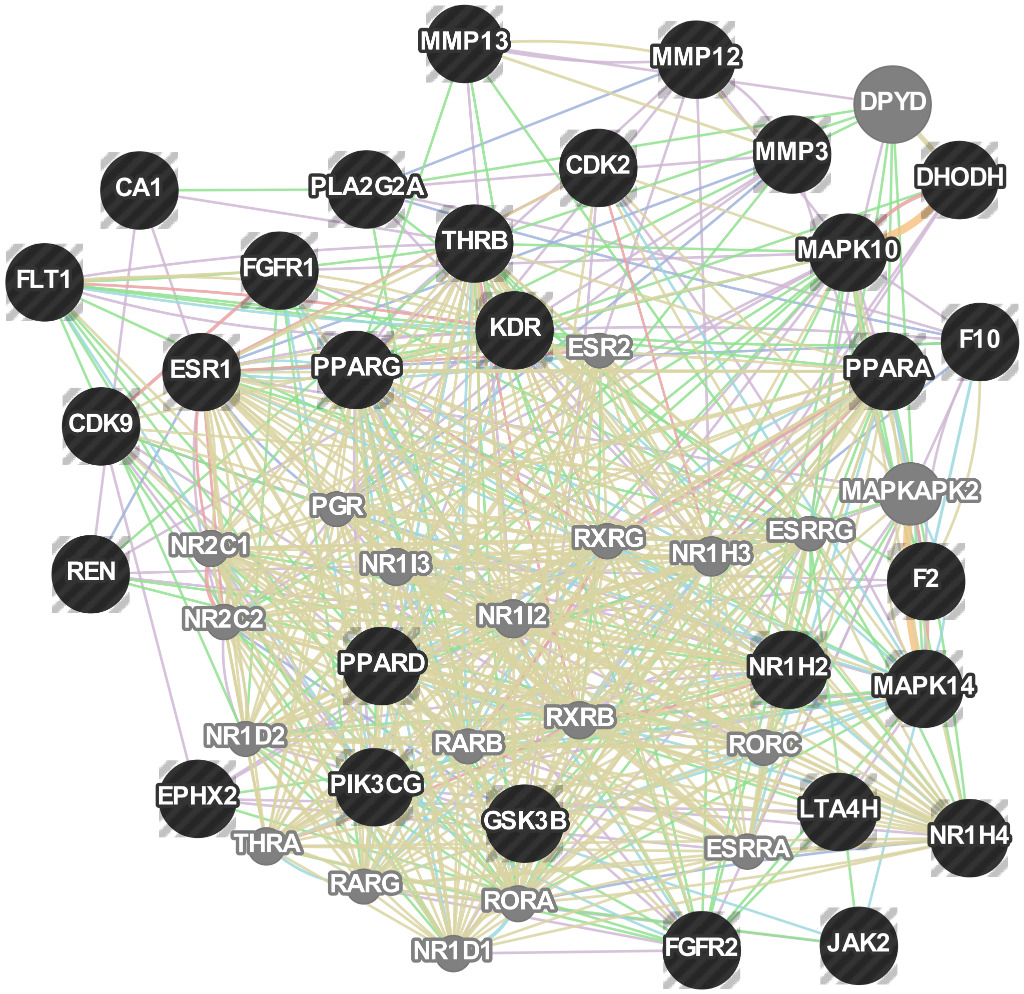

Functions legend
- query genes

Networks legend
- Co-expression
- Co-localization
- Genetic interactions
- Pathway
- Physical interactions
- Predicted
- Shared protein domains

Search parameters

Organism: H. sapiens (human)

Genes: Q96RI1; P21802; P11362; P49841; P03372; O60674; P17948; Q02127; P48736; P53779; P09960; Q03181; Q16539; P34913; P24941; P00742; P35968; P55055; P37231; P00915; P50750; P45452; P00797; P08254; P10828; P00734; Q07869; P14555; P39900

Networks:

Attributes:

Co-expression:
Alizadeh-Staudt-2000 Arijs-Rutgeerts-2009 Bahr-Bowler-2013 Bild-Nevins-2006 B Boldrick-Relman-2002 Burington-Shaughnessy-2008 Chen-Brown-2002 Cheok-Evans-2003 Gysin-McMahon-2012 Innocenti-Brown-2011 Kang-Willman-2010 Mallon-McKay-2013 Perou-Botstein-1999 Ramaswamy-Golub-2001 Rieger-Chu-2004 Roth-Zlotnik-2006 Salaverria-Siebert-2011 Smirnov-Cheung-2009 Wang-Maris-2006 Wu-Garvey-2007

Co-localization:
Johnson-Shoemaker-2003 Satoh-Yamamoto-2013 Schadt-Shoemaker-2004

Genetic interactions:
BIOGRID-SMALL-SCALE-STUDIES IREF-SMALL-SCALE-STUDIES Lin-Boeke-2012 A Lin-Boeke-2012 B Lin-Smith-2010 Toyoshima-Grandori-2012 Willingham-Muchowski-2003

Pathway:
PATHWAYCOMMONS-CELL_MAP PATHWAYCOMMONS-HUMANCYC PATHWAYCOMMONS-IMID PATHWAYCOMMONS-NCI_NATURE PATHWAYCOMMONS-REACTOME Wu-Stein-2010

Physical interactions:
Abu-Odeh-Aqeilan-2014 Agrawal-Sedivy-2010 Aichem-Groettrup-2012 Albers-Koegl-2005 Alexandru-Deshaies-2008 Altun-Kessler-2011 Andresen-Flores-Morales-2014 Arbuckle-Grant-2010 Arora-Mercola-2008 BIOGRID-SMALL-SCALE-STUDIES Bandyopadhyay-Ideker-2010 Bantscheff-Drewes-2011 Barr-Knapp-2009 Barrios-Rodiles-Wrana-2005 Behrends-Harper-2010 Behzadnia-Lührmann-2007 Bennett-Harper-2010 Benzinger-Hermeking-2005 Berggård-James-2006 Bett-Hay-2013 Blandin-Richard-2013 Bouwmeester-Superti-Furga-2004 Brajenovic-Drewes-2004 Brehme-Superti-Furga-2009 Bruderer-Hay-2011 Byron-Humphries-2012 Cai-Conaway-2007 Camargo-Brandon-2007 Cannavo-Jiricny-2007 Cao-Chinnaiyan-2014 Chen-Ge-2013 Chen-Naus-2012 Chen-Zhang-2013 Cheng-Chen-2010 Cloutier-Coulombe-2013 Colland-Gauthier-2004 Couzens-Gingras-2013 Cox-Rizzino-2013 Danielsen-Nielsen-2011 Dyer-Sobral-2010 Emanuele-Elledge-2011 Ewing-Figeys-2007 Fenner-Prehn-2010 Foerster-Ritter-2013 Foster-Marshall-2013 Freibaum-Taylor-2010 Gao-Reinberg-2012 Gautier-Hall-2009 Giannone-Liu-2010 Glatter-Gstaiger-2009 Gloeckner-Ueffing-2007 Goehler-Wanker-2004 Golebiowski-Hay-2009 Goudreault-Gingras-2009 Grant-2010 Greco-Cristea-2011 Havrylov-Redowicz-2009 Havugimana-Emili-2012 Hayes-Urbé-2012 Hegele-Stelzl-2012 A Hegele-Stelzl-2012 B Humphries-Humphries-2009 Hutchins-Peters-2010 IREF-BIND IREF-BIOGRID IREF-DIP IREF-HPRD IREF-INNATEDB IREF-INTACT IREF-MATRIXDB IREF-MPPI IREF-OPHID IREF-PUBMED IREF-SMALL-SCALE-STUDIES Ingham-Pawson-2005 Jeronimo-Coulombe-2007 Jin-Pawson-2004 Jones-MacBeath-2006 Joshi-Cristea-2013 Jäger-Krogan-2012 Kahle-Zoghbi-2011 Kim-Gygi-2011 Kneissl-Grummt-2003 Koch-Hermeking-2007 Kristensen-Foster-2012 Lau-Ronai-2012 Lee-Doedens-2011 Lee-Songyang-2011 Lehner-Sanderson-2004 A Lehner-Sanderson-2004 B Leng-Wang-2014 Li-Dorf-2011 A Li-Dorf-2011 B Lim-Zoghbi-2006 Liu-Wang-2012 Loch-Strickler-2012 Lopitz-Otsoa-Rodriguez-2012 Lu-Zhang-2013 Mak-Moffat-2010 Malovannaya-Qin-2010 Markson-Sanderson-2009 Maréchal-Zou-2014 Matafora-Bachi-2009 Matsumoto-Nakayama-2005 McCracken-Blencowe-2005 McFarland-Nussbaum-2008 Meek-Piwnica-Worms-2004 Meierhofer-Kaiser-2008 Miyamoto-Sato-Yanagawa-2010 Nakayama-Ohara-2002 Nakayasu-Adkins-2013 Napolitano-Meroni-2011 Nathan-Goldberg-2013 Neganova-Lako-2011 Newman-Keating-2003 Olma-Pintard-2009 Oláh-Ovádi-2011 Oshikawa-Nakayama-2012 Ouyang-Gill-2009 Panigrahi-Pati-2012 Perez-Hernandez-Yáñez-Mó-2013 Persaud-Rotin-2009 Pichlmair-Superti-Furga-2011 Pichlmair-Superti-Furga-2012 Pilot-Storck-Goillot-2010 Povlsen-Choudhary-2012 Ramachandran-LaBaer-2004 Ravasi-Hayashizaki-2010 Reinke-Keating-2013 Richter-Chrzanowska-Lightowlers-2010 Roux-Burke-2012 Rowbotham-Mermoud-2011 Roy-Parent-2013 Rual-Vidal-2005 A Rual-Vidal-2005 B San-Marina-Minden-2008 Sang-Jackson-2011 Sato-Conaway-2004 Shi-Qin-2011 Singh-Moore-2012 Soler-López-Aloy-2011 Sowa-Harper-2009 Stehling-Lill-2012 Stelzl-Wanker-2005 Suter-Wanker-2013 Taipale-Lindquist-2012 Takahashi-Conaway-2011 Tarallo-Weisz-2011 Tatham-Hay-2011 Teixeira-Gomes-2010 Thalappilly-Dusetti-2008 Tsai-Cristea-2012 Udeshi-Carr-2012 Vandamme-Angrand-2011 Vanderwerf-Bagby-2009 Varjosalo-Gstaiger-2013 A Varjosalo-Gstaiger-2013 B Varjosalo-Superti-Furga-2013 Venkatesan-Vidal-2009 Vinayagam-Wanker-2011 Wagner-Choudhary-2011 Wallach-Kramer-2013 Wang-Balch-2006 Wang-He-2008 Wang-Yang-2011 Weimann-Stelzl-2013 A

Weimann-Stelzl-2013 B Weinmann-Meister-2009 Wilker-Yaffe-2007 Wong-O'Bryan-2012 Woods-Monteiro-2012 Woodsmith-Sanderson-2012 Wu-Li-2007 Wu-Ma-2012 Xiao-Lefkowitz-2007 Xie-Cong-2013 Xu-Jaffrey-2010 Xu-Ye-2012 Yang-Chen-2010 Yatim-Benkirane-2012 Yu-Chow-2013 Yu-Vidal-2011 Zanon-Pichler-2013 Zhang-Zou-2011 Zhao-Krug-2005 Zhao-Yang-2011 Zhou-Conrads-2004 Zhou-Liang-2012 de Hoog-Mann-2004 van Wijk-Timmers-2009

Predicted:
I2D-BIND-Fly2Human I2D-BIND-Mouse2Human I2D-BIND-Rat2Human I2D-BIND-Worm2Human I2D-BIND-Yeast2Human I2D-BioGRID-Fly2Human I2D-BioGRID-Mouse2Human I2D-BioGRID-Rat2Human I2D-BioGRID-Worm2Human I2D-BioGRID-Yeast2Human I2D-Chen-Pawson-2009-PiwiScreen-Mouse2Human I2D-Formstecher-Daviet-2005-Embryo-Fly2Human I2D-Giot-Rothbert-2003-Low-Fly2Human I2D-INNATEDB-Mouse2Human I2D-IntAct-Fly2Human I2D-IntAct-Mouse2Human I2D-IntAct-Rat2Human I2D-IntAct-Worm2Human I2D-IntAct-Yeast2Human I2D-Krogan-Greenblatt-2006-Core-Yeast2Human I2D-Krogan-Greenblatt-2006-NonCore-Yeast2Human I2D-Li-Vidal-2004-CORE-1-Worm2Human I2D-Li-Vidal-2004-non-core-Worm2Human I2D-MGI-Mouse2Human I2D-MINT-Fly2Human I2D-MINT-Mouse2Human I2D-MINT-Rat2Human I2D-MINT-Worm2Human I2D-MINT-Yeast2Human I2D-Manual-Mouse2Human I2D-Manual-Rat2Human I2D-Ptacek-Snyder-2005-Yeast2Human I2D-Tarassov-PCA-Yeast2Human I2D-Tewari-Vidal-2004-TGFb-Worm2Human I2D-Wang-Orkin-2006-EScmplx-Mouse2Human I2D-Wang-Orkin-2006-EScmplxlow-Mouse2Human I2D-Yu-Vidal-2008-GoldStd-Yeast2Human I2D-vonMering-Bork-2002-High-Yeast2Human I2D-vonMering-Bork-2002-Low-Yeast2Human I2D-vonMering-Bork-2002-Medium-Yeast2Human Stuart-Kim-2003

Shared protein domains:
INTERPRO PFAM

Network weighting: Automatically selected weighting method (Assigned based on query genes)

Number of gene results: 20

Networks

**Shared protein domains**	**36.92 %**
**INTERPRO**	**23.32 %**

Source: Pearson correlation with 562,560 interactions from InterPro

PFAM — **13.60 %**

Source: Pearson correlation with 543,464 interactions from Pfam

Predicted — **25.37 %**

I2D-BioGRID-Fly2Human — **13.34 %**

BioGRID: a general repository for interaction datasets. Stark et al. (2006). *Nucleic Acids Res*.

Note: I2D predictions of protein protein interactions using BioGRID Drosophila melanogaster data

Source: Direct interaction with 4,100 interactions from I2D

I2D-MINT-Fly2Human — **8.54 %**

MINT: a Molecular INTeraction database. Zanzoni et al. (2002). *FEBS Lett*.

Note: I2D predictions of protein protein interactions using MINT Drosophila melanogaster data

Source: Direct interaction with 2,986 interactions from I2D

I2D-IntAct-Fly2Human — **3.49 %**

The IntAct molecular interaction database in 2010. Aranda et al. (2010). *Nucleic Acids Res*.

Note: I2D predictions of protein protein interactions using IntAct Drosophila melanogaster data

Source: Direct interaction with 3,934 interactions from I2D

Co-expression — **23.69 %**

Alizadeh-Staudt-2000 — **6.15 %**

Distinct types of diffuse large B-cell lymphoma identified by gene expression profiling. Alizadeh et al. (2000). *Nature*.

Source: Pearson correlation with 88,888 interactions from supplementary material

Tags: cultured cells; cancer

Ramaswamy-Golub-2001 — **4.78 %**

Multiclass cancer diagnosis using tumor gene expression signatures. Ramaswamy et al. (2001). *Proc Natl Acad Sci U S A*.

Source: Pearson correlation with 270,142 interactions from supplementary material

Tags: cancer

Wu-Garvey-2007 — **2.54 %**

The effect of insulin on expression of genes and biochemical pathways in human skeletal muscle. Wu et al. (2007). *Endocrine*.

Source: Pearson correlation with 260,762 interactions from GEO

Tags: transcription factors; muscle; cultured cells

Arijs-Rutgeerts-2009 — **2.37 %**

Mucosal gene expression of antimicrobial peptides in inflammatory bowel disease before and after first infliximab treatment. Arijs et al. (2009). *PLoS One*.

Source: Pearson correlation with 653,194 interactions from GEO

Tags: immune system

Roth-Zlotnik-2006 — **2.33 %**

Gene expression analyses reveal molecular relationships among 20 regions of the human CNS. Roth et al. (2006). *Neurogenetics*.

Source: Pearson correlation with 666,614 interactions from GEO

Gysin-McMahon-2012 **1.41 %**

Analysis of mRNA profiles after MEK1/2 inhibition in human pancreatic cancer cell lines reveals pathways involved in drug sensitivity. Gysin et al. (2012). *Mol Cancer Res*.

Source: Pearson correlation with 388,454 interactions from GEO

Tags: cell proliferation; cultured cells; cell line; signal transduction; cancer

Kang-Willman-2010 **1.38 %**

Gene expression classifiers for relapse-free survival and minimal residual disease improve risk classification and outcome prediction in pediatric B-precursor acute lymphoblastic leukemia. Kang et al. (2010). *Blood*.

Source: Pearson correlation with 656,632 interactions from GEO

Tags: transcription factors; lymphoma; cancer

Perou-Botstein-1999 **1.16 %**

Distinctive gene expression patterns in human mammary epithelial cells and breast cancers. Perou et al. (1999). *Proc Natl Acad Sci U S A*.

Source: Pearson correlation with 62,886 interactions from supplementary material

Tags: cultured cells; cancer; epithelial cells; signal transduction; breast; stromal cells; transcription factors; breast cancer

Boldrick-Relman-2002 **0.80 %**

Stereotyped and specific gene expression programs in human innate immune responses to bacteria. Boldrick et al. (2002). *Proc Natl Acad Sci U S A*.

Source: Pearson correlation with 108,543 interactions from supplementary material

Tags: immune system

Rieger-Chu-2004 **0.71 %**

Toxicity from radiation therapy associated with abnormal transcriptional responses to DNA damage. Rieger et al. (2004). *Proc Natl Acad Sci U S A*.

Source: Pearson correlation with 259,055 interactions from GEO

Tags: cultured cells; cell line

Smirnov-Cheung-2009 **0.05 %**

Genetic analysis of radiation-induced changes in human gene expression. Smirnov et al. (2009). *Nature*.

Source: Pearson correlation with 463,390 interactions from GEO

Tags: transcription factors; cultured cells; cell line

Cheok-Evans-2003 **0.01 %**

Treatment-specific changes in gene expression discriminate in vivo drug response in human leukemia cells. Cheok et al. (2003). *Nat Genet*.

Source: Pearson correlation with 263,940 interactions from GEO

Tags: chemotherapy; cultured cells; lymphoma; cancer

Physical interactions **6.99 %**

Varjosalo-Gstaiger-2013 A **2.93 %**

The protein interaction landscape of the human CMGC kinase group. Varjosalo et al. (2013). *Cell Rep*.

Note: One of 2 datasets produced from this publication.

Source: Direct interaction with 686 interactions from BioGRID

Tags: cultured cells; cell line

IREF-OPHID **2.17 %**

Source: Direct interaction with 44,492 interactions from iRefIndex

Cheng-Chen-2010 **1.89 %**

Quantitative nanoproteomics for protein complexes (QNanoPX) related to estrogen transcriptional action. Cheng et al. (2010). *Mol Cell Proteomics*.

Source: Direct interaction with 259 interactions from iRefIndex

Tags: transcription factors; cell line; cancer; cultured cells

Co-localization **2.61 %**

Schadt-Shoemaker-2004 **1.99 %**

A comprehensive transcript index of the human genome generated using microarrays and computational approaches. Schadt et al. (2004). *Genome Biol*.

Note: Predicted transcript array

Source: Pearson correlation with 60,216 interactions from GEO

Johnson-Shoemaker-2003 **0.62 %**

Genome-wide survey of human alternative pre-mRNA splicing with exon junction microarrays. Johnson et al. (2003). *Science*.

Source: Pearson correlation with 426,640 interactions from GEO

Tags: cultured cells; cell line

Pathway **2.36 %**

PATHWAYCOMMONS-REACTOME **1.42 %**

Source: Direct interaction with 24,930 interactions from Pathway Commons

PATHWAYCOMMONS-NCI_NATURE **0.66 %**

Source: Direct interaction with 10,109 interactions from Pathway Commons

Wu-Stein-2010 **0.28 %**

A human functional protein interaction network and its application to cancer data analysis. Wu et al. (2010). *Genome Biol*.

Source: 78,183 interactions from supplementary material

Tags: transcription factors; cancer

Genetic interactions **2.06 %**

Lin-Smith-2010 **2.06 %**

A genome-wide map of human genetic interactions inferred from radiation hybrid genotypes. Lin et al. (2010). *Genome Res*.

Source: 4,836,794 interactions from supplementary material

Attributes

Attribute	Gene

Genes

DHODH (Q02127) dihydroorotate dehydrogenase (quinone) [Source:HGNC Symbol;Acc:2867]

Functions:

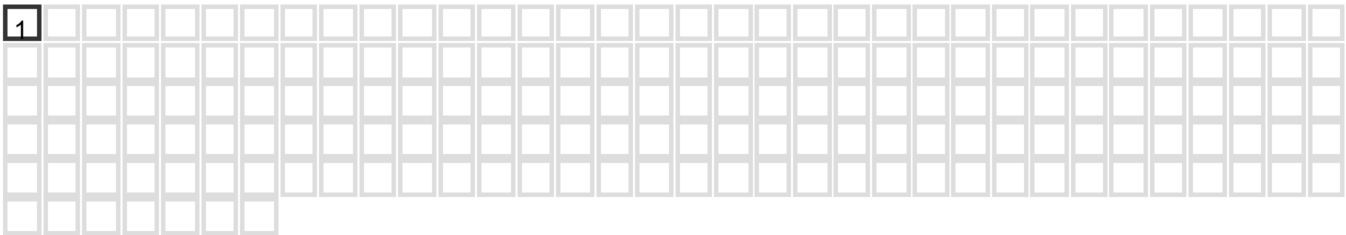

Synonyms: ENSG00000102967; ENSP00000219240; ENSP00000460966; ENSP00000461848; ENSP00000464249; ENSP00000464333; 1723; DHODH; NP_001352; NM_001361; PYRD_HUMAN; Q02127;

More at Entrez

NR1H4 (Q96RI1) nuclear receptor subfamily 1, group H, member 4 [Source:HGNC Symbol;Acc:7967]

Functions:

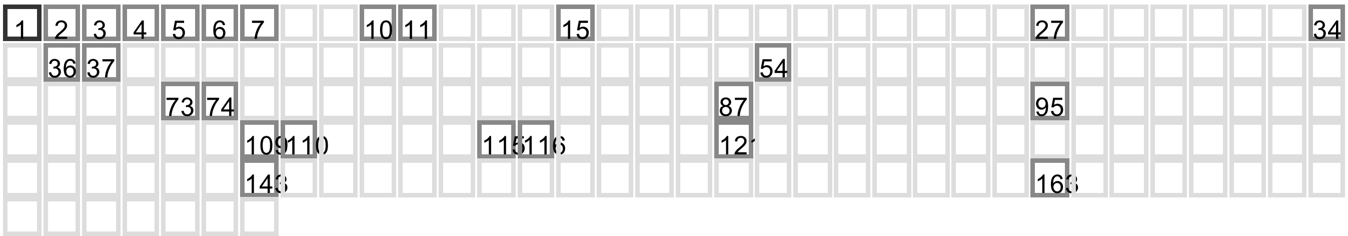

Synonyms: ENSG00000012504; ENSP00000188403; ENSP00000315442; ENSP00000376712; ENSP00000446584; ENSP00000446760; ENSP00000446861; ENSP00000447149; ENSP00000448506; ENSP00000448978; 9971; NR1H4; NP_001193906; NP_001193907; NP_001193908; NP_001193921; NP_001193922; NP_005114; NM_001206977; NM_001206978; NM_001206979; NM_001206992; NM_001206993; NM_005123; FXR; HRR-1; HRR1; RIP14; NR1H4_HUMAN; Q96RI1;

More at Entrez

PPARD (Q03181) peroxisome proliferator-activated receptor delta [Source:HGNC Symbol;Acc:9235]

Functions:

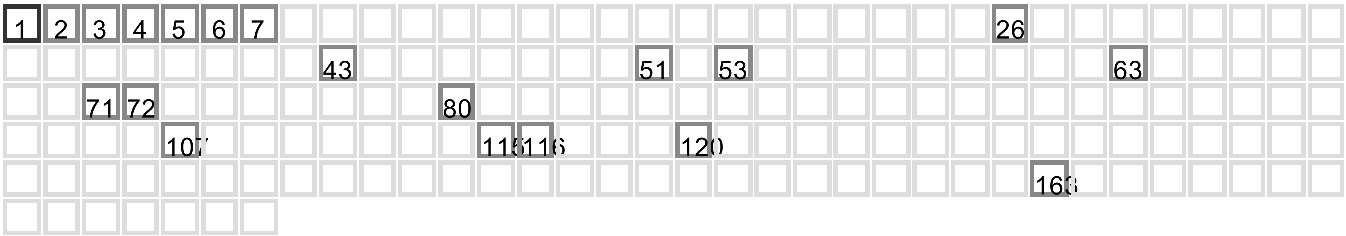

Synonyms: ENSG00000112033; ENSP00000310928; ENSP00000337063; ENSP00000353916; ENSP00000410837; ENSP00000413314; ENSP00000414372; ENSP00000443759; 5467; PPARD; NP_001165289; NP_001165290; NP_001165291; NP_006229; NP_803184; NM_001171818; NM_001171819; NM_001171820; NM_006238; NM_177435; FAAR; NR1C2; NUC1; NUCII; PPARD_HUMAN; Q03181;

More at Entrez

PPARA (Q07869) peroxisome proliferator-activated receptor alpha [Source:HGNC Symbol;Acc:9232]

Functions:

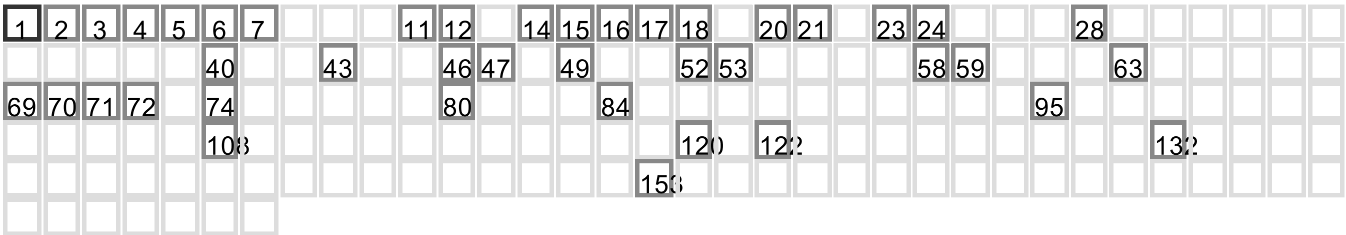

Synonyms: ENSG00000186951; ENSP00000262735; ENSP00000379322; ENSP00000385246; ENSP00000385523; ENSP00000397291; ENSP00000408149; ENSP00000411677; ENSP00000414752; 5465; PPARA; NP_001001928; NP_005027; NM_001001928; NM_005036; hPPAR; NR1C1; PPAR; PPARA_HUMAN;

Q07869;

More at Entrez

THRB (P10828) thyroid hormone receptor, beta [Source:HGNC Symbol;Acc:11799]

Functions:

1 2 3 4 5 6 7

Synonyms: ENSG00000151090; ENSP00000280696; ENSP00000348827; ENSP00000379904; ENSP00000388467; ENSP00000393932; ENSP00000395362; ENSP00000399573; ENSP00000404233; ENSP00000404898; ENSP00000414100; ENSP00000414444; ENSP00000414910; 7068; THRB; NP_000452; NP_001121648; NP_001121649; NP_001239563; NM_000461; NM_001128176; NM_001128177; NM_001252634; ERBA-BETA; ERBA2; NR1A2; PRTH; THR1; THRB1; THRB2; P10828; THB_HUMAN;

More at Entrez

FGFR1 (P11362) fibroblast growth factor receptor 1 [Source:HGNC Symbol;Acc:3688]

Functions:

1 22 29 30 31 33
42 45 56 61 64 65 66 68
76 78 81 88 91
105 118 119
151 152 160 161 169 170

Synonyms: ENSG00000077782; ENSP00000327229; ENSP00000337247; ENSP00000340636; ENSP00000348537; ENSP00000380280; ENSP00000380292; ENSP00000380297; ENSP00000380302; ENSP00000392645; ENSP00000393312; ENSP00000398318; ENSP00000400162; ENSP00000400708; ENSP00000432972; ENSP00000433163; ENSP00000433569; ENSP00000434473; ENSP00000434712; ENSP00000434800; ENSP00000434869; ENSP00000435254; ENSP00000435283; 2260; FGFR1; NP_001167534; NP_001167535; NP_001167536; NP_001167537; NP_001167538; NP_056934; NP_075593; NP_075594; NP_075598; NM_001174063; NM_001174064; NM_001174065; NM_001174066; NM_001174067; NM_015850; NM_023105; NM_023106; NM_023110; BFGFR; CD331; CEK; FLT2; KAL2; N-SAM; FGFR1_HUMAN; P11362;

More at Entrez

PPARG (P37231) peroxisome proliferator-activated receptor gamma [Source:HGNC Symbol;Acc:9236]

Functions:

1 2 3 4 5 6 7 12 13 14 15 17 18 20 21 23 24 25 26 27 28 32 34
35 36 37 44 47 48 51 55 58 59 60
70 72 79 80 83 85 90 92 97 98 99
107 108 116 117 120 132
137 138 149 153

Synonyms: ENSG00000132170; ENSP00000287820; ENSP00000312472; ENSP00000380195; ENSP00000380196; ENSP00000380205; ENSP00000380207; ENSP00000380210; ENSP00000380218; ENSP00000380221; ENSP00000380224; ENSP00000392285; ENSP00000411931; ENSP00000438940; 5468; PPARG; NP_005028; NP_056953; NP_619725; NP_619726; NM_005037; NM_015869; NM_138711; NM_138712; NR1C3; PPARG1; PPARG2; PPARgamma; P37231; PPARG_HUMAN;

More at Entrez

FGFR2 (P21802) fibroblast growth factor receptor 2 [Source:HGNC Symbol;Acc:3689]

Functions:

1 22 29 30 31 33
48 55 60 61 66
76 78 81 88 91

107 118 119
139 141 146 148 160 161 169 170

Synonyms: ENSG00000066468; ENSP00000263451; ENSP00000309878; ENSP00000337665; ENSP00000348559; ENSP00000350166; ENSP00000351276; ENSP00000352309; ENSP00000353262; ENSP00000358052; ENSP00000358054; ENSP00000358055; ENSP00000358056; ENSP00000358057; ENSP00000404219; ENSP00000410294; ENSP00000474011; ENSP00000474109; 2263; FGFR2; NP_000132; NP_001138385; NP_001138386; NP_001138387; NP_001138388; NP_001138389; NP_001138390; NP_001138391; NP_075259; NP_075418; NM_000141; NM_001144913; NM_001144914; NM_001144915; NM_001144916; NM_001144917; NM_001144918; NM_001144919; NM_022970; NM_023029; BEK; CD332; CEK3; ECT1; JWS; K-SAM; KGFR; TK14; TK25; FGFR2_HUMAN; P21802;

More at Entrez

NR1H2 (P55055) nuclear receptor subfamily 1, group H, member 2 [Source:HGNC Symbol;Acc:7965]

Functions:

1 2 3 11 12 14 17 18 20 21 22 23 25 30 32
35 43 46 47 57 58 59
69 70 73 74 79 80 83 85 87 90 92 95 97 98 99
106 109 110 117 122 130 134
137 138 142 154 156

Synonyms: ENSG00000131408; ENSP00000253727; ENSP00000396151; ENSP00000445074; ENSP00000469778; ENSP00000470518; ENSP00000471194; ENSP00000471294; ENSP00000472138; ENSP00000472271; ENSP00000472526; ENSP00000473099; 7376; NR1H2; NP_001243576; NP_009052; NM_001256647; NM_007121; LXR-b; NER; NER-I; RIP15; NR1H2_HUMAN; P55055;

More at Entrez

FLT1 (P17948) fms-related tyrosine kinase 1 [Source:HGNC Symbol;Acc:3763]

Functions:

1 11 22 29 30 31 33
38 42 45 46 50 56 61 64 65 66 68
76 78 81 88 91
105 118 119
151 152

Synonyms: ENSG00000102755; ENSP00000282397; ENSP00000437631; ENSP00000437841; ENSP00000442630; ENSP00000443311; 2321; FLT1; NP_001153392; NP_001153502; NP_001153503; NP_002010; NM_001160030; NM_001160031; NM_002019; FLT; VEGFR1; P17948; VGFR1_HUMAN;

More at Entrez

CA1 (P00915) carbonic anhydrase I [Source:HGNC Symbol;Acc:1368]

Functions:

1

Synonyms: ENSG00000133742; ENSP00000256119; ENSP00000392338; ENSP00000401551; ENSP00000427773; ENSP00000427852; ENSP00000428923; ENSP00000429300; ENSP00000429688; ENSP00000429798; ENSP00000429843; ENSP00000430372; ENSP00000430471; ENSP00000430543; ENSP00000430571; ENSP00000430656; ENSP00000430710; ENSP00000430737; ENSP00000430861; ENSP00000430975; ENSP00000443517; 759; CA1; NP_001122301; NP_001122302; NP_001122303; NP_001158302; NP_001729; NM_001128829; NM_001128830; NM_001128831; NM_001164830; NM_001738; CAH1_HUMAN; P00915;

More at Entrez

MMP13 (P45452) matrix metallopeptidase 13 (collagenase 3) [Source:HGNC Symbol;Acc:7159]

Functions:

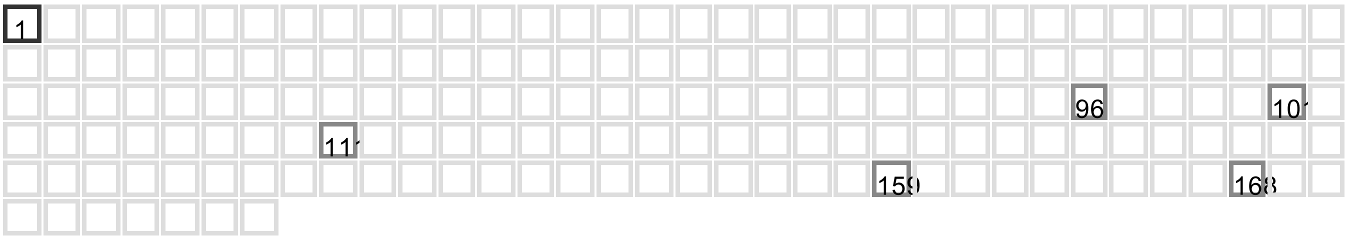

Synonyms: ENSG00000137745; ENSG00000262325; ENSP00000260302; ENSP00000339672; ENSP00000460695; ENSP00000461440; 4322; MMP13; NP_002418; NM_002427; CLG3; MMP13_HUMAN; P45452;

More at Entrez

REN (P00797) renin [Source:HGNC Symbol;Acc:9958]

Functions:

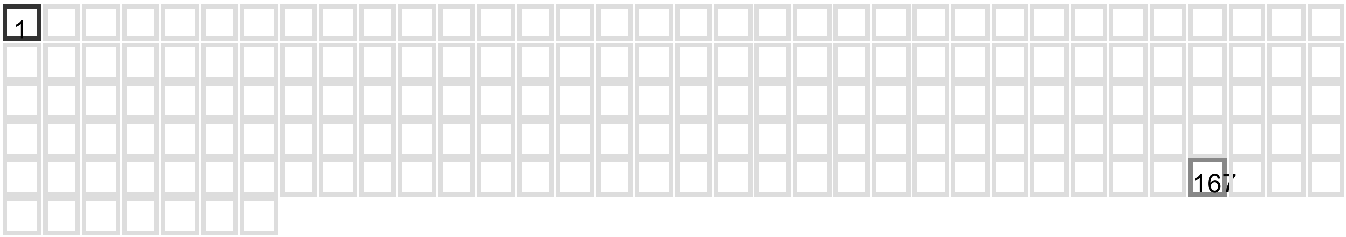

Synonyms: ENSG00000143839; ENSP00000272190; ENSP00000356163; 5972; REN; NP_000528; NM_000537; P00797; RENI_HUMAN;

More at Entrez

MMP3 (P08254) matrix metallopeptidase 3 (stromelysin 1, progelatinase) [Source:HGNC Symbol;Acc:7173]

Functions:

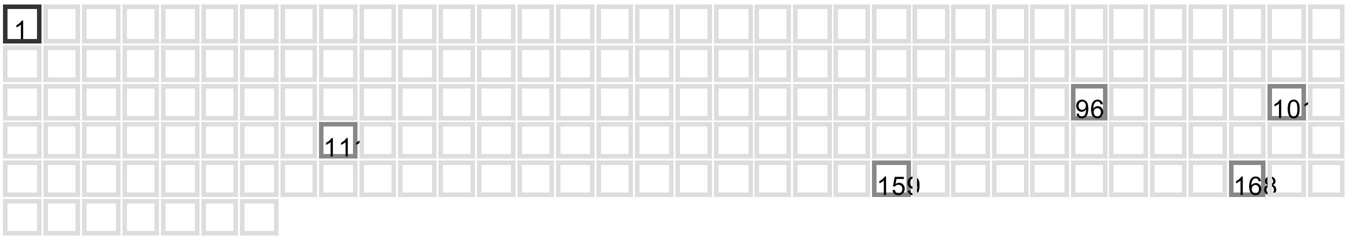

Synonyms: ENSG00000149968; ENSG00000263313; ENSP00000299855; ENSP00000398346; ENSP00000435255; ENSP00000458496; ENSP00000459512; ENSP00000459712; 4314; MMP3; NP_002413; NM_002422; STMY; STMY1; MMP3_HUMAN; P08254;

More at Entrez

LTA4H (P09960) leukotriene A4 hydrolase [Source:HGNC Symbol;Acc:6710]

Functions:

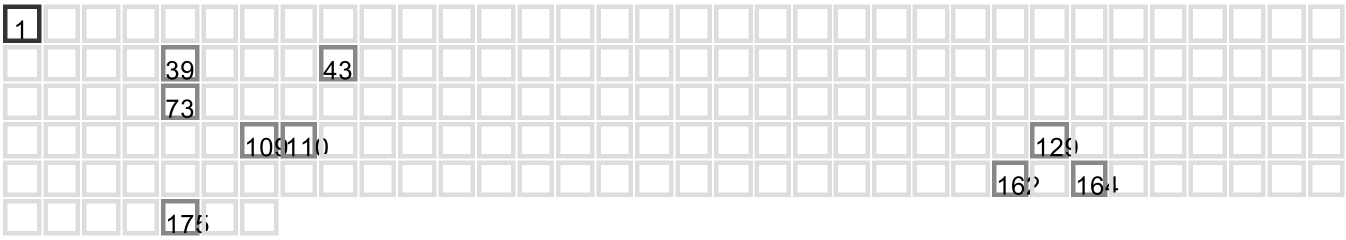

Synonyms: ENSG00000111144; ENSP00000228740; ENSP00000395051; ENSP00000449340; ENSP00000449958; 101928830; 4048; LTA4H; NP_000886; NP_001243572; NP_001243573; NM_000895; NM_001256643; NM_001256644; LKHA4_HUMAN; P09960;

More at Entrez

F10 (P00742) coagulation factor X [Source:HGNC Symbol;Acc:3528]

Functions:

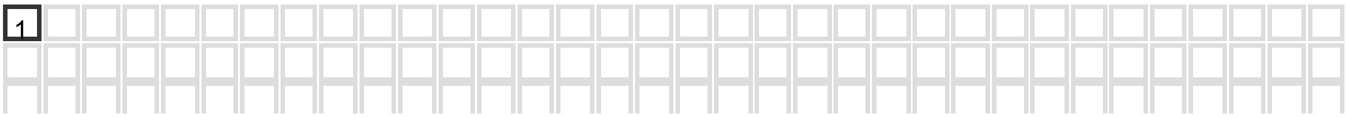

135 136
167

Synonyms: ENSG00000126218; ENSP00000364701; ENSP00000364709; ENSP00000386320; ENSP00000387092; 2159; F10; NP_000495; NM_000504; FA10_HUMAN; P00742;

More at Entrez

MMP12 (P39900) matrix metallopeptidase 12 (macrophage elastase) [Source:HGNC Symbol;Acc:7158]

Functions:

1
96 101
111
159 167 168

Synonyms: ENSG00000262406; ENSP00000458585; 4321; MMP12; NP_002417; NM_002426; HME; MMP12_HUMAN; P39900;

More at Entrez

PLA2G2A (P14555) phospholipase A2, group IIA (platelets, synovial fluid) [Source:HGNC Symbol;Acc:9031]

Functions:

1 12 14 17
39
77 94
147

Synonyms: ENSG00000188257; ENSP00000364252; ENSP00000383364; 5320; PLA2G2A; NP_000291; NP_001155199; NP_001155200; NP_001155201; NM_000300; NM_001161727; NM_001161728; NM_001161729; PLA2B; P14555; PA2GA_HUMAN;

More at Entrez

JAK2 (O60674) Janus kinase 2 [Source:HGNC Symbol;Acc:6192]

Functions:

1 13 25 31 32 33
35 39 42 48 55 56 60 61 65
79 81 92 94 97 99 100
112 114 117 118 119
140 144 145 147
172 176

Synonyms: ENSG00000096968; ENSP00000371067; ENSP00000440387; ENSP00000443103; 3717; JAK2; NP_004963; NM_004972; JTK10; JAK2_HUMAN; O60674;

More at Entrez

EPHX2 (P34913) epoxide hydrolase 2, cytoplasmic [Source:HGNC Symbol;Acc:3402]

Functions:

1 11
39 43 44
115 121 124 127 129
143 162 163 164
175

Synonyms: ENSG00000120915; ENSP00000369843; ENSP00000427956; ENSP00000428191; ENSP00000428875; ENSP00000430269; ENSP00000430302; ENSP00000430779; 2053; EPHX2;

NP_001243411; NP_001243412; NP_001243413; NP_001970; NM_001256482; NM_001256483; NM_001256484; NM_001979; HYES_HUMAN; P34913;

More at Entrez

PIK3CG (P48736) phosphatidylinositol-4,5-bisphosphate 3-kinase, catalytic subunit gamma [Source:HGNC Symbol;Ac

Functions:

1 29 31 33
39 42 45 64 68
105

Synonyms: ENSG00000105851; ENSP00000352121; ENSP00000392258; ENSP00000417623; ENSP00000419260; 5294; PIK3CG; NP_001269355; NP_001269356; NP_002640; NM_001282426; NM_001282427; NM_002649; P48736; PK3CG_HUMAN;

More at Entrez

F2 (P00734) coagulation factor II (thrombin) [Source:HGNC Symbol;Acc:3535]

Functions:

1
59
96 101
111 135 136
167

Synonyms: ENSG00000180210; ENSP00000308541; ENSP00000387413; ENSP00000433907; 2147; F2; NP_000497; NM_000506; P00734; THRB_HUMAN;

More at Entrez

KDR (P35968) kinase insert domain receptor (a type III receptor tyrosine kinase) [Source:HGNC Symbol;Acc:6307]

Functions:

1 29 31 33
38 42 48 50 55 56 60 61 65 66
76 81 91
112 118 119
144 145 148
176

Synonyms: ENSG00000128052; ENSP00000263923; 3791; KDR; NP_002244; NM_002253; CD309; FLK1; VEGFR; VEGFR2; P35968; VGFR2_HUMAN;

More at Entrez

GSK3B (P49841) glycogen synthase kinase 3 beta [Source:HGNC Symbol;Acc:4617]

Functions:

1 8 19 31 33
40 49 52 53 59 63
71 84
104 112
160 161 169
173 176

Synonyms: ENSG00000082701; ENSP00000264235; ENSP00000324806; 2932; GSK3B; NP_001139628; NP_002084; NM_001146156; NM_002093; GSK3B_HUMAN; P49841;

More at Entrez

MAPK10 (P53779) mitogen-activated protein kinase 10 [Source:HGNC Symbol;Acc:6872]

Functions:

1 25 29
45 62 64 68
75 77 86 89 93
104 105 113 125
155 157 158 165 166 169
171

Synonyms: ENSG00000109339; ENSP00000309857; ENSP00000352157; ENSP00000355297; ENSP00000378586; ENSP00000378589; ENSP00000378590; ENSP00000378595; ENSP00000378598; ENSP00000414469; ENSP00000420987; ENSP00000421359; ENSP00000421409; ENSP00000421762; ENSP00000422277; ENSP00000422985; ENSP00000423918; ENSP00000424128; ENSP00000424154; ENSP00000424755; ENSP00000425654; 5602; MAPK10; NP_002744; NP_620446; NP_620447; NP_620448; NM_002753; NM_138980; NM_138981; NM_138982; JNK3; p493F12; p54bSAPK; PRKM10; MK10_HUMAN; P53779;

More at Entrez

CDK9 (P50750) cyclin-dependent kinase 9 [Source:HGNC Symbol;Acc:1780]

Functions:

1 2 3
104

Synonyms: ENSG00000136807; ENSP00000362361; ENSP00000362362; ENSP00000395872; 1025; CDK9; NP_001252; NM_001261; C-2k; CDC2L4; PITALRE; TAK; CDK9_HUMAN; P50750;

More at Entrez

ESR1 (P03372) estrogen receptor 1 [Source:HGNC Symbol;Acc:3467]

Functions:

1 2 3 4 5 6 7 10 13 15 16 22 30 34
36 37 41 54 67
78 82 88 102
151 152
177

Synonyms: ENSG00000091831; ENSP00000206249; ENSP00000342630; ENSP00000384064; ENSP00000385373; ENSP00000387500; ENSP00000394721; ENSP00000401995; ENSP00000405330; ENSP00000411105; ENSP00000415934; 2099; ESR1; NP_000116; NP_001116212; NP_001116213; NP_001116214; NM_000125; NM_001122740; NM_001122741; NM_001122742; Era; ESR; NR3A1; ESR1_HUMAN; P03372;

More at Entrez

MAPK14 (Q16539) mitogen-activated protein kinase 14 [Source:HGNC Symbol;Acc:6876]

Functions:

1 25 29
38 45 48 50 55 60 62 64 68
75 77 86 89 93
104 105 113 125 126
139 141 146 155 157 158 160 161 165 166
171

Synonyms: ENSG00000112062; ENSP00000229794; ENSP00000229795; ENSP00000308669; ENSP00000417065; ENSP00000417531; ENSP00000419141; ENSP00000419837; 1432; MAPK14; NP_001306; NP_620581; NP_620582; NP_620583; NM_001315; NM_139012; NM_139014; CSBP1; CSBP2; CSPB1; Mxi2; PRKM14; PRKM15; MK14_HUMAN; Q16539;

More at Entrez

CDK2 (P24941) cyclin-dependent kinase 2 [Source:HGNC Symbol;Acc:1771]

Functions:

1 104 140 172

Synonyms: ENSG00000123374; ENSP00000243067; ENSP00000266970; ENSP00000393605; ENSP00000450983; ENSP00000452138; ENSP00000452514; 1017; CDK2; NP_001789; NP_439892; NM_001798; NM_052827; CDK2_HUMAN; P24941;

More at Entrez

DPYD dihydropyrimidine dehydrogenase [Source:HGNC Symbol;Acc:3012] 5.89

Functions:

Synonyms: ENSG00000188641; ENSP00000307107; ENSP00000359211; ENSP00000398884; 1806; DPYD; NP_000101; NP_001153773; NM_000110; NM_001160301; DPD; DPYD_HUMAN; Q12882;

More at Entrez

MAPKAPK2 mitogen-activated protein kinase-activated protein kinase 2 [Source:HGNC Symbol;Acc:6887] 4.92

Functions:

13 25 29 38 39 43 45 50 57 62 64 68 75 77 86 89 93 94 100 104 105 113 114 126 129 157 158 160 161 162 164 165 166 171 175

Synonyms: ENSG00000162889; ENSG00000266641; ENSP00000294981; ENSP00000356070; ENSP00000461936; ENSP00000462387; 9261; MAPKAPK2; NP_004750; NP_116584; NM_004759; NM_032960; MAPK2_HUMAN; P49137;

More at Entrez

NR1H3 nuclear receptor subfamily 1, group H, member 3 [Source:HGNC Symbol;Acc:7966] 3.43

Functions:

2 3 8 9 10 11 12 13 14 17 18 19 20 21 22 23 24 25 27 28 30 32 34 35 36 37 39 43 44 46 47 57 58 59 62 69 70 73 74 75 77 79 80 82 83 85 86 87 89 90 92 93 94 95 97 98 99 100 103 106 108 109 110 113 114 117 122 124 127 128 130 133 134 137 138 142 147 154 156 173

Synonyms: ENSG00000025434; ENSP00000378793; ENSP00000384745; ENSP00000385073; ENSP00000385801; ENSP00000387946; ENSP00000391005; ENSP00000396132; ENSP00000403696; ENSP00000403798; ENSP00000406692; ENSP00000407716; ENSP00000412636; ENSP00000413095; ENSP00000415591; ENSP00000420656; ENSP00000432073; ENSP00000433271; ENSP00000434650; 10062; NR1H3; NP_001123573; NP_001123574; NP_001238863; NP_001238864; NP_005684; NM_001130101; NM_001130102; NM_005693; LXR-a; RLD-1; NR1H3_HUMAN; Q13133;

More at Entrez

NR1I2 nuclear receptor subfamily 1, group I, member 2 [Source:HGNC Symbol;Acc:7968] 3.36

Functions:

2 3 4 5 6 7 27 72 115

Synonyms: ENSG00000144852; ENSP00000336528; ENSP00000377319; ENSP00000420297; 8856; NR1I2; NP_003880; NP_071285; NP_148934; NM_003889; NM_022002; NM_033013; BXR; ONR1; PXR; SXR; NR1I2_HUMAN; O75469;

More at Entrez

THRA thyroid hormone receptor, alpha [Source:HGNC Symbol;Acc:11796] 3.32

Functions:

2 3 4 5 7 34 36 37

Synonyms: ENSG00000126351; ENSP00000264637; ENSP00000377679; ENSP00000395641; ENSP00000443972; ENSP00000462955; ENSP00000463466; ENSP00000464013; ENSP00000464123; ENSP00000464414; 7067; THRA; NP_001177847; NP_001177848; NP_003241; NP_955366; NM_001190918; NM_001190919; NM_003250; NM_199334; AR7; EAR-7.1/EAR-7.2; ERBA; ERBA1; NR1A1; THRA2; THRA3; P10827; THA_HUMAN;

More at Entrez

RXRB retinoid X receptor, beta [Source:HGNC Symbol;Acc:10478] 3.31

Functions:

2 3 4 5 7 27

Synonyms: ENSG00000204231; ENSG00000206289; ENSG00000227322; ENSG00000228333; ENSG00000231321; ENSG00000235712; ENSP00000363812; ENSP00000363817; ENSP00000372703; ENSP00000372704; ENSP00000389514; ENSP00000392937; ENSP00000393286; ENSP00000393870; ENSP00000400104; ENSP00000402506; ENSP00000402590; ENSP00000406246; ENSP00000410433; ENSP00000410468; ENSP00000411238; ENSP00000411729; ENSP00000415199; ENSP00000415561; ENSP00000431353; ENSP00000431369; ENSP00000432690; ENSP00000433265; ENSP00000434208; ENSP00000435434; ENSP00000435471; ENSP00000439222; ENSP00000446618; ENSP00000446888; ENSP00000447039; ENSP00000447135; ENSP00000447315; ENSP00000448491; ENSP00000449125; ENSP00000449719; ENSP00000450209; ENSP00000450327; 6257; RXRB; NP_001257330; NP_068811; NM_001270401; NM_021976; H-2RIIBP; NR2B2; RCoR-1; P28702; RXRB_HUMAN;

More at Entrez

NR1I3 nuclear receptor subfamily 1, group I, member 3 [Source:HGNC Symbol;Acc:7969] 3.30

Functions:

2 3 4 5 6 7 16 27

Synonyms: ENSG00000143257; ENSP00000356958; ENSP00000356959; ENSP00000356960; ENSP00000356961; ENSP00000356962; ENSP00000356963; ENSP00000356965; ENSP00000399361;

ENSP00000406493; ENSP00000407446; ENSP00000412672; ENSP00000421374; ENSP00000421588; ENSP00000422982; ENSP00000423007; ENSP00000423089; ENSP00000423666; ENSP00000424345; ENSP00000424834; ENSP00000424934; ENSP00000425417; ENSP00000425607; ENSP00000425900; ENSP00000426016; ENSP00000426292; ENSP00000427034; ENSP00000427175; ENSP00000427600; 9970; NR1I3; NP_001070937; NP_001070938; NP_001070939; NP_001070940; NP_001070941; NP_001070942; NP_001070943; NP_001070944; NP_001070945; NP_001070946; NP_001070947; NP_001070948; NP_001070949; NP_001070950; NP_005113; NM_001077469; NM_001077470; NM_001077471; NM_001077472; NM_001077473; NM_001077474; NM_001077475; NM_001077476; NM_001077477; NM_001077478; NM_001077479; NM_001077480; NM_001077481; NM_001077482; NM_005122; MB67; NR1I3_HUMAN; Q14994;

More at Entrez

NR1D1 nuclear receptor subfamily 1, group D, member 1 [Source:HGNC Symbol;Acc:7962] 3.25

Functions:

2 3 4 5 6 7 8 9 11 13 15 19 24 25 26 28 34
36 37 40 41 44 46 49 51 52 53 54 62 63 67
71 73 74 75 77 82 84 86 87 89 93 94 95 100
106 108 109 110 113 114 115 121 124 127 133
173

Synonyms: ENSG00000126368; ENSP00000246672; 9572; NR1D1; NP_068370; NM_021724; ear-1; hRev; Rev-ErbAalpha; THRAL; NR1D1_HUMAN; P20393;

More at Entrez

NR2C1 nuclear receptor subfamily 2, group C, member 1 [Source:HGNC Symbol;Acc:7971] 3.25

Functions:

2 3

Synonyms: ENSG00000120798; ENSP00000328843; ENSP00000333275; ENSP00000376813; ENSP00000446817; ENSP00000446906; ENSP00000450225; 7181; NR2C1; NP_001027458; NP_001120834; NP_003288; NM_001032287; NM_001127362; NM_003297; TR2-11; NR2C1_HUMAN; P13056;

More at Entrez

RORA RAR-related orphan receptor A [Source:HGNC Symbol;Acc:10258] 3.22

Functions:

2 3 4 6 8 9 10 13 19 26
40 48 49 51 52 53 55 59 60 63
71 82 84 102
103 123 128 131
150
174 177

Synonyms: ENSG00000069667; ENSP00000261523; ENSP00000309753; ENSP00000335087; ENSP00000402971; ENSP00000449482; ENSP00000453322; 6095; RORA; NP_002934; NP_599022; NP_599023; NP_599024; NM_002943; NM_134260; NM_134261; NM_134262; NR1F1; ROR3; RZRA; P35398; RORA_HUMAN;

More at Entrez

RORC RAR-related orphan receptor C [Source:HGNC Symbol;Acc:10260] 3.20

Functions:

2 3 4 6 8 9 10 13 19 26
40 48 49 51 52 53 55 60 63
71 82 84 102

108 12[illegible] 128 131 150 174 177

Synonyms: ENSG00000143365; ENSP00000327025; ENSP00000349164; ENSP00000376461; 6097; RORC; NP_001001523; NP_005051; NM_001001523; NM_005060; NR1F3; RORG; RZRG; TOR; P51449; RORG_HUMAN;

More at Entrez

NR2C2 nuclear receptor subfamily 2, group C, member 2 [Source:HGNC Symbol;Acc:7972] 3.19

Functions:

2 3 15 27

Synonyms: ENSG00000177463; ENSP00000320447; ENSP00000376814; ENSP00000384463; ENSP00000388387; ENSP00000391473; ENSP00000401807; ENSP00000402272; ENSP00000412473; ENSP00000413438; 7182; NR2C2; NP_003289; NM_003298; hTAK1; TR2R1; TR4; NR2C2_HUMAN; P49116;

More at Entrez

RXRG retinoid X receptor, gamma [Source:HGNC Symbol;Acc:10479] 3.18

Functions:

2 3

Synonyms: ENSG00000143171; ENSP00000352900; 6258; RXRG; NP_001243499; NP_001243500; NP_008848; NM_001256570; NM_006917; NR2B3; P48443; RXRG_HUMAN;

More at Entrez

ESRRA estrogen-related receptor alpha [Source:HGNC Symbol;Acc:3471] 3.18

Functions:

2 3 4 5 6 7 15

Synonyms: ENSG00000173153; ENSP00000000442; ENSP00000384851; ENSP00000385971; ENSP00000439896; ENSP00000441970; ENSP00000444710; 2101; ESRRA; NP_001269379; NP_001269380; NP_004442; NM_001282450; NM_001282451; NM_004451; ERR1; ERRa; ERRalpha; ESRL1; NR3B1; ERR1_HUMAN; P11474;

More at Entrez

NR1D2 nuclear receptor subfamily 1, group D, member 2 [Source:HGNC Symbol;Acc:7963] 3.16

Functions:

2 3 4 5 6 7 8 9 11 15 19 34 36 37 39 41 44 54 67 139 141 146 147

Synonyms: ENSG00000174738; ENSP00000310006; ENSP00000373283; 9975; NR1D2; NP_001138897; NP_005117; NM_001145425; NM_005126; BD73; EAR-1r; Hs.37288; RVR; NR1D2_HUMAN; Q14995;

More at Entrez

RARG retinoic acid receptor, gamma [Source:HGNC Symbol;Acc:9866] 3.16

Functions:

2 3 6 13 148 149

Synonyms: ENSG00000172819; ENSP00000332695; ENSP00000343698; ENSP00000377947; ENSP00000388510; ENSP00000444335; ENSP00000454385; ENSP00000454417; ENSP00000454604; 5916; RARG; NP_000957; NP_001036193; NP_001230659; NP_001230660; NP_001230661; NM_000966; NM_001042728; NM_001243730; NM_001243731; NM_001243732; NR1B3; RARC; P13631; RARG_HUMAN;

More at Entrez

RARB retinoic acid receptor, beta [Source:HGNC Symbol;Acc:9865] 3.15

Functions:

2 3 148

Synonyms: ENSG00000077092; ENSP00000332296; ENSP00000373282; ENSP00000385865; ENSP00000391391; ENSP00000398840; 5915; RARB; NP_000956; NP_057236; NM_000965; NM_016152; NR1B2; RRB2; P10826; RARB_HUMAN;

More at Entrez

PGR progesterone receptor [Source:HGNC Symbol;Acc:8910] 3.14

Functions:

2 3

Synonyms: ENSG00000082175; ENSP00000263463; ENSP00000325120; ENSP00000432352; ENSP00000432914; ENSP00000436561; ENSP00000436803; 5241; PGR; NP_000917; NP_001189403; NP_001258090; NP_001258091; NM_000926; NM_001202474; NM_001271161; NM_001271162; NR3C3; P06401; PRGR_HUMAN;

More at Entrez

ESRRG estrogen-related receptor gamma [Source:HGNC Symbol;Acc:3474] 3.14

Functions:

2 3

Synonyms: ENSG00000196482; ENSP00000352077; ENSP00000353108; ENSP00000354584; ENSP00000355225; ENSP00000355904; ENSP00000355905; ENSP00000355907; ENSP00000375761;

ENSP00000386171; ENSP00000417374; ENSP00000417900; ENSP00000418629; ENSP00000418895; ENSP00000419155; ENSP00000419514; ENSP00000419594; ENSP00000420370; ENSP00000466343; 2104; ESRRG; NP_001127757; NP_001230434; NP_001230435; NP_001230436; NP_001230438; NP_001230439; NP_001230440; NP_001230441; NP_001230442; NP_001230443; NP_001230444; NP_001230447; NP_001230448; NP_001429; NP_996317; NP_996318; NM_001134285; NM_001243505; NM_001243506; NM_001243507; NM_001243509; NM_001243510; NM_001243511; NM_001243512; NM_001243513; NM_001243514; NM_001243515; NM_001243518; NM_001243519; NM_001438; NM_206594; NM_206595; ERR3_HUMAN; P62508;

More at Entrez

ESR2 estrogen receptor 2 (ER beta) [Source:HGNC Symbol;Acc:3468] 3.14

Functions:

2 3 4 5 6 7 10 15 16 27 34
36 37 41 54 67

Synonyms: ENSG00000140009; ENSP00000267525; ENSP00000335551; ENSP00000343925; ENSP00000345616; ENSP00000350427; ENSP00000351412; ENSP00000441792; ENSP00000450488; ENSP00000450699; ENSP00000451582; ENSP00000452426; ENSP00000452485; 2100; ESR2; NP_001035365; NP_001035366; NP_001201831; NP_001201832; NP_001258805; NP_001258806; NP_001428; NM_001040275; NM_001040276; NM_001214902; NM_001214903; NM_001271876; NM_001271877; NM_001437; Erb; NR3A2; ESR2_HUMAN; Q92731;

More at Entrez

Functions

Function	FDR	Coverage
1 query genes	n/a	29 / 29
2 transcription initiation from RNA polymerase II promoter	8.83E-36	26 / 189
3 DNA-templated transcription, initiation	1.04E-34	26 / 212
4 direct ligand regulated sequence-specific DNA binding transcription factor activity	4.74E-30	16 / 35
5 ligand-activated sequence-specific DNA binding RNA polymerase II transcription factor activity	2.24E-25	14 / 33
6 intracellular receptor signaling pathway	6.92E-15	15 / 206
7 sequence-specific DNA binding RNA polymerase II transcription factor activity	1.51E-13	14 / 199
8 circadian rhythm	2.62E-6	6 / 42
9 regulation of circadian rhythm	9.8E-6	5 / 25
10 steroid binding	9.8E-6	6 / 54
11 regulation of lipid metabolic process	1.34E-5	8 / 162
12 regulation of macrophage derived foam cell differentiation	1.41E-5	5 / 28
13 response to lipid	1.66E-5	9 / 248
14 foam cell differentiation	1.66E-5	5 / 31
15 sequence-specific DNA binding	1.66E-5	9 / 254
16 steroid hormone receptor activity	1.66E-5	4 / 11
17 macrophage derived foam cell differentiation	1.66E-5	5 / 31
18 regulation of cholesterol storage	2.02E-5	4 / 12
19 rhythmic process	2.21E-5	6 / 69
20 cholesterol storage	2.48E-5	4 / 13
21 negative regulation of macrophage derived foam cell differentiation	2.48E-5	4 / 13
22 positive regulation of lipase activity	3.99E-5	6 / 78
23 negative regulation of lipid storage	5.71E-5	4 / 16
24 regulation of receptor biosynthetic process	9.14E-5	4 / 18
25 regulation of innate immune response	1.3E-4	8 / 243
26 regulation of fat cell differentiation	1.5E-4	5 / 52
27 transcription coactivator activity	1.5E-4	8 / 249
28 receptor biosynthetic process	1.51E-4	4 / 21
29 positive regulation of MAPK cascade	1.51E-4	8 / 253
30 regulation of lipase activity	1.53E-4	6 / 103
31 phosphatidylinositol-mediated signaling	1.56E-4	7 / 173
32 regulation of interferon-gamma-mediated signaling pathway	1.56E-4	4 / 22
33 inositol lipid-mediated signaling	1.56E-4	7 / 173
34 regulatory region DNA binding	1.78E-4	8 / 267
35 regulation of response to interferon-gamma	1.78E-4	4 / 23
36 transcription regulatory region DNA binding	1.78E-4	8 / 266
37 regulatory region nucleic acid binding	1.78E-4	8 / 267
38 cellular response to vascular endothelial growth factor stimulus	1.95E-4	4 / 24
39 inflammatory response	2.55E-4	8 / 282
40 regulation of glucose metabolic process	2.59E-4	5 / 63
41 core promoter sequence-specific DNA binding	2.59E-4	4 / 26
42 phosphatidylinositol 3-kinase signaling	3.71E-4	5 / 68
43 fatty acid metabolic process	4.12E-4	7 / 209
44 lipid homeostasis	4.12E-4	5 / 70
	5.21E-4	6 / 136

45	positive regulation of MAP kinase activity		
46	positive regulation of lipid metabolic process	6.71E-4	5 / 78
47	regulation of lipid storage	6.72E-4	4 / 34
48	hemopoiesis	7.56E-4	7 / 232
49	regulation of cellular carbohydrate metabolic process	7.59E-4	5 / 81
50	vascular endothelial growth factor receptor signaling pathway	8.94E-4	4 / 37
51	fat cell differentiation	9.81E-4	5 / 86
52	regulation of carbohydrate metabolic process	1.02E-3	5 / 87
53	glucose metabolic process	1.12E-3	6 / 161
54	transcription regulatory region sequence-specific DNA binding	1.12E-3	5 / 90
55	hematopoietic or lymphoid organ development	1.12E-3	7 / 250
56	positive regulation of phosphatidylinositol 3-kinase signaling	1.12E-3	4 / 40
57	pinocytosis	1.14E-3	3 / 12
58	lipid storage	1.42E-3	4 / 43
59	negative regulation of cell differentiation	1.49E-3	7 / 266
60	immune system development	1.49E-3	7 / 266
61	protein tyrosine kinase activity	1.56E-3	5 / 98
62	toll-like receptor 4 signaling pathway	1.99E-3	5 / 104
63	hexose metabolic process	1.99E-3	6 / 182
64	positive regulation of protein serine/threonine kinase activity	1.99E-3	6 / 182
65	regulation of phosphatidylinositol 3-kinase signaling	2.15E-3	4 / 49
66	transmembrane receptor protein tyrosine kinase activity	2.26E-3	4 / 50
67	core promoter binding	2.26E-3	4 / 50
68	regulation of MAP kinase activity	2.44E-3	6 / 191
69	positive regulation of fatty acid metabolic process	2.89E-3	3 / 17
70	regulation of lipid transport	3.17E-3	4 / 55
71	monosaccharide metabolic process	3.85E-3	6 / 209
72	drug binding	3.85E-3	4 / 58
73	monocarboxylic acid biosynthetic process	4.1E-3	5 / 124
74	regulation of cellular ketone metabolic process	4.89E-3	5 / 129
75	toll-like receptor signaling pathway	5.8E-3	5 / 134
76	transmembrane receptor protein kinase activity	5.96E-3	4 / 66
77	positive regulation of defense response	5.96E-3	6 / 228
78	positive regulation of phospholipase activity	8.3E-3	4 / 72
79	interferon-gamma-mediated signaling pathway	8.54E-3	4 / 73
80	lipid transport	8.54E-3	5 / 147
81	protein autophosphorylation	8.82E-3	5 / 149
82	cellular response to lipid	8.82E-3	5 / 149
83	regulation of sterol transport	9.96E-3	3 / 27
84	cellular carbohydrate metabolic process	9.96E-3	5 / 154
85	regulation of cholesterol transport	9.96E-3	3 / 27
86	pattern recognition receptor signaling pathway	1.02E-2	5 / 155
87	regulation of lipid biosynthetic process	1.03E-2	4 / 79
88	regulation of phospholipase activity	1.03E-2	4 / 79
89	innate immune response-activating signal transduction	1.03E-2	5 / 157
90	negative regulation of cytokine-mediated signaling pathway	1.03E-2	3 / 28
91	growth factor binding	1.03E-2	4 / 79
92	regulation of cytokine-mediated signaling pathway	1.12E-2	4 / 81

93	activation of innate immune response	1.26E-2	5 / 165
94	response to bacterium	1.27E-2	5 / 166
95	cellular ketone metabolic process	1.27E-2	5 / 166
96	collagen metabolic process	1.35E-2	4 / 86
97	regulation of response to cytokine stimulus	1.4E-2	4 / 87
98	negative regulation of response to cytokine stimulus	1.44E-2	3 / 32
99	cellular response to interferon-gamma	1.48E-2	4 / 89
100	response to lipopolysaccharide	1.48E-2	4 / 89
101	multicellular organismal macromolecule metabolic process	1.52E-2	4 / 90
102	cellular response to alcohol	1.52E-2	3 / 33
103	sterol binding	1.64E-2	3 / 34
104	protein serine/threonine kinase activity	1.66E-2	6 / 290
105	regulation of protein serine/threonine kinase activity	1.7E-2	6 / 292
106	positive regulation of lipid biosynthetic process	1.73E-2	3 / 35
107	placenta development	1.73E-2	3 / 35
108	receptor metabolic process	1.77E-2	4 / 95
109	carboxylic acid biosynthetic process	1.79E-2	5 / 184
110	organic acid biosynthetic process	1.79E-2	5 / 184
111	multicellular organismal metabolic process	1.79E-2	4 / 96
112	positive regulation of cell-substrate adhesion	1.8E-2	3 / 36
113	positive regulation of innate immune response	1.92E-2	5 / 188
114	response to molecule of bacterial origin	2.05E-2	4 / 100
115	steroid metabolic process	2.3E-2	5 / 196
116	monocarboxylic acid binding	2.38E-2	3 / 40
117	response to interferon-gamma	2.41E-2	4 / 105
118	peptidyl-tyrosine modification	3.06E-2	5 / 210
119	peptidyl-tyrosine phosphorylation	3.06E-2	5 / 210
120	fatty acid transport	3.28E-2	3 / 45
121	regulation of steroid metabolic process	3.47E-2	3 / 46
122	regulation of fatty acid metabolic process	4.15E-2	3 / 49
123	cellular response to sterol	4.29E-2	2 / 10
124	cholesterol homeostasis	4.29E-2	3 / 50
125	MAP kinase activity	4.29E-2	2 / 10
126	3'-UTR-mediated mRNA stabilization	4.29E-2	2 / 10
127	sterol homeostasis	4.29E-2	3 / 50
128	alcohol binding	4.42E-2	3 / 51
129	arachidonic acid metabolic process	4.42E-2	3 / 51
130	positive regulation of cholesterol transport	4.89E-2	2 / 11
131	T-helper 17 type immune response	4.89E-2	2 / 11
132	regulation of sequestering of triglyceride	4.89E-2	2 / 11
133	regulation of toll-like receptor 4 signaling pathway	4.89E-2	2 / 11
134	positive regulation of sterol transport	4.89E-2	2 / 11
135	peptidyl-glutamic acid carboxylation	4.89E-2	2 / 11
136	protein carboxylation	4.89E-2	2 / 11
137	cholesterol transport	5.17E-2	3 / 55
138	sterol transport	5.17E-2	3 / 55
139	regulation of striated muscle tissue development	5.41E-2	3 / 56
140	histone kinase activity	5.49E-2	2 / 12
141	regulation of muscle tissue development	5.49E-2	3 / 57

142	regulation of triglyceride biosynthetic process	5.49E-2	2 / 12
143	regulation of cholesterol metabolic process	5.49E-2	2 / 12
144	regulation of nitric-oxide synthase biosynthetic process	5.49E-2	2 / 12
145	nitric-oxide synthase biosynthetic process	5.49E-2	2 / 12
146	regulation of muscle organ development	5.71E-2	3 / 58
147	regulation of inflammatory response	5.75E-2	4 / 140
148	embryonic organ development	6.27E-2	4 / 144
149	retinoic acid receptor binding	6.27E-2	2 / 13
150	circadian regulation of gene expression	6.27E-2	2 / 13
151	positive regulation of phospholipase C activity	6.72E-2	3 / 62
152	regulation of phospholipase C activity	7E-2	3 / 63
153	sequestering of triglyceride	7.01E-2	2 / 14
154	positive regulation of triglyceride metabolic process	7.01E-2	2 / 14
155	MAP kinase kinase activity	7.01E-2	2 / 14
156	regulation of cholesterol efflux	7.01E-2	2 / 14
157	toll-like receptor 5 signaling pathway	7.33E-2	3 / 65
158	toll-like receptor 10 signaling pathway	7.33E-2	3 / 65
159	collagen catabolic process	7.33E-2	3 / 65
160	neurotrophin TRK receptor signaling pathway	7.7E-2	5 / 274
161	neurotrophin signaling pathway	8.03E-2	5 / 277
162	icosanoid metabolic process	8.46E-2	3 / 69
163	cholesterol metabolic process	8.46E-2	3 / 69
164	fatty acid derivative metabolic process	8.46E-2	3 / 69
165	toll-like receptor TLR6:TLR2 signaling pathway	8.98E-2	3 / 71
166	toll-like receptor TLR1:TLR2 signaling pathway	8.98E-2	3 / 71
167	endopeptidase activity	8.98E-2	4 / 163
168	multicellular organismal catabolic process	8.98E-2	3 / 71
169	Fc-epsilon receptor signaling pathway	9.4E-2	4 / 166
170	fibroblast growth factor binding	9.4E-2	2 / 17
171	toll-like receptor 9 signaling pathway	9.4E-2	3 / 73
172	histone phosphorylation	9.4E-2	2 / 17
173	cellular response to biotic stimulus	9.4E-2	3 / 73
174	response to sterol	9.4E-2	2 / 17
175	long-chain fatty acid metabolic process	9.99E-2	3 / 75
176	regulation of cell-substrate adhesion	9.99E-2	3 / 75
177	response to alcohol	9.99E-2	3 / 75

Interactions

Gene 1	Gene 2	Weight	Network group	Networks
MMP12	MMP3	0.043236750178039074	Co-expression	Arijs-Rutgeerts-2009<br>Boldrick-Relman-2002<br>Ramaswamy-Golub-2001
RXRB	CDK9	0.03211466781795025	Co-expression	Alizadeh-Staudt-2000<br>Boldrick-Relman-2002
NR1D2	NR2C2	0.03163459897041321	Co-expression	Ramaswamy-Golub-2001
KDR	FLT1	0.030316736549139023	Co-expression	Arijs-Rutgeerts-2009<br>Roth-Zlotnik-2006
NR1D1	FGFR2	0.025683493353426456	Co-expression	Alizadeh-Staudt-2000<br>Boldrick-Relman-2002
NR1D2	NR1H2	0.02265908569097519	Co-expression	Rieger-Chu-2004
NR1H3	PPARG	0.022533847019076347	Co-expression	Gysin-McMahon-2012<br>Roth-Zlotnik-2006
NR1D2	GSK3B	0.021795935928821564	Co-expression	Wu-Garvey-2007
EPHX2	PPARA	0.021765772253274918	Co-expression	Arijs-Rutgeerts-2009<br>Roth-Zlotnik-2006
NR1I3	NR1I2	0.021289569325745106	Co-expression	Rieger-Chu-2004<br>Roth-Zlotnik-2006
NR1I2	PPARG	0.02123275212943554	Co-expression	Kang-Willman-2010
PPARG	THRB	0.021227585151791573	Co-expression	Kang-Willman-2010
MAPK10	PIK3CG	0.020742544904351234	Co-expression	Alizadeh-Staudt-2000
RXRB	NR1H2	0.01992536336183548	Co-expression	Gysin-McMahon-2012
RARG	FGFR2	0.019609088078141212	Co-expression	Ramaswamy-Golub-2001
RARG	JAK2	0.01916118524968624	Co-expression	Gysin-McMahon-2012
MAPKAPK2	NR1H2	0.0183554720133543	Co-expression	Wu-Garvey-2007
MAPK14	LTA4H	0.018249066546559334	Co-expression	Roth-Zlotnik-2006
RXRB	MAPK14	0.017710430547595024	Co-expression	Alizadeh-Staudt-2000
MAPK10	MMP12	0.01747087761759758	Co-expression	Wu-Garvey-2007
ESRRG	RORA	0.017252985388040543	Co-expression	Cheok-Evans-2003
MAPK10	FGFR1	0.017057158052921295	Co-expression	Gysin-McMahon-2012
RORC	PPARD	0.017010711133480072	Co-expression	Wu-Garvey-2007
NR2C2	MAPK10	0.01682709902524948	Co-expression	Wu-Garvey-2007
CDK9	EPHX2	0.01647859998047352	Co-expression	Rieger-Chu-2004
PLA2G2A	MMP3	0.01639402285218239	Co-expression	Alizadeh-Staudt-2000
DPYD	MMP13	0.016266196966171265	Co-expression	Ramaswamy-Golub-2001
PIK3CG	PPARG	0.016093742102384567	Co-expression	Wu-Garvey-2007
PGR	FGFR2	0.014344998635351658	Co-expression	Ramaswamy-Golub-2001
MAPK14	CDK9	0.014261950738728046	Co-expression	Ramaswamy-Golub-2001
RARB	MAPK10	0.014128590933978558	Co-expression	Gysin-McMahon-2012
CA1	THRB	0.014084487222135067	Co-expression	Arijs-Rutgeerts-2009
KDR	REN	0.013823027722537518	Co-expression	Smirnov-Cheung-2009
ESRRA	MAPKAPK2	0.01348874345421791	Co-expression	Wu-Garvey-2007
MAPK10	F2	0.013400968164205551	Co-expression	Ramaswamy-Golub-2001
MAPK10	F10	0.012979976832866669	Co-expression	Gysin-McMahon-2012
NR1I3	NR1H2	0.01281662192195654	Co-expression	Perou-Botstein-1999
NR1D2	MAPKAPK2	0.012671624310314655	Co-expression	Kang-Willman-2010
NR1H3	MMP12	0.012486720457673073	Co-expression	Ramaswamy-Golub-2001
RORC	NR1I3	0.01219329982995987	Co-expression	Roth-Zlotnik-2006
CDK9	PIK3CG	0.012154494412243366	Co-expression	Cheok-Evans-2003
FLT1	THRB	0.012036114931106567	Co-expression	Ramaswamy-Golub-2001
RARB	ESRRA	0.011722870171070099	Co-expression	Cheok-Evans-2003
F2	MMP3	0.011672643013298512	Co-expression	Kang-Willman-2010
PIK3CG	NR1H4	0.011357635259628296	Co-expression	Ramaswamy-Golub-2001
KDR	FGFR1	0.011164223775267601	Co-expression	Alizadeh-Staudt-2000
NR1H3	PLA2G2A	0.010994229465723038	Co-expression	Alizadeh-Staudt-2000

				Boldrick-Relman-2002
NR2C1	PIK3CG	0.010694886557757854	Co-expression	Ramaswamy-Golub-2001
ESR1	KDR	0.010648923926055431	Co-expression	Alizadeh-Staudt-2000
KDR	MMP3	0.010643739253282547	Co-expression	Alizadeh-Staudt-2000
RXRB	DHODH	0.010622811503708363	Co-expression	Perou-Botstein-1999
ESRRG	DPYD	0.009727969765663147	Co-expression	Arijs-Rutgeerts-2009
CDK2	GSK3B	0.009701027534902096	Co-expression	Alizadeh-Staudt-2000
KDR	PPARD	0.009465987794101238	Co-expression	Perou-Botstein-1999
NR1I2	MMP13	0.009203195571899414	Co-expression	Wu-Garvey-2007
NR1H3	REN	0.008947978727519512	Co-expression	Rieger-Chu-2004
CDK2	MAPK14	0.008813655935227871	Co-expression	Alizadeh-Staudt-2000
ESRRA	EPHX2	0.008711691945791245	Co-expression	Arijs-Rutgeerts-2009
PGR	MMP12	0.008544520009309053	Co-expression	Kang-Willman-2010<br>Wu-Garvey-2007
NR2C2	ESR1	0.008530636318027973	Co-expression	Perou-Botstein-1999
ESR1	CA1	0.008343247696757317	Co-expression	Gysin-McMahon-2012
NR1I2	EPHX2	0.0082744425162673	Co-expression	Arijs-Rutgeerts-2009
MAPKAPK2	ESR1	0.008247756399214268	Co-expression	Perou-Botstein-1999
KDR	F10	0.008109210059046745	Co-expression	Perou-Botstein-1999
PGR	KDR	0.007630572654306889	Co-expression	Wu-Garvey-2007
F10	FGFR2	0.007585418876260519	Co-expression	Wu-Garvey-2007
PLA2G2A	F10	0.007522277068346739	Co-expression	Wu-Garvey-2007
NR1I2	F2	0.007515043020248413	Co-expression	Roth-Zlotnik-2006
REN	CA1	0.007287074811756611	Co-expression	Rieger-Chu-2004
MAPK14	PIK3CG	0.007059122435748577	Co-expression	Boldrick-Relman-2002
PGR	MAPK10	0.006829873658716679	Co-expression	Wu-Garvey-2007
F2	F10	0.006107615306973457	Co-expression	Roth-Zlotnik-2006
CDK9	FGFR2	0.006060219369828701	Co-expression	Boldrick-Relman-2002
NR1I2	REN	0.005140224471688271	Co-expression	Rieger-Chu-2004
NR1I3	F2	0.005070996005088091	Co-expression	Roth-Zlotnik-2006
EPHX2	LTA4H	0.004831915255635977	Co-expression	Arijs-Rutgeerts-2009
PGR	NR2C2	0.0046678646467626095	Co-expression	Wu-Garvey-2007
ESR2	MMP12	0.004125125706195831	Co-expression	Kang-Willman-2010
NR1H3	KDR	0.004100181628018618	Co-expression	Ramaswamy-Golub-2001
PGR	MMP3	0.0038916515186429024	Co-expression	Wu-Garvey-2007
NR1H3	DHODH	0.0038481594529002905	Co-expression	Boldrick-Relman-2002
NR1D1	NR1H3	0.0037995490711182356	Co-expression	Boldrick-Relman-2002
MMP12	PPARG	0.0014438159996643662	Co-expression	Kang-Willman-2010
MMP12	MMP13	0.0012650575954467058	Co-expression	Kang-Willman-2010
PGR	MMP3	0.02585810050368309	Co-localization	Johnson-Shoemaker-2003
PGR	ESR1	0.022514594718813896	Co-localization	Johnson-Shoemaker-2003
NR2C2	GSK3B	0.02153736539185047	Co-localization	Johnson-Shoemaker-2003
RORA	NR1H4	0.020591670647263527	Co-localization	Johnson-Shoemaker-2003
ESR1	MMP3	0.01997765526175499	Co-localization	Johnson-Shoemaker-2003
NR1H3	F10	0.019330978393554688	Co-localization	Johnson-Shoemaker-2003
PLA2G2A	MMP12	0.014045377261936665	Co-localization	Johnson-Shoemaker-2003
ESR1	REN	0.013943271711468697	Co-localization	Schadt-Shoemaker-2004
ESR1	F10	0.010013038292527199	Co-localization	Schadt-Shoemaker-2004
PLA2G2A	F10	0.007618853822350502	Co-localization	Schadt-Shoemaker-2004
ESRRA	PPARA	0.025752108544111252	Genetic interactions	Lin-Smith-2010
RORC	REN	0.006957837380468845	Genetic interactions	Lin-Smith-2010
RORC	MAPKAPK2	0.006427282001823187	Genetic interactions	Lin-Smith-2010
RARG	FGFR1	0.00400179298594594	Genetic interactions	Lin-Smith-2010
PLA2G2A	CA1	0.0032838573679327965	Genetic interactions	Lin-Smith-2010
ESR2	RXRB	0.003167258109897375	Genetic interactions	Lin-Smith-2010
KDR	DHODH	0.0026649069041013718	Genetic interactions	Lin-Smith-2010
GSK3B	PPARA	0.002508551348000765	Genetic interactions	Lin-Smith-2010

PIK3CG	NR1H2	0.0024490407668054104	Genetic interactions	Lin-Smith-2010
GSK3B	FGFR1	0.00235161860473454	Genetic interactions	Lin-Smith-2010
KDR	F10	0.0022838159929960966	Genetic interactions	Lin-Smith-2010
PPARA	NR1H4	0.0022201472893357277	Genetic interactions	Lin-Smith-2010
REN	NR1H2	0.002202487550675869	Genetic interactions	Lin-Smith-2010
NR2C1	GSK3B	0.002052635420113802	Genetic interactions	Lin-Smith-2010
CDK9	NR1H2	0.0020062513649463654	Genetic interactions	Lin-Smith-2010
KDR	F2	0.0019962212536484003	Genetic interactions	Lin-Smith-2010
MAPK10	F2	0.0019184473203495145	Genetic interactions	Lin-Smith-2010
NR1H3	FGFR2	0.0018740467494353652	Genetic interactions	Lin-Smith-2010
NR2C1	NR1H2	0.0018494734540581703	Genetic interactions	Lin-Smith-2010
RARB	PLA2G2A	0.0017488953890278935	Genetic interactions	Lin-Smith-2010
NR1D2	RXRB	0.0016960856737568974	Genetic interactions	Lin-Smith-2010
NR1I2	MAPKAPK2	0.0014395348262041807	Genetic interactions	Lin-Smith-2010
LTA4H	PPARD	0.0012460262514650822	Genetic interactions	Lin-Smith-2010
NR1I2	PPARD	0.00120081240311265	Genetic interactions	Lin-Smith-2010
NR1D2	FGFR1	0.0011410964652895927	Genetic interactions	Lin-Smith-2010
MAPK10	NR1H4	0.001121930661611259	Genetic interactions	Lin-Smith-2010
GSK3B	FLT1	0.0010738756973296404	Genetic interactions	Lin-Smith-2010
PIK3CG	PPARG	0.0010691799689084291	Genetic interactions	Lin-Smith-2010
MMP3	PPARD	0.001047093071974814	Genetic interactions	Lin-Smith-2010
RARB	RXRG	0.0010351411765441298	Genetic interactions	Lin-Smith-2010
KDR	MMP13	0.001004209159873426	Genetic interactions	Lin-Smith-2010
DPYD	PLA2G2A	9.879310382530093E-4	Genetic interactions	Lin-Smith-2010
ESR2	FLT1	9.730724850669503E-4	Genetic interactions	Lin-Smith-2010
NR1D1	CDK9	9.660786017775536E-4	Genetic interactions	Lin-Smith-2010
MMP13	PPARG	9.185090311802924E-4	Genetic interactions	Lin-Smith-2010
THRB	NR1H4	9.121192852035165E-4	Genetic interactions	Lin-Smith-2010
FLT1	PPARA	8.782998775132E-4	Genetic interactions	Lin-Smith-2010
DPYD	PPARA	8.520803530700505E-4	Genetic interactions	Lin-Smith-2010
ESRRG	GSK3B	8.354241726920009E-4	Genetic interactions	Lin-Smith-2010
REN	THRB	8.213643450289965E-4	Genetic interactions	Lin-Smith-2010
NR1I2	MAPK10	8.081633131951094E-4	Genetic interactions	Lin-Smith-2010
NR2C1	PPARG	8.07426346000284E-4	Genetic interactions	Lin-Smith-2010
ESRRG	PLA2G2A	7.922128424979746E-4	Genetic interactions	Lin-Smith-2010
MAPK10	PPARD	7.784959161654115E-4	Genetic interactions	Lin-Smith-2010
ESRRG	JAK2	7.748457137495279E-4	Genetic interactions	Lin-Smith-2010
RORA	JAK2	7.618249510414898E-4	Genetic interactions	Lin-Smith-2010
ESRRG	NR1H2	7.527371635660529E-4	Genetic interactions	Lin-Smith-2010
MAPK14	EPHX2	7.498431950807571E-4	Genetic interactions	Lin-Smith-2010
ESRRG	NR1H4	7.393768173642457E-4	Genetic interactions	Lin-Smith-2010
ESR1	KDR	7.392543484456837E-4	Genetic interactions	Lin-Smith-2010
THRA	FGFR2	7.299561984837055E-4	Genetic interactions	Lin-Smith-2010
PIK3CG	FGFR2	7.140024681575596E-4	Genetic interactions	Lin-Smith-2010
RARB	MAPK14	6.774006760679185E-4	Genetic interactions	Lin-Smith-2010
NR1D2	NR1D1	6.457331473939121E-4	Genetic interactions	Lin-Smith-2010
ESRRG	FGFR1	6.405303720384836E-4	Genetic interactions	Lin-Smith-2010
ESRRG	MMP13	6.360117695294321E-4	Genetic interactions	Lin-Smith-2010
NR2C2	MAPK10	6.276679341681302E-4	Genetic interactions	Lin-Smith-2010
NR1D2	MAPK10	6.151240668259561E-4	Genetic interactions	Lin-Smith-2010
RORA	CDK9	5.962959257885814E-4	Genetic interactions	Lin-Smith-2010
RXRG	THRB	5.784468376077712E-4	Genetic interactions	Lin-Smith-2010
EPHX2	FGFR2	5.362827214412391E-4	Genetic interactions	Lin-Smith-2010
LTA4H	FGFR2	5.329888663254678E-4	Genetic interactions	Lin-Smith-2010
NR1D2	FLT1	5.210860981605947E-4	Genetic interactions	Lin-Smith-2010
ESRRG	PPARD	5.130458157509565E-4	Genetic interactions	Lin-Smith-2010
MAPK10	PPARG	4.986528656445444E-4	Genetic interactions	Lin-Smith-2010

RARB	RORA	4.939031205140054E-4	Genetic interactions	Lin-Smith-2010
ESRRG	ESR1	4.682037397287786E-4	Genetic interactions	Lin-Smith-2010
NR1D1	FLT1	4.659265687223524E-4	Genetic interactions	Lin-Smith-2010
RXRG	FGFR2	4.52214851975441E-4	Genetic interactions	Lin-Smith-2010
MAPK10	THRB	4.259571142029017E-4	Genetic interactions	Lin-Smith-2010
DPYD	PPARG	4.0981030906550586E-4	Genetic interactions	Lin-Smith-2010
NR2C2	RORA	4.0669579175300896E-4	Genetic interactions	Lin-Smith-2010
NR1D2	RORA	3.985680523328483E-4	Genetic interactions	Lin-Smith-2010
MAPK14	FLT1	3.9443071000277996E-4	Genetic interactions	Lin-Smith-2010
DPYD	MAPK14	3.826559113804251E-4	Genetic interactions	Lin-Smith-2010
MAPK14	THRB	3.785380395129323E-4	Genetic interactions	Lin-Smith-2010
FLT1	THRB	3.608383412938565E-4	Genetic interactions	Lin-Smith-2010
DPYD	THRB	3.500663733575493E-4	Genetic interactions	Lin-Smith-2010
ESRRG	MAPK10	3.4528691321611404E-4	Genetic interactions	Lin-Smith-2010
RORA	MAPK10	3.3948459895327687E-4	Genetic interactions	Lin-Smith-2010
MAPK10	FGFR2	3.3300233189947903E-4	Genetic interactions	Lin-Smith-2010
RORA	PPARG	3.2310085953213274E-4	Genetic interactions	Lin-Smith-2010
FGFR2	PPARG	3.1693142955191433E-4	Genetic interactions	Lin-Smith-2010
RORA	MAPK14	3.0169193632900715E-4	Genetic interactions	Lin-Smith-2010
MAPK14	FGFR2	2.9593126964755356E-4	Genetic interactions	Lin-Smith-2010
RORA	FLT1	2.875853970181197E-4	Genetic interactions	Lin-Smith-2010
ESRRG	DPYD	2.8376877889968455E-4	Genetic interactions	Lin-Smith-2010
RORA	THRB	2.75997823337093E-4	Genetic interactions	Lin-Smith-2010
FGFR2	THRB	2.70727789029479E-4	Genetic interactions	Lin-Smith-2010
ESRRG	RORA	2.2372775129042566E-4	Genetic interactions	Lin-Smith-2010
ESRRG	FGFR2	2.1945580374449492E-4	Genetic interactions	Lin-Smith-2010
RORA	NR1D1	0.39692720770835876	Pathway	PATHWAYCOMMONS-REACTOME
RXRG	NR1H2	0.18449123203754425	Pathway	Wu-Stein-2010
RXRG	NR1H4	0.16041384637355804	Pathway	Wu-Stein-2010
RXRG	NR1H3	0.1473913937807083	Pathway	Wu-Stein-2010
F2	F10	0.1373298577964306	Pathway	PATHWAYCOMMONS-REACTOME<br>Wu-Stein-2010
RARG	THRA	0.0932689756155014	Pathway	Wu-Stein-2010
RARG	THRB	0.08184022456407547	Pathway	Wu-Stein-2010
RARB	THRA	0.08164875954389572	Pathway	Wu-Stein-2010
RARB	THRB	0.0716438964009285	Pathway	Wu-Stein-2010
RXRB	NR1H2	0.055716972798109055	Pathway	Wu-Stein-2010
RXRB	NR1H4	0.04844552278518677	Pathway	Wu-Stein-2010
NR1H3	PPARG	0.045004721730947495	Pathway	Wu-Stein-2010
RXRB	NR1H3	0.04451269656419754	Pathway	Wu-Stein-2010
ESRRA	PPARA	0.043132755905389786	Pathway	Wu-Stein-2010
NR1H3	PPARA	0.03913494199514389	Pathway	Wu-Stein-2010
MAPK14	GSK3B	0.03789595514535904	Pathway	PATHWAYCOMMONS-NCI_NATURE<br>Wu-Stein-2010
KDR	FLT1	0.03713475726544857	Pathway	PATHWAYCOMMONS-NCI_NATURE<br>Wu-Stein-2010
CDK2	MAPK14	0.032336439937353134	Pathway	PATHWAYCOMMONS-NCI_NATURE
RXRG	PPARD	0.031032348051667213	Pathway	Wu-Stein-2010
RARG	RXRG	0.02518633008003235	Pathway	Wu-Stein-2010
NR1D1	PPARG	0.02288435585796833	Pathway	Wu-Stein-2010
RARB	RXRG	0.022048410028219223	Pathway	Wu-Stein-2010
RXRG	PPARG	0.015684736892580986	Pathway	Wu-Stein-2010
RXRG	PPARA	0.013639041222631931	Pathway	Wu-Stein-2010

ESR2	PPARG	0.012165921740233898	Pathway	Wu-Stein-2010
ESR2	ESR1	0.01215824019163847	Pathway	PATHWAYCOMMONS-NCI_NATURE
RARB	RARG	0.010810628533363342	Pathway	Wu-Stein-2010
FGFR2	FGFR1	0.010412666946649551	Pathway	Wu-Stein-2010
RXRB	PPARD	0.00937187485396862	Pathway	Wu-Stein-2010
PIK3CG	FLT1	0.008706091903150082	Pathway	Wu-Stein-2010
RARG	RXRB	0.007606356870383024	Pathway	Wu-Stein-2010
KDR	PIK3CG	0.007380048278719187	Pathway	Wu-Stein-2010
PIK3CG	FGFR2	0.007137341424822807	Pathway	Wu-Stein-2010
PIK3CG	FGFR1	0.006896982900798321	Pathway	Wu-Stein-2010
RARB	PPARG	0.006732297129929066	Pathway	Wu-Stein-2010
MAPKAPK2	MAPK14	0.006674602162092924	Pathway	Wu-Stein-2010
RARB	RXRB	0.006658694241195917	Pathway	Wu-Stein-2010
PGR	MAPK14	0.006296984385699034	Pathway	Wu-Stein-2010
RXRB	PPARG	0.004736843518912792	Pathway	Wu-Stein-2010
F2	PPARA	0.004600238054990768	Pathway	Wu-Stein-2010
ESR1	PPARG	0.004191082902252674	Pathway	Wu-Stein-2010
RXRB	PPARA	0.004119036719202995	Pathway	Wu-Stein-2010
PIK3CG	JAK2	0.004011406097561121	Pathway	Wu-Stein-2010
RARG	MAPK14	0.0033220662735402584	Pathway	Wu-Stein-2010
MAPK14	KDR	0.003208716632798314	Pathway	Wu-Stein-2010
RARB	MAPK14	0.0029081758111715317	Pathway	Wu-Stein-2010
MAPK14	F2	0.002285235794261098	Pathway	Wu-Stein-2010
MAPK14	ESR1	0.0018104378832504153	Pathway	Wu-Stein-2010
MAPK14	JAK2	0.0017440896481275558	Pathway	Wu-Stein-2010
NR2C2	NR2C1	0.465468168258667	Physical interactions	IREF-OPHID
MAPK10	DHODH	0.4099876582622528	Physical interactions	IREF-OPHID
CDK9	FGFR1	0.29400432109832764	Physical interactions	Varjosalo-Gstaiger-2013 A
RARB	RXRG	0.2602044343948364	Physical interactions	IREF-OPHID
RXRB	NR1H2	0.2161848545074463	Physical interactions	IREF-OPHID
RARB	RXRB	0.2073972523212433	Physical interactions	IREF-OPHID
RXRB	NR1I2	0.2013409435749054	Physical interactions	IREF-OPHID
MAPKAPK2	MAPK14	0.1811496615409851	Physical interactions	IREF-OPHID<br>Varjosalo-Gstaiger-2013 A
KDR	FLT1	0.09555456042289734	Physical interactions	IREF-OPHID
RXRG	THRB	0.07476211339235306	Physical interactions	IREF-OPHID
RXRG	PPARA	0.06852563470602036	Physical interactions	IREF-OPHID
RXRG	PPARG	0.06532465666532516	Physical interactions	IREF-OPHID
CDK2	ESR1	0.06213697791099548	Physical interactions	Cheng-Chen-2010
RXRB	THRA	0.06198616698384285	Physical interactions	IREF-OPHID
RXRB	THRB	0.05958951637148857	Physical interactions	IREF-OPHID
NR2C2	ESR1	0.05004659295082092	Physical interactions	IREF-OPHID
NR2C1	ESR1	0.042015619575977325	Physical interactions	IREF-OPHID
NR1H3	PPARA	0.03901902586221695	Physical interactions	IREF-OPHID
NR1H3	CDK2	0.01953577995300293	Physical interactions	IREF-OPHID
ESR2	ESR1	0.011904633603990078	Physical interactions	IREF-OPHID
MAPKAPK2	MAPK14	1.9786877632141113	Predicted	I2D-BioGRID-Fly2Human<br>I2D-IntAct-Fly2Human<br>I2D-MINT-Fly2Human
MAPK10	DHODH	1.8117722272872925	Predicted	I2D-BioGRID-Fly2Human<br>I2D-IntAct-Fly2Human<br>I2D-MINT-Fly2Human
DPYD	DHODH	0.8162329792976379	Shared protein domains	INTERPRO<br>PFAM
MMP12	MMP13	0.0866105854511261	Shared protein domains	INTERPRO<br>PFAM
MMP3	MMP13	0.08632494136691093	Shared protein domains	INTERPRO

				PFAM
MMP12	MMP3	0.08617567270994186	Shared protein domains	INTERPRO PFAM
FGFR2	FGFR1	0.07727719098329544	Shared protein domains	INTERPRO PFAM
PPARA	PPARD	0.06783170998096466	Shared protein domains	INTERPRO PFAM
NR1H3	NR1H2	0.0676528736948967	Shared protein domains	INTERPRO PFAM
RORC	RORA	0.0590713769197464	Shared protein domains	INTERPRO PFAM
NR1H3	NR1H4	0.058772098273038864	Shared protein domains	INTERPRO PFAM
RARB	RARG	0.05795972794294357	Shared protein domains	INTERPRO PFAM
NR1H2	NR1H4	0.05715620517730713	Shared protein domains	INTERPRO PFAM
PPARG	PPARD	0.055352840572595596	Shared protein domains	INTERPRO PFAM
PPARG	PPARA	0.0553528256714344	Shared protein domains	INTERPRO PFAM
THRA	NR1H2	0.052789557725191116	Shared protein domains	INTERPRO PFAM
RXRG	RXRB	0.05268229357898235	Shared protein domains	INTERPRO PFAM
RARG	RXRB	0.05182567797601223	Shared protein domains	INTERPRO PFAM
RARB	THRA	0.0512840561568737	Shared protein domains	INTERPRO PFAM
RARB	NR1H2	0.05060655251145363	Shared protein domains	INTERPRO PFAM
RARB	RXRB	0.05018881894648075	Shared protein domains	INTERPRO PFAM
NR1I2	THRB	0.04945397190749645	Shared protein domains	INTERPRO PFAM
NR1I3	THRB	0.04945397190749645	Shared protein domains	INTERPRO PFAM
NR1I3	NR1I2	0.04945397190749645	Shared protein domains	INTERPRO PFAM
THRB	NR1H4	0.04901903308928013	Shared protein domains	INTERPRO PFAM
NR1I2	NR1H4	0.04901903308928013	Shared protein domains	INTERPRO PFAM
NR1I3	NR1H4	0.04901903308928013	Shared protein domains	INTERPRO PFAM
RORA	THRB	0.04901314526796341	Shared protein domains	INTERPRO PFAM
RORA	NR1I2	0.04901314526796341	Shared protein domains	INTERPRO PFAM
RORA	NR1I3	0.04901314526796341	Shared protein domains	INTERPRO PFAM
RORC	THRB	0.04901314526796341	Shared protein domains	INTERPRO PFAM
RORC	NR1I2	0.04901314526796341	Shared protein domains	INTERPRO PFAM
RORC	NR1I3	0.04901314526796341	Shared protein domains	INTERPRO PFAM
NR1H3	THRB	0.04878928139805794	Shared protein domains	INTERPRO PFAM
NR1I2	NR1H3	0.04878928139805794	Shared protein domains	INTERPRO PFAM
NR1I3	NR1H3	0.04878928139805794	Shared protein domains	INTERPRO

				PFAM
RORA	NR1H4	0.0485851988196373	Shared protein domains	INTERPRO PFAM
RORC	NR1H4	0.0485851988196373	Shared protein domains	INTERPRO PFAM
RORA	NR1H3	0.04835914261639118	Shared protein domains	INTERPRO PFAM
RORC	NR1H3	0.04835914261639118	Shared protein domains	INTERPRO PFAM
THRA	THRB	0.04821889474987984	Shared protein domains	INTERPRO PFAM
THRA	NR1I2	0.04821889474987984	Shared protein domains	INTERPRO PFAM
NR1I3	THRA	0.04821889474987984	Shared protein domains	INTERPRO PFAM
ESRRG	THRA	0.04807572811841965	Shared protein domains	INTERPRO PFAM
THRA	NR1H4	0.04780355095863342	Shared protein domains	INTERPRO PFAM
RORA	THRA	0.047797929495573044	Shared protein domains	INTERPRO PFAM
RORC	THRA	0.047797929495573044	Shared protein domains	INTERPRO PFAM
ESRRG	RARG	0.047633757814764977	Shared protein domains	INTERPRO PFAM
NR1H2	THRB	0.04761234484612942	Shared protein domains	INTERPRO PFAM
NR1I2	NR1H2	0.04761234484612942	Shared protein domains	INTERPRO PFAM
NR1I3	NR1H2	0.04761234484612942	Shared protein domains	INTERPRO PFAM
THRA	NR1H3	0.04758414998650551	Shared protein domains	INTERPRO PFAM
ESRRG	RXRB	0.04753287136554718	Shared protein domains	INTERPRO PFAM
ESRRG	NR1H2	0.04747248813509941	Shared protein domains	INTERPRO PFAM
RORA	NR1H2	0.047201132401824	Shared protein domains	INTERPRO PFAM
RORC	NR1H2	0.047201132401824	Shared protein domains	INTERPRO PFAM
RARB	THRB	0.04636017791926861	Shared protein domains	INTERPRO PFAM
RARB	NR1I2	0.04636017791926861	Shared protein domains	INTERPRO PFAM
RARB	NR1I3	0.04636017791926861	Shared protein domains	INTERPRO PFAM
FLT1	FGFR1	0.046316420659422874	Shared protein domains	INTERPRO PFAM
FLT1	FGFR2	0.046316420659422874	Shared protein domains	INTERPRO PFAM
ESRRG	RARB	0.04622715339064598	Shared protein domains	INTERPRO PFAM
RARG	THRA	0.04609023965895176	Shared protein domains	INTERPRO PFAM
RARG	RXRG	0.04608701542019844	Shared protein domains	INTERPRO PFAM
RXRB	THRA	0.0459954347461462	Shared protein domains	INTERPRO PFAM
RARB	NR1H4	0.04597432538866997	Shared protein domains	INTERPRO PFAM
RARB	RORA	0.045969102531671524	Shared protein domains	INTERPRO PFAM

RARB	RORC	0.045969102531671524	Shared protein domains	INTERPRO PFAM
RARB	NR1H3	0.04577050171792507	Shared protein domains	INTERPRO PFAM
RARG	NR1H2	0.04553300328552723	Shared protein domains	INTERPRO PFAM
RXRB	NR1H2	0.045440396293997765	Shared protein domains	INTERPRO PFAM
RARB	RXRG	0.04474376514554024	Shared protein domains	INTERPRO PFAM
KDR	FGFR1	0.04466992802917957	Shared protein domains	INTERPRO PFAM
KDR	FGFR2	0.04466992802917957	Shared protein domains	INTERPRO PFAM
NR2C1	NR1D1	0.044550810009241104	Shared protein domains	INTERPRO PFAM
NR2C2	NR1D1	0.044550810009241104	Shared protein domains	INTERPRO PFAM
NR2C2	NR2C1	0.044550810009241104	Shared protein domains	INTERPRO PFAM
ESRRA	NR1D1	0.044550810009241104	Shared protein domains	INTERPRO PFAM
ESRRA	NR2C1	0.044550810009241104	Shared protein domains	INTERPRO PFAM
ESRRA	NR2C2	0.044550810009241104	Shared protein domains	INTERPRO PFAM
NR1D2	NR1D1	0.044550810009241104	Shared protein domains	INTERPRO PFAM
NR1D2	NR2C1	0.044550810009241104	Shared protein domains	INTERPRO PFAM
NR1D2	NR2C2	0.044550810009241104	Shared protein domains	INTERPRO PFAM
NR1D2	ESRRA	0.044550810009241104	Shared protein domains	INTERPRO PFAM
NR1D1	ESR1	0.04455075040459633	Shared protein domains	INTERPRO PFAM
NR2C1	ESR1	0.04455075040459633	Shared protein domains	INTERPRO PFAM
NR2C2	ESR1	0.04455075040459633	Shared protein domains	INTERPRO PFAM
ESRRA	ESR1	0.04455075040459633	Shared protein domains	INTERPRO PFAM
NR1D2	ESR1	0.04455075040459633	Shared protein domains	INTERPRO PFAM
PGR	NR1D1	0.04455075040459633	Shared protein domains	INTERPRO PFAM
PGR	NR2C1	0.04455075040459633	Shared protein domains	INTERPRO PFAM
PGR	NR2C2	0.04455075040459633	Shared protein domains	INTERPRO PFAM
PGR	ESRRA	0.04455075040459633	Shared protein domains	INTERPRO PFAM
PGR	NR1D2	0.04455075040459633	Shared protein domains	INTERPRO PFAM
ESR2	NR1D1	0.04455075040459633	Shared protein domains	INTERPRO PFAM
ESR2	NR2C1	0.04455075040459633	Shared protein domains	INTERPRO PFAM
ESR2	NR2C2	0.04455075040459633	Shared protein domains	INTERPRO PFAM
ESR2	ESRRA	0.04455075040459633	Shared protein domains	INTERPRO PFAM

ESR2	NR1D2	0.04455075040459633	Shared protein domains	INTERPRO PFAM
PGR	ESR1	0.044550696387887	Shared protein domains	INTERPRO PFAM
ESR2	ESR1	0.044550696387887	Shared protein domains	INTERPRO PFAM
ESR2	PGR	0.044550696387887	Shared protein domains	INTERPRO PFAM
NR1D1	PPARG	0.04414699785411358	Shared protein domains	INTERPRO PFAM
NR2C1	PPARG	0.04414699785411358	Shared protein domains	INTERPRO PFAM
NR2C2	PPARG	0.04414699785411358	Shared protein domains	INTERPRO PFAM
ESRRA	PPARG	0.04414699785411358	Shared protein domains	INTERPRO PFAM
NR1D2	PPARG	0.04414699785411358	Shared protein domains	INTERPRO PFAM
ESR1	PPARG	0.04414694383740425	Shared protein domains	INTERPRO PFAM
PGR	PPARG	0.04414694383740425	Shared protein domains	INTERPRO PFAM
ESR2	PPARG	0.04414694383740425	Shared protein domains	INTERPRO PFAM
NR1D1	PPARA	0.04393242485821247	Shared protein domains	INTERPRO PFAM
NR2C1	PPARA	0.04393242485821247	Shared protein domains	INTERPRO PFAM
NR2C2	PPARA	0.04393242485821247	Shared protein domains	INTERPRO PFAM
ESRRA	PPARA	0.04393242485821247	Shared protein domains	INTERPRO PFAM
NR1D2	PPARA	0.04393242485821247	Shared protein domains	INTERPRO PFAM
NR1D1	PPARD	0.04393240436911583	Shared protein domains	INTERPRO PFAM
NR2C1	PPARD	0.04393240436911583	Shared protein domains	INTERPRO PFAM
NR2C2	PPARD	0.04393240436911583	Shared protein domains	INTERPRO PFAM
ESRRA	PPARD	0.04393240436911583	Shared protein domains	INTERPRO PFAM
NR1D2	PPARD	0.04393240436911583	Shared protein domains	INTERPRO PFAM
ESR1	PPARA	0.043932365253567696	Shared protein domains	INTERPRO PFAM
PGR	PPARA	0.043932365253567696	Shared protein domains	INTERPRO PFAM
ESR2	PPARA	0.043932365253567696	Shared protein domains	INTERPRO PFAM
ESR1	PPARD	0.0439323466271162	Shared protein domains	INTERPRO PFAM
PGR	PPARD	0.0439323466271162	Shared protein domains	INTERPRO PFAM
ESR2	PPARD	0.0439323466271162	Shared protein domains	INTERPRO PFAM
F2	F10	0.04362805373966694	Shared protein domains	INTERPRO PFAM
ESRRG	NR1D1	0.0434532705694437	Shared protein domains	INTERPRO PFAM
ESRRG	NR2C1	0.0434532705694437	Shared protein domains	INTERPRO PFAM

ESRRG	NR2C2	0.0434532705694437	Shared protein domains	INTERPRO PFAM
ESRRG	ESRRA	0.0434532705694437	Shared protein domains	INTERPRO PFAM
ESRRG	NR1D2	0.0434532705694437	Shared protein domains	INTERPRO PFAM
ESRRG	ESR1	0.04345321282744408	Shared protein domains	INTERPRO PFAM
ESRRG	PGR	0.04345321282744408	Shared protein domains	INTERPRO PFAM
ESR2	ESRRG	0.04345321282744408	Shared protein domains	INTERPRO PFAM
NR1D1	THRB	0.043111249804496765	Shared protein domains	INTERPRO PFAM
NR1D1	NR1I2	0.043111249804496765	Shared protein domains	INTERPRO PFAM
NR1D1	NR1I3	0.043111249804496765	Shared protein domains	INTERPRO PFAM
NR2C1	THRB	0.043111249804496765	Shared protein domains	INTERPRO PFAM
NR2C1	NR1I2	0.043111249804496765	Shared protein domains	INTERPRO PFAM
NR2C1	NR1I3	0.043111249804496765	Shared protein domains	INTERPRO PFAM
NR2C2	THRB	0.043111249804496765	Shared protein domains	INTERPRO PFAM
NR2C2	NR1I2	0.043111249804496765	Shared protein domains	INTERPRO PFAM
NR2C2	NR1I3	0.043111249804496765	Shared protein domains	INTERPRO PFAM
ESRRA	THRB	0.043111249804496765	Shared protein domains	INTERPRO PFAM
ESRRA	NR1I2	0.043111249804496765	Shared protein domains	INTERPRO PFAM
ESRRA	NR1I3	0.043111249804496765	Shared protein domains	INTERPRO PFAM
NR1D2	THRB	0.043111249804496765	Shared protein domains	INTERPRO PFAM
NR1D2	NR1I2	0.043111249804496765	Shared protein domains	INTERPRO PFAM
NR1D2	NR1I3	0.043111249804496765	Shared protein domains	INTERPRO PFAM
ESR1	THRB	0.04311119206249714	Shared protein domains	INTERPRO PFAM
NR1I2	ESR1	0.04311119206249714	Shared protein domains	INTERPRO PFAM
NR1I3	ESR1	0.04311119206249714	Shared protein domains	INTERPRO PFAM
PGR	THRB	0.04311119206249714	Shared protein domains	INTERPRO PFAM
PGR	NR1I2	0.04311119206249714	Shared protein domains	INTERPRO PFAM
PGR	NR1I3	0.04311119206249714	Shared protein domains	INTERPRO PFAM
ESR2	THRB	0.04311119206249714	Shared protein domains	INTERPRO PFAM
ESR2	NR1I2	0.04311119206249714	Shared protein domains	INTERPRO PFAM
ESR2	NR1I3	0.04311119206249714	Shared protein domains	INTERPRO PFAM
ESRRG	PPARG	0.04306914657354355	Shared protein domains	INTERPRO PFAM

ESRRG	PPARA	0.04286503791809082	Shared protein domains	INTERPRO PFAM
ESRRG	PPARD	0.04286501742899418	Shared protein domains	INTERPRO PFAM
NR1D1	NR1H4	0.042776890099048615	Shared protein domains	INTERPRO PFAM
NR2C1	NR1H4	0.042776890099048615	Shared protein domains	INTERPRO PFAM
NR2C2	NR1H4	0.042776890099048615	Shared protein domains	INTERPRO PFAM
ESRRA	NR1H4	0.042776890099048615	Shared protein domains	INTERPRO PFAM
NR1D2	NR1H4	0.042776890099048615	Shared protein domains	INTERPRO PFAM
ESR1	NR1H4	0.04277683235704899	Shared protein domains	INTERPRO PFAM
PGR	NR1H4	0.04277683235704899	Shared protein domains	INTERPRO PFAM
ESR2	NR1H4	0.04277683235704899	Shared protein domains	INTERPRO PFAM
RORA	NR1D1	0.04277236387133598	Shared protein domains	INTERPRO PFAM
RORA	NR2C1	0.04277236387133598	Shared protein domains	INTERPRO PFAM
RORC	NR1D1	0.04277236387133598	Shared protein domains	INTERPRO PFAM
RORC	NR2C1	0.04277236387133598	Shared protein domains	INTERPRO PFAM
NR2C2	RORA	0.04277236387133598	Shared protein domains	INTERPRO PFAM
NR2C2	RORC	0.04277236387133598	Shared protein domains	INTERPRO PFAM
ESRRA	RORA	0.04277236387133598	Shared protein domains	INTERPRO PFAM
ESRRA	RORC	0.04277236387133598	Shared protein domains	INTERPRO PFAM
NR1D2	RORA	0.04277236387133598	Shared protein domains	INTERPRO PFAM
NR1D2	RORC	0.04277236387133598	Shared protein domains	INTERPRO PFAM
RORA	ESR1	0.042772307991981506	Shared protein domains	INTERPRO PFAM
RORC	ESR1	0.042772307991981506	Shared protein domains	INTERPRO PFAM
PGR	RORA	0.042772307991981506	Shared protein domains	INTERPRO PFAM
PGR	RORC	0.042772307991981506	Shared protein domains	INTERPRO PFAM
ESR2	RORA	0.042772307991981506	Shared protein domains	INTERPRO PFAM
ESR2	RORC	0.042772307991981506	Shared protein domains	INTERPRO PFAM
PPARG	THRB	0.04273326136171818	Shared protein domains	INTERPRO PFAM
NR1I2	PPARG	0.04273326136171818	Shared protein domains	INTERPRO PFAM
NR1I3	PPARG	0.04273326136171818	Shared protein domains	INTERPRO PFAM
NR1D1	NR1H3	0.04260025918483734	Shared protein domains	INTERPRO PFAM
NR2C1	NR1H3	0.04260025918483734	Shared protein domains	INTERPRO PFAM
NR2C2	NR1H3	0.04260025918483734	Shared protein domains	INTERPRO

				PFAM
ESRRA	NR1H3	0.04260025918483734	Shared protein domains	INTERPRO PFAM
NR1D2	NR1H3	0.04260025918483734	Shared protein domains	INTERPRO PFAM
NR1H3	ESR1	0.042600203305482864	Shared protein domains	INTERPRO PFAM
PGR	NR1H3	0.042600203305482864	Shared protein domains	INTERPRO PFAM
ESR2	NR1H3	0.042600203305482864	Shared protein domains	INTERPRO PFAM
THRB	PPARA	0.04253241419792175	Shared protein domains	INTERPRO PFAM
NR1I2	PPARA	0.04253241419792175	Shared protein domains	INTERPRO PFAM
NR1I3	PPARA	0.04253241419792175	Shared protein domains	INTERPRO PFAM
THRB	PPARD	0.04253239370882511	Shared protein domains	INTERPRO PFAM
NR1I2	PPARD	0.04253239370882511	Shared protein domains	INTERPRO PFAM
NR1I3	PPARD	0.04253239370882511	Shared protein domains	INTERPRO PFAM
PPARG	NR1H4	0.0424048975110054	Shared protein domains	INTERPRO PFAM
RORA	PPARG	0.04240045323967934	Shared protein domains	INTERPRO PFAM
RORC	PPARG	0.04240045323967934	Shared protein domains	INTERPRO PFAM
NR1H3	PPARG	0.04223143681883812	Shared protein domains	INTERPRO PFAM
PPARA	NR1H4	0.04220723733305931	Shared protein domains	INTERPRO PFAM
PPARD	NR1H4	0.04220722056925297	Shared protein domains	INTERPRO PFAM
RORA	PPARA	0.04220283776521683	Shared protein domains	INTERPRO PFAM
RORC	PPARA	0.04220283776521683	Shared protein domains	INTERPRO PFAM
RORA	PPARD	0.042202819138765335	Shared protein domains	INTERPRO PFAM
RORC	PPARD	0.042202819138765335	Shared protein domains	INTERPRO PFAM
NR1D1	THRA	0.04216170869767666	Shared protein domains	INTERPRO PFAM
NR2C1	THRA	0.04216170869767666	Shared protein domains	INTERPRO PFAM
NR2C2	THRA	0.04216170869767666	Shared protein domains	INTERPRO PFAM
ESRRA	THRA	0.04216170869767666	Shared protein domains	INTERPRO PFAM
NR1D2	THRA	0.04216170869767666	Shared protein domains	INTERPRO PFAM
THRA	ESR1	0.04216165281832218	Shared protein domains	INTERPRO PFAM
PGR	THRA	0.04216165281832218	Shared protein domains	INTERPRO PFAM
ESR2	THRA	0.04216165281832218	Shared protein domains	INTERPRO PFAM
RXRG	NR1D1	0.04209457151591778	Shared protein domains	INTERPRO PFAM
RXRG	NR2C1	0.04209457151591778	Shared protein domains	INTERPRO PFAM

RXRG	NR2C2	0.04209457151591778	Shared protein domains	INTERPRO<br>PFAM
ESRRA	RXRG	0.04209457151591778	Shared protein domains	INTERPRO<br>PFAM
NR1D2	RXRG	0.04209457151591778	Shared protein domains	INTERPRO<br>PFAM
RXRG	ESR1	0.04209451377391815	Shared protein domains	INTERPRO<br>PFAM
PGR	RXRG	0.04209451377391815	Shared protein domains	INTERPRO<br>PFAM
ESR2	RXRG	0.04209451377391815	Shared protein domains	INTERPRO<br>PFAM
ESRRG	THRB	0.042083900421857834	Shared protein domains	INTERPRO<br>PFAM
ESRRG	NR1I2	0.042083900421857834	Shared protein domains	INTERPRO<br>PFAM
ESRRG	NR1I3	0.042083900421857834	Shared protein domains	INTERPRO<br>PFAM
NR1H3	PPARA	0.042035460472106934	Shared protein domains	INTERPRO<br>PFAM
NR1H3	PPARD	0.04203544370830059	Shared protein domains	INTERPRO<br>PFAM
RARG	NR1D1	0.04181999713182449	Shared protein domains	INTERPRO<br>PFAM
RARG	NR2C1	0.04181999713182449	Shared protein domains	INTERPRO<br>PFAM
RARG	NR2C2	0.04181999713182449	Shared protein domains	INTERPRO<br>PFAM
RARG	ESRRA	0.04181999713182449	Shared protein domains	INTERPRO<br>PFAM
RARG	NR1D2	0.04181999713182449	Shared protein domains	INTERPRO<br>PFAM
RARG	ESR1	0.041819941252470016	Shared protein domains	INTERPRO<br>PFAM
PGR	RARG	0.041819941252470016	Shared protein domains	INTERPRO<br>PFAM
ESR2	RARG	0.041819941252470016	Shared protein domains	INTERPRO<br>PFAM
THRA	PPARG	0.04180075228214264	Shared protein domains	INTERPRO<br>PFAM
ESRRG	NR1H4	0.0417658444494009	Shared protein domains	INTERPRO<br>PFAM
ESRRG	RORA	0.04176153987646103	Shared protein domains	INTERPRO<br>PFAM
ESRRG	RORC	0.04176153987646103	Shared protein domains	INTERPRO<br>PFAM
NR1D1	RXRB	0.041741929948329926	Shared protein domains	INTERPRO<br>PFAM
NR2C1	RXRB	0.041741929948329926	Shared protein domains	INTERPRO<br>PFAM
NR2C2	RXRB	0.041741929948329926	Shared protein domains	INTERPRO<br>PFAM
ESRRA	RXRB	0.041741929948329926	Shared protein domains	INTERPRO<br>PFAM
NR1D2	RXRB	0.041741929948329926	Shared protein domains	INTERPRO<br>PFAM
RXRB	ESR1	0.0417418722063303	Shared protein domains	INTERPRO<br>PFAM
PGR	RXRB	0.0417418722063303	Shared protein domains	INTERPRO<br>PFAM
ESR2	RXRB	0.0417418722063303	Shared protein domains	INTERPRO<br>PFAM

RXRG	PPARG	0.04172775335609913	Shared protein domains	INTERPRO PFAM
NR1D1	NR1H2	0.04169541597366333	Shared protein domains	INTERPRO PFAM
NR2C1	NR1H2	0.04169541597366333	Shared protein domains	INTERPRO PFAM
NR2C2	NR1H2	0.04169541597366333	Shared protein domains	INTERPRO PFAM
ESRRA	NR1H2	0.04169541597366333	Shared protein domains	INTERPRO PFAM
NR1D2	NR1H2	0.04169541597366333	Shared protein domains	INTERPRO PFAM
ESR1	NR1H2	0.041695361956954	Shared protein domains	INTERPRO PFAM
PGR	NR1H2	0.041695361956954	Shared protein domains	INTERPRO PFAM
ESR2	NR1H2	0.041695361956954	Shared protein domains	INTERPRO PFAM
THRA	PPARA	0.04160895571112633	Shared protein domains	INTERPRO PFAM
THRA	PPARD	0.041608940809965134	Shared protein domains	INTERPRO PFAM
ESRRG	NR1H3	0.04159782640635967	Shared protein domains	INTERPRO PFAM
RXRG	PPARA	0.041532840579748154	Shared protein domains	INTERPRO PFAM
RXRG	PPARD	0.04153282009065151	Shared protein domains	INTERPRO PFAM
RARG	PPARG	0.04146517068147659	Shared protein domains	INTERPRO PFAM
RXRB	PPARG	0.0413885023444891	Shared protein domains	INTERPRO PFAM
NR1H2	PPARG	0.04134282469749451	Shared protein domains	INTERPRO PFAM
RARG	PPARA	0.041276633739471436	Shared protein domains	INTERPRO PFAM
RARG	PPARD	0.04127661697566509	Shared protein domains	INTERPRO PFAM
RXRB	PPARA	0.04120070859789848	Shared protein domains	INTERPRO PFAM
RXRB	PPARD	0.04120069183409214	Shared protein domains	INTERPRO PFAM
NR1H2	PPARA	0.04115547426044941	Shared protein domains	INTERPRO PFAM
NR1H2	PPARD	0.041155459359288216	Shared protein domains	INTERPRO PFAM
ESRRG	RXRG	0.04109758138656616	Shared protein domains	INTERPRO PFAM
RXRG	THRB	0.04078689590096474	Shared protein domains	INTERPRO PFAM
RXRG	NR1I2	0.04078689590096474	Shared protein domains	INTERPRO PFAM
RXRG	NR1I3	0.04078689590096474	Shared protein domains	INTERPRO PFAM
RARB	NR1D1	0.040732767432928085	Shared protein domains	INTERPRO PFAM
RARB	NR2C1	0.040732767432928085	Shared protein domains	INTERPRO PFAM
RARB	NR2C2	0.040732767432928085	Shared protein domains	INTERPRO PFAM
RARB	ESRRA	0.040732767432928085	Shared protein domains	INTERPRO PFAM

RARB	NR1D2	0.040732767432928085	Shared protein domains	INTERPRO PFAM
RARB	ESR1	0.04073271155357361	Shared protein domains	INTERPRO PFAM
PGR	RARB	0.04073271155357361	Shared protein domains	INTERPRO PFAM
ESR2	RARB	0.04073271155357361	Shared protein domains	INTERPRO PFAM
RARG	THRB	0.0405550803989172	Shared protein domains	INTERPRO PFAM
RARG	NR1I2	0.0405550803989172	Shared protein domains	INTERPRO PFAM
RARG	NR1I3	0.0405550803989172	Shared protein domains	INTERPRO PFAM
RXRG	NR1H4	0.04048316739499569	Shared protein domains	INTERPRO PFAM
RXRB	THRB	0.04048200510442257	Shared protein domains	INTERPRO PFAM
RXRB	NR1I2	0.04048200510442257	Shared protein domains	INTERPRO PFAM
NR1I3	RXRB	0.04048200510442257	Shared protein domains	INTERPRO PFAM
RXRG	RORA	0.04047905653715134	Shared protein domains	INTERPRO PFAM
RXRG	RORC	0.04047905653715134	Shared protein domains	INTERPRO PFAM
RARB	PPARG	0.04039744287729263	Shared protein domains	INTERPRO PFAM
RXRG	NR1H3	0.040322719141840935	Shared protein domains	INTERPRO PFAM
RARG	NR1H4	0.04026128351688385	Shared protein domains	INTERPRO PFAM
RARG	RORA	0.04025730863213539	Shared protein domains	INTERPRO PFAM
RARG	RORC	0.04025730863213539	Shared protein domains	INTERPRO PFAM
RARB	PPARA	0.040219271555542946	Shared protein domains	INTERPRO PFAM
RARB	PPARD	0.0402192547917366	Shared protein domains	INTERPRO PFAM
RXRB	NR1H4	0.04018936678767204	Shared protein domains	INTERPRO PFAM
RORA	RXRB	0.04018540680408478	Shared protein domains	INTERPRO PFAM
RORC	RXRB	0.04018540680408478	Shared protein domains	INTERPRO PFAM
RARG	NR1H3	0.04010608233511448	Shared protein domains	INTERPRO PFAM
RXRB	NR1H3	0.04003477841615677	Shared protein domains	INTERPRO PFAM
RXRG	THRA	0.03992434777319431	Shared protein domains	INTERPRO PFAM
RXRG	NR1H2	0.039500774815678596	Shared protein domains	INTERPRO PFAM
KDR	FLT1	0.038302602246403694	Shared protein domains	INTERPRO PFAM
MAPKAPK2	MAPK14	0.015003179665654898	Shared protein domains	INTERPRO PFAM
MAPKAPK2	MAPK10	0.014965989161282778	Shared protein domains	INTERPRO PFAM
MAPK14	MAPK10	0.014837779570370913	Shared protein domains	INTERPRO PFAM
MAPKAPK2	GSK3B	0.006023443071171641	Shared protein domains	INTERPRO

				PFAM
MAPKAPK2	CDK9	0.006023443071171641	Shared protein domains	INTERPRO PFAM
MAPKAPK2	CDK2	0.006015064660459757	Shared protein domains	INTERPRO PFAM
MAPK14	GSK3B	0.005969909252598882	Shared protein domains	INTERPRO PFAM
MAPK14	CDK9	0.005969909252598882	Shared protein domains	INTERPRO PFAM
CDK2	MAPK14	0.00596166867762804	Shared protein domains	INTERPRO PFAM
MAPK10	GSK3B	0.005955187603831291	Shared protein domains	INTERPRO PFAM
CDK9	MAPK10	0.005955187603831291	Shared protein domains	INTERPRO PFAM
CDK2	MAPK10	0.0059469640254974365	Shared protein domains	INTERPRO PFAM
CDK9	GSK3B	0.004580185515806079	Shared protein domains	INTERPRO PFAM
CDK2	GSK3B	0.004574882099404931	Shared protein domains	INTERPRO PFAM
CDK2	CDK9	0.004574882099404931	Shared protein domains	INTERPRO PFAM

Search results generated by the GeneMANIA algorithm (genemania.org)
